# From test to rest: evaluating socioeconomic differences along the COVID-19 care pathway in the Netherlands

**DOI:** 10.1007/s10198-024-01680-4

**Published:** 2024-03-18

**Authors:** Iris Meulman, Ellen Uiters, Mariëlle Cloin, Jeroen Struijs, Johan Polder, Niek Stadhouders

**Affiliations:** 1 Tranzo, Tilburg School of Social and Behavioral Sciences, Tilburg University, Tilburg, The Netherlands; 2 Center for Public Health, Healthcare & Society, National Institute for Public Health and the Environment, P.O. Box 1, 3720 BA Bilthoven, The Netherlands; 3 Center for Prevention, Lifestyle and Health, National Institute for Public Health and the Environment, Bilthoven, The Netherlands; 4 Department of Public Health and Primary Care, Leiden University Medical Center–Health Campus The Hague, The Hague, The Netherlands; 5 Scientific Center for Quality of Healthcare, Radboud University Medical Center, Nijmegen, The Netherlands

**Keywords:** Socioeconomic inequality, COVID-19 testing, COVID-19 healthcare utilization, COVID-19 mortality, I14, I18

## Abstract

**Introduction:**

The COVID-19 pandemic exacerbated healthcare needs and caused excess mortality, especially among lower socioeconomic groups. This study describes the emergence of socioeconomic differences along the COVID-19 pathway of testing, healthcare use and mortality in the Netherlands.

**Methodology:**

This retrospective observational Dutch population-based study combined individual-level registry data from June 2020 to December 2020 on personal socioeconomic characteristics, COVID-19 administered tests, test results, general practitioner (GP) consultations, hospital admissions, Intensive Care Unit (ICU) admissions and mortality. For each outcome measure, relative differences between income groups were estimated using log-link binomial regression models. Furthermore, regression models explained socioeconomic differences in COVID-19 mortality by differences in ICU/hospital admissions, test administration and test results.

**Results:**

Among the Dutch population, the lowest income group had a lower test probability (RR = 0.61) and lower risk of testing positive (RR = 0.77) compared to the highest income group. However, among individuals with at least one administered COVID-19 test, the lowest income group had a higher risk of testing positive (RR = 1.40). The likelihood of hospital admissions and ICU admissions were higher for low income groups (RR = 2.11 and RR = 2.46, respectively). The lowest income group had an almost four times higher risk of dying from COVID-19 (RR = 3.85), which could partly be explained by a higher risk of hospitalization and ICU admission, rather than differences in test administration or result.

**Discussion:**

Our findings indicated that socioeconomic differences became more pronounced at each step of the care pathway, culminating to a large gap in mortality. This underlines the need for enhancing social security and well-being policies and incorporation of health equity in pandemic preparedness plans.

**Supplementary Information:**

The online version contains supplementary material available at 10.1007/s10198-024-01680-4.

## Introduction

The COVID-19 pandemic impacted many aspects of the health system, pressuring hospital capacity and causing high excess mortality. In 2020, the Netherlands registered more than 20,000 deaths due to COVID-19 [1] and an overall excess mortality of more than 15,000 deaths [2]. Furthermore, over 30,000 individuals were admitted to Dutch hospitals with COVID-19 [3], of which approximately 6500 were admitted to the ICU [4]. This has had a major impact on regular care provision, both in hospitals and primary care [5, 6]. Regular care was postponed, canceled, avoided or provided digitally and waiting times increased [7–9]. GPs function as gatekeepers in the Dutch healthcare system. During the COVID-19 pandemic, GPs had to transform and adapt service provision due to a large influx of patients and imposed contact restrictions. Being the first point of contact for patients, GPs experienced a large influx of patients with COVID-19 related complaints and patients whose treatment was postponed in secondary care. Furthermore, impose contact restrictions forced GPs to mainly provide digital care. These changes in primary care increased workload and put pressure on the accessibility and quality of care during the COVID-19 pandemic [10–12]. To prevent spread and potential care use related to COVID-19, Municipal Health Services offered free testing in case of COVID-19 related symptoms from June 1st 2020 onwards [13]. Although free of charge, individuals could have experienced barriers for barriers for testing, which included capacity constraints and long waiting times, accessibility of the test location, digital barriers in making appointments and recognizing health symptoms [14]. On the other hand, being free of charge could have induced risky behavior (i.e., moral hazard) and overconsumption of testing.

Socioeconomic Status (SES) disparities in health status, health care utilization and mortality were prominent before the pandemic. Although various definitions and interpretations exists, SES in health research is a multidimensional construct that encompasses individual’s or group’s access to basic resources required to achieve and maintain good health [15]. Among an adult population, SES is often determined by factors such as income, assets or equity, education, occupation and employment, each covering various aspects of SES [16]. Additionally, SES indicators may comprise other sociocultural differences, such as lifestyle, behavioral, cultural, and contextual factors. Regardless of level of healthcare coverage in countries, individuals with lower SES reported poorer health and used on average more care [17, 18]. This association was more ambiguous for previous pandemics and infectious diseases, as it varied by (respiratory) infectious disease whether it affected socioeconomic groups differently [19–22]. International evidence on socioeconomic differences in COVID-19 infections, utilization and mortality is emerging rapidly [23, 24]. In the Netherlands, preliminary figures show that low education is negatively correlated to test rates for COVID-19 via the Municipal Health Services [25]. Furthermore, low-income individuals had higher risk of dying from COVID-19 [26, 27]. Findings from other European countries with universal healthcare systems consistently showed that lower socioeconomic groups had a higher risk for COVID-19 related hospital admission, ICU admission and mortality than higher socioeconomic groups among the general population, as well as among the population with at least one administered test and confirmed COVID-19 cases [28–31]. Findings were, however, mixed for positive COVID-19 tests. In the general population, both no differences as well as a lower risk of testing positive for COVID-19 for individuals with lower SES compared with higher SES were found [28–30]. For England, Thygesen, Tomlinson, Hollings et al. [32] constructed a COVID-19 care pathway analysis, where care pathways of each individual were followed, including stages of no COVID-19 infection, positive COVID-19 test, primary care COVID-19 diagnosis, hospitalization for COVID-19, ICU admission for COVID-19 and death due to COVID-19. They stratified their analysis, among others, by area index of multiple deprivation (IMD), which is a combined measure of area income, employment, education, health, crime, barriers to housing and services, and living environment [33]. Comparing the lowest and highest area IMD scores, individuals with the lowest IMD score had higher transition rates between most stages, but there were no major differences in elapsed time between stages between lowest and highest area IMD score. When focusing on a hospitalized population, a US and English study showed that lower SES, in terms of median household income or area deprivation index respectively, was associated with higher in-hospital mortality with COVID-19 [34, 35]. The study periods comprised 2020 for the US study and March 1 to May 31, 2020 for the English study. Contrarily, a Scottish study found no differences between Scottish area IMD in mortality among hospitalized patients with COVID-19 during the first wave in 2020 [36].

**Fig. 1:**
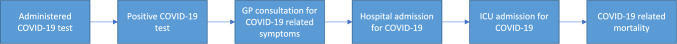
COVID-19 care pathway. Note that an individual does not have to use care in each step and does not necessarily have to follow the care pathway linearly

To date, a comprehensive overview of socioeconomic differences along the COVID-19 care pathway, from testing to mortality, is scarce. The majority of research studies concentrated on a restricted set of outcome measures. We included six consecutive steps in the pathway: administered COVID-19 tests, positive COVID-19 tests, contact with GP for COVID-19, hospital admission for COVID-19, ICU admission for COVID-19 and COVID-19 mortality (Fig. 1). Consequently, the addition of this study to existing literature resides in its comprehensive analysis of the entire care pathway and specifically the inclusion of GP consultations for COVID-19 related health symptoms. Furthermore, most studies employed geographically aggregated data as a proxy for socioeconomic status, as individual level data pertaining to for example income, education and wealth may either be lacking of insufficiently widespread. This study addresses this research gap by utilizing nationwide individual level data encompassing both socioeconomic status and COVID-19 outcome measures. To contribute to the current knowledge base, we aim to answer the following questions:

1. To what extent did COVID-19 related testing, healthcare utilization and mortality differ by socioeconomic groups?
2. To what extent could socioeconomic differences in COVID-19 related mortality be explained by differences in COVID-19 related testing and healthcare utilization?

To answer the first research question, we used binomial regression models with log-link and adjusted step-by-step for age, sex, health status, degree of urbanization of the living environment, household size and country of origin. The second research question will be answered using binomial regression models with log-link and adjusted step-by-step for COVID-19 administered tests, positive tests, hospital admission and ICU admission.

## Methodology

### Study population and period

The study population included all Dutch residents on January 1, 2020 living in a non-institutionalized household and aged 25–79 years. The study period covered June to December 2020, because all outcomes measures were collectively available during this period. The study therefore covered a period of relatively low infection rates and relaxation of COVID-19 lockdown and other measures (June to October) and the ‘second wave’ of infections with increasing restrictions to a complete lockdown (October to December) [13]. The primary variant of SARS-COV-2 was dominant during this times [37]. In the Netherlands, COVID-19 vaccinations became available from January 2021, therefore the effect of vaccination was not taken into account in this study.

### Study design and data sources

This cross-sectional study used pseudonymized routinely collected registry data at the individual level. Individual characteristics, i.e., age, sex, income, education, country of origin, urbanity and household size, from Statistic Netherlands and hospital visits from Vektis (a healthcare business intelligence center who combines and provides declared healthcare data) were linked at individual level to six different types of COVID-19-related outcome measures: administered COVID-19 tests and positive tests from the municipal health services, GP registration data from the Extramural Leiden University Medical Center Academic Network (ELAN), hospital and ICU admissions from Dutch Hospital Data (DHD) and mortality registrations from Statistics Netherlands. Statistics Netherlands functioned as a trusted third party, enabling the linkage between datasets, while ensuring the privacy of the involved individuals, according to Dutch law (Statistics Netherlands Act 2003). An overview of the variables, data sources and population is provided in Table 1.

**Table 1:** Variable characteristics

Variable	Data source	Population	Coded
COVID-19 outcome measures			
Administered tests	Municipal health services	Dutch population	Binary (yes/no)
Positive tests	Municipal health services	Dutch population	Binary (yes/no)
GP-consultations	ELAN	Haaglanden region	Binary (yes/no)
Hospital admissions	Dutch Hospital Data	Dutch population	Binary (yes/no)
ICU admissions	Dutch Hospital Data	Dutch population	Binary (yes/no)
Mortality	Statistics Netherlands	Dutch population	Binary (yes/no)
Socioeconomic characteristics			
Disposable household income	Statistics Netherlands (via tax filings)	Dutch population	Categorical (deciles)
Education	Statistics Netherlands	Dutch population	Categorical (low/middle/high)
Financial wealth	Statistics Netherlands (via tax filings)	Dutch population	Categorical (deciles)
Covariates			
Age	Statistics Netherlands	Dutch population	Categorical (25–44/45–64/65–79)
Sex	Statistics Netherlands	Dutch population	Categorical (male/female)
Elixhauser Comorbidity Index	Vektis	Dutch population	Binary (yes/no)
Urbanity	Statistics Netherlands	Dutch population	Categorical (1–6)
Household size	Statistics Netherlands	Dutch population	Categorical (1/2/3–4/> 4)
Country of origin	Statistics Netherlands	Dutch population	Categorical (Dutch/European/Other)

#### COVID-19 tests

The number of administered tests for COVID-19 in all Municipal Health Services test locations were available from June 2020 to December 2020. Administered COVID-19 tests and positive test results were binary coded, indicating at least one administered or positive test at the Municipal Health Services, respectively, between June and December 2020.

#### COVID-19-related GP-consultations

Data from the ELAN network were used to identify COVID-19-related GP consultations [38]. This network includes registrations of about 100 general practitioners in The Hague greater area (5% of the Dutch population), enrolling approximately 300,000 Dutch citizens. Following Sijbom, Büchner, Saadah et al. [39], COVID-19-related GP consultations were identified as consultations with ICPC code R74 or an ICPC code associated with COVID-19-related symptoms together with an indication of COVID-19 in the description (Appendix 1). COVID-19-related GP consultations were coded binary, with score one indicating at least one COVID-19-related GP consultation between June and December 2020.

#### COVID-19-related hospitalization

DHD provided data on COVID-19-related hospitalizations in nursing wards and ICUs. Hospitalizations related to COVID-19 were identified as hospitalization with ICD-10 codes U07.1 (confirmed COVID-19) or U07.2 (suspected COVID-19) [40]. Due to the structure of the data, it was not possible to distinguish whether a patient had been admitted to the hospital due to health complaints caused by a COVID-19 infection or had been admitted for other health complaints while having COVID-19. COVID-19-related hospital and ICU admissions and were coded binary, indicating at least one admission to the hospital or ICU with COVID-19 respectively between June-December 2020.

#### COVID-19 mortality

Mortality with COVID-19 (ICD-10 code U07.1) or suspected COVID-19 (ICD-10 code U07.2) was based on the official mortality registry of Statistics Netherlands [41]. This registry uses the primary cause of death as identified by the physician.

#### Socioeconomic status: income, educational level and financial wealth

We used disposable household income, standardized for household composition [42], as the main indicator for socioeconomic status since household income is most frequently used and arguably the best single indicator of material living standards [16]. To facilitate interpretability, we categorized income level. Income was categorized through a data-driven approach, employing scatterplots to determine the most appropriate cut-off points, thereby resulting into decile categorization. The income deciles were age-standardized, implying that the income decile is based on the income distribution of peers from the same age category (5-year categories for the population aged 25 to 79). This implies that a different income threshold value applied in different age groups to belong, for example, to the lowest income group.

We additionally used educational level [43], lagged income, and financial wealth, derived from tax filings from the Dutch Tax Administration [44], to check for robustness across SES-indicators. These findings are presented in Appendix 3. For educational level, following Statistics Netherlands, the highest level of completed level of education is used and categorized as low (lower vocational educational level, lower secondary educational level or less), middle (intermediate vocational educational level or higher secondary educational level) or high (higher vocational educational level or university). Educational level was missing for a substantial part of the population (38.6%) and missing values were strongly correlated with age. Therefore, a random forest approach, based on data from the representative Dutch Labor Force Survey, was used to impute missing information on educational level. Financial wealth was defined as the difference between households assets and household debts and standardized for household composition. Financial wealth was, using the same approach as for income, categorized in deciles relative to peers in the same 5-year age group.

#### Covariates

Age, sex, elixhauser comorbidity index, urbanity of living environment, household size and country of origin were included as covariates. These covariates were included because they are known to be associated with income and one or more of the outcome variables [45–51]. The covariates were maintained across models for consistency. Age was measured on January 1, 2020, and classified into categories to improve interpretability. Applying a data-driven approach to determine the optimal cut-off points, age was categorized into two age categories (25–64 and 65–79) for models with mortality as outcome measure, and three age categories (25–44, 45–64 and 65–79) for models with other outcome measures. The elixhauser comorbidity index is used as a proxy for health status. The elixhauser comorbidity index is operationalized based on prior healthcare use and indicated whether one had one or more hospital visits (both inpatient or outpatient) between 2016 and 2019 for one of the comorbidity listed in the Elixhauser index. This index identifies 30 comorbidities that are associated with substantial increases in length of stay, hospital charges, and mortality [52] and that are closely related to underlying health conditions marked by the Dutch Institute for Public Health and the Environment (RIVM) as risk groups for severe illness from COVID-19 [53]. Primary diagnoses of individual hospital visits from 2016 to 2019 were identified from claims data [54] using algorithms developed during cost-of-illness studies [55] and classified based on the list of Elixhauser ICD-10 codes (Appendix 2). For urbanity six categories were distinguished based on neighborhood address density (0–500 (least urban), 500–1000, 1000–1500, 1500–2500, 2500–5000 or more than 5000 (most urban) addresses per squared kilometer). Household size was defined as a single household, two-person household, three- or four-person household or more than four-person household. Country of origin was defined as Dutch origin, first or second generation of European country origin (excluding the Netherlands) or first or second generation from a non-European country.

### Statistical analyses

Descriptive statistics are provided for the study sample of the Dutch population and The Hague greater area population aged 25–79. Socioeconomic differences were estimated per outcome measure for the Dutch population using binomial regression models with log-link to obtain relative risks (RR) of each income decile compared to the highest income decile. For the analysis on GP consultations, the sample was limited to citizens enrolled at one of the participating GP practices. Additionally, the ratio of positive COVID-19 tests was assessed on the Dutch population with at least one administered test and the ratio of COVID-19-related mortality was assessed on the Dutch hospitalized population. All models were fitted using stepwise inclusion of covariates. We decided to use the same covariates for each outcome measure to increase comparability.

- Model 1: SES (income/education/financial wealth) + age + sex
- Model 2: Model 1 + Elixhauser comorbidity index
- Model 3: Model 2 + urbanity + household size + country of origin

Finally, we used multiple logistic regression models to explore to which extent socioeconomic differences in COVID-19 mortality could be explained by differences in hospital admissions, testing behavior and testing results. Here, model 2 was used with COVID-19 mortality as an outcome measure, followed by a stepwise inclusion following the care pathway (administered COVID-19 tests, positive COVID-19 tests, hospital admission for COVID-19 and ICU admission for COVID-19 respectively). GP consultations were not included because they were not available for the entire Dutch population. The fitted models were:

- Model 4: Model 2 + administered COVID-19 tests
- Model 5: Model 4 + positive COVID-19 tests
- Model 6: Model 5 + hospital admissions for COVID-19
- Model 7: Model 6 + ICU admissions for COVID-19

To test for robustness across SES-indicators, we repeated the analyses using educational attainment, lagged income, and financial wealth instead of income. Confidence intervals (CIs) were set at 95%. Analyses were conducted in R version 4.1.2, using the logbin package [56].

## Results

### Study population

Data on sex, age, standardized disposable household income, household financial wealth, urbanity and household size were available for 11,447,803 individuals aged 25–79 in the Dutch population (3.9% of individuals excluded due to missing data). 226,959 of these individuals were enrolled at one of the participating GP practices (2.0% of the Dutch population). Table 2 shows the characteristics of both study populations. Approximately half of the Dutch population was female (50.3%) and the mean age was 50.8 (SD 15.0). Three-quarters of the population had a Dutch country of origin, 8% had another European background and 16% had a non-European country of origin. Most individuals lived in a two-person household and in an area with 1500–2500 addresses per km^2^. Almost half of the population was identified with one or more Elixhauser comorbidities (44.4%).

2% of the full Dutch study population was enrolled at one of the participating GP practices in The Hague greater area. Compared to the full Dutch population, individuals in The Hague greater area that enrolled at participating GP practices had similar age, sex, income, education, financial wealth and Elixhauser comorbidity distribution. Individuals in The Hague greater area did have a non-European country of origin more often, lived in more urban areas, and lived in larger households (Table 2).

**Table 2:** Descriptive information of (sample) populations

Variable	The Netherlands	The Hague greater area
	% (*N*)	% (*N*)
Total (aged 25–79)	100 (11,477,803)	100 (226,959)
Sex		
Male	49.7 (5,699,393)	48.3 (109,662)
Female	50.3 (5,778,410)	51.7 (117,297)
Age		
25–44	36.4 (4,176,820)	38.2 (86,728)
45–64	41.6 (4,770,451)	40.3 (91,384)
65–79	22.0 (2,530,532)	21.5 (48,847)
Country of origin		
Dutch origin	76.8 (8,818,801)	69.5 (157,689)
European origin	7.6 (869,854)	6.5 (14,841)
Other	15.6 (1,789,148)	24.0 (54,429)
Disposable household income^a^		
1st decile	10.0 (1,147,612)	10.2 (23,228)
2nd decile	10.0 (1,147,717)	9.7 (22,024)
3rd decile	10.0 (1,147,781)	9.6 (21,677)
4th decile	10.0 (1,147,735)	9.6 (21,692)
5th decile	10.0 (1,147,877)	9.6 (21,890)
6th decile	10.0 (1,147,816)	10.0 (22,636)
7th decile	10.0 (1,147,840)	10.2 (23,161)
8th decile	10.0 (1,147,838)	10.3 (23,444)
9th decile	10.0 (1,147,796)	10.7 (24,173)
10th decile	10.0 (1,147,791)	10.1 (23,034)
Highest level of completed educational attainment		
Lower education	12.8 (1,466,022)	13.9 (31,651)
Secondary education	23.8 (2,731,133)	24.0 (54,539)
Higher education	24.8 (2,847,491)	24.0 (54,525)
Missing (before imputation)	38.6 (4,433,157)	38.0 (86,244)
Household financial wealth^a^		
1st decile	10.0 (1,147,725)	10.9 (24,829)
2nd decile	10.0 (1,147,725)	11.2 (25,468)
3rd decile	10.0 (1,147,569)	10.6 (24,152)
4th decile	10.0 (1,147,604)	9.6 (21,855)
5th decile	10.0 (1,147,730)	9.4 (21,351)
6th decile	10.0 (1,147,817)	9.7 (21,976)
7th decile	10.0 (1,147,851)	10.5 (23,753)
8th decile	10.0 (1,147,885)	10.4 (23,687)
9th decile	10.0 (1,147,872)	9.4 (21,250)
10th decile	10.0 (1,147,876)	8.2 (18,638)
Urbanity (number of addresses per km^2^)		
0–500 per km^2^ (least urban)	14.9 (1,715,126)	0.7 (1,566)
500–1000 per km^2^	16.8 (1,925,077)	1.5 (3,310)
1000–1500 per km^2^	17.9 (2,056,642)	3.7 (8,385)
1500–2500 per km^2^	27.4 (3,142,603)	43.8 (99,404)
2500–5000 per km^2^	17.1 (1,958,318)	39.7 (90,099)
> 5000 per km^2^ (most urban)	5.9 (680,037)	10.7 (24,195)
Elixhauser comorbidity index		
No elixhauser comorbidity	55.0 (6,314,704)	53.0 (120,272)
One or more elixhauser comorbidities	45.0 (5,163,099)	47.0 (106,687)
Household size		
1	20.1 (2,307,387)	21.1 (47,788)
2	38.9 (4,461,100)	37.1 (84,107)
3–4	33.3 (3,824,923)	33.5 (76,029)
5 or more	7.7 (884,393)	8.4 (19,035)

^a^Standardized for household composition relative to peers in the same 5-year age group. Percentages per decile deviate from 10% in The Hague greater area because a subsample of the Dutch population was taken

### Descriptive statistics of the outcome measures

Table 3 shows descriptive information on the six outcome measures among the Dutch population and The Hague greater area. In the Dutch population, 27.7% of the individuals between 25 and 79 years old took at least one COVID-19 test at the Municipality Health Services during the period June 1st to December 31st 2020. This percentage ranged between 17.5% for the lowest income decile and 30.4% for the highest income decile. Furthermore, 3.8% of the Dutch population tested positive for COVID-19. Among the subpopulation living in the Hague greater area, 4.68% consulted the GP for COVID-19-related symptoms. In total, 0.13% in the Dutch population had been admitted to the hospital with COVID-19 or suspicion of COVID-19, of which 18% had been admitted to the ICU. Mortality rates ranged from 0.013% among the highest income decile to 0.059% in the lowest income decile, with an average of 0.026%.

**Table 3:** Descriptive information on COVID-19-related outcome measures per income decile among the Dutch population (*N* = 11,477,803)

Income decile	Administered tests	Positive tests	GP consultation (the Hague greater area)	Hospital admission	ICU admission	Mortality
1	17.500% (200,351)	2.842% (32,611)	4.279% (994)	0.247% (2,835)	0.049% (562)	0.059% (673)
2	23.068% (264,758)	3.350% (38,447)	4.631% (1020)	0.179% (2,055)	0.030% (345)	0.036% (414)
3	26.139% (300,023)	3.696% (42,420)	4.858% (1053)	0.151% (1,730)	0.026% (297)	0.029% (334)
4	27.735% (318,327)	3.876% (44,489)	4.831% (1048)	0.130% (1,495)	0.022% (247)	0.025% (286)
5	28.921% (331,974)	4.014% (46,077)	4.742% (1038)	0.123% (1,415)	0.023% (267)	0.023% (260)
6	29.865% (342,797)	4.093% (46,981)	5.116% (1158)	0.123% (1,408)	0.021% (245)	0.023% (265)
7	30.679% (352,144)	4.119% (47,279)	5.082% (1177)	0.104% (1,197)	0.018% (212)	0.020% (227)
8	31.287% (359,119)	4.105% (47,122)	4.833% (1133)	0.098% (1,124)	0.017% (195)	0.017% (194)
9	31.494% (361,485)	3.903% (44,794)	4.501% (1088)	0.089% (1,026)	0.017% (196)	0.012% (139)
10	30.427% (349,240)	3.551% (40,758)	3.925% (904)	0.083% (956)	0.014% (159)	0.013% (150)
Total	27.708% (3,180,218)	3.755% (430,978)	4.676% (10,613)	0.133% (15,241)	0.024% (2,725)	0.026% (2,942)

GP consultations among the Hague greater area (*n* = 226,959). June to December 2020. Frequency in between brackets

### Socioeconomic differences in COVID-19-related healthcare use and mortality

**Fig. 2:**
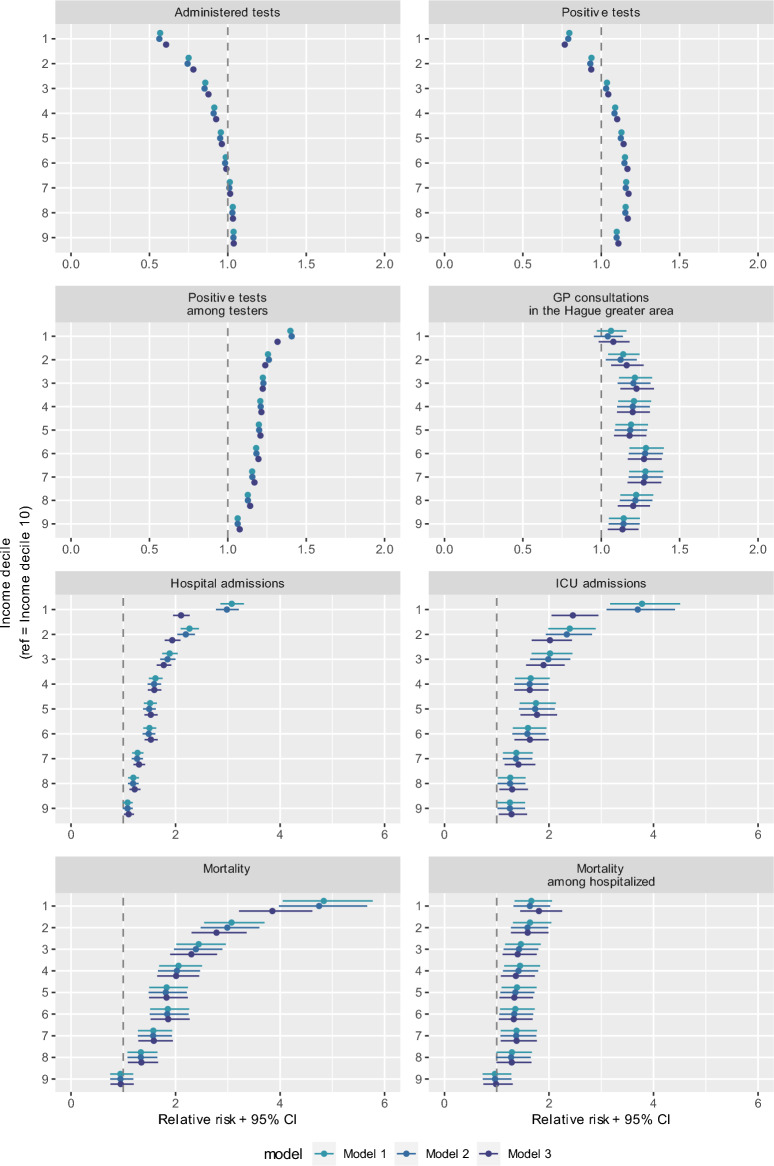
Socioeconomic differences in COVID-19-related testing, healthcare use and mortality. Reference category is income decile 10 (highest income group). Model 1: SES (income) + age + sex; Model 2: Model 1 + elixhauser index; Model 3: Model 2 + urbanity + household size + country of origin (fully adjusted). Analyses with outcomes administered tests, positive tests, hospital admissions, ICU admissions and mortality are analyzed on the Dutch population (N = 11,477,803). GP consultations were analyzed among the Hague greater area (N = 226,959), positive tests among testers was analyzed on the Dutch population with at least one administered test (n = 3,180,218) and mortality among hospitalized was analyzed among the Dutch hospitalized population (n = 15,241). Please note the different x-axis scales

Figure 2 shows for each outcome measure the differences between income groups compared to the highest income group (see Appendix 3 for tables with all regression coefficients). Results reported below refer to the relative risk of the lowest income group compared to the highest income group from the fully adjusted model (model 3), unless stated otherwise. Individuals in the lowest income group had a 39% lower risk of at least one administered COVID-19 test (RR = 0.61, 95%CI 0.60–0.61) and in general a 23% lower risk of testing positive for COVID-19 (RR = 0.77, 95%CI 0.76–0.78) compared to individuals in the highest income group. However, among those who took at least one COVID-19 test, individuals in the lowest income group had a 32% higher relative risk of testing positive (RR = 1.32, 95%CI 1.30–1.34). In other words, individuals with lower income were at a population level less likely to test positive for COVID-19 most likely because they tested less often. Middle incomes went to the GP slightly more often with COVID-19-related complaints compared to the highest income group (income decile 6 vs. income decile 10; RR = 1.27, 95%CI 1.17–1.39), however, there was no statistically significant risk difference between the lowest and highest income groups (RR = 1.08, 95%CI 0.98–1.18). Individuals in the lowest income group had a 111% higher risk to be admitted to the hospital ward (RR = 2.11, 95%CI 1.95–2.27), 146% higher risk to be admitted to the ICU (RR = 2.46, 95%CI 2.05–2.95) and 285% higher risk to die with COVID-19 (RR = 3.85, 95%CI 3.21–4.62) compared to individuals in the highest income group. Among hospitalized patients, individuals in the lowest income group had a 81% higher risk to die with COVID-19 (RR = 1.81, 95%CI 1.45–2.25). These relative risk should be interpreted in the context of the small absolute numbers of hospitalization, ICU admission and mortality, as described in Table 3.The addition of covariates (elixhauser comorbidity index, urbanity, household size and country of origin) only marginally explained the association between income groups and differences (models 3 compared to models 1 and 2).

### Explaining income differences in COVID-19-related mortality

**Fig. 3:**
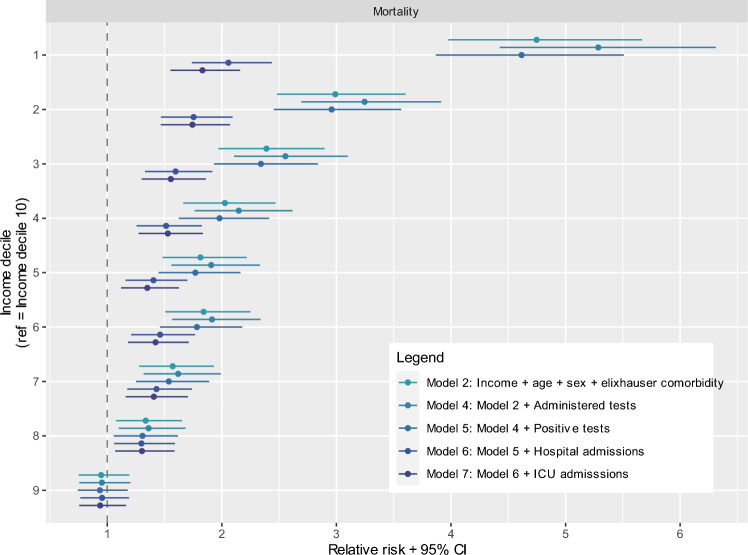
Explaining socioeconomic differences in COVID-19-related mortality

Figure 3 shows to what extent the higher risk of dying from COVID-19 among lower income groups can be explained by differences in testing and hospital admission (see Appendix 3 for tables with all regression coefficients). Here, model 2 (see also Fig. 2) with COVID-19-related mortality as outcome measure was extended step-by-step with administered COVID-19 tests, positive COVID-19 tests, hospital admission for COVID-19 and ICU admission for COVID-19. The higher risk of dying from COVID-19 among lower income individuals could only partly be explained by differences in hospital admission and ICU admission. After controlling for differences in testing and hospital/ICU admission, individuals in the lowest income group had a 83% higher risk of dying with COVID-19 (RR = 1.83, 95%CI 1.55–2.16, model 7) compared to individuals in the highest income group. Thus, the higher risk of COVID-19 mortality for lower-income individuals could partly be explained by a higher risk of COVID-19 hospitalization or ICU admission and partly by a higher risk of death when being hospitalized. However, income differences in COVID-19 mortality did not change when controlling for testing positive for COVID-19 or having at least one administered COVID-19 test (models 4 and 5). This indicates that differences in testing behavior and test results did not contribute to the observed socioeconomic differences in COVID-19-related mortality.

### Education, lagged income, and financial wealth as SES-indicator

Appendix 3 shows the results for financial wealth, lagged income, education and imputed education as a measure of SES. Results are in general consistent with the analysis using income as SES-indicator. The association of financial wealth with hospital admissions, ICU admissions and mortality seems to be slightly stronger compared to income.

## Discussion

In this retrospective cross-sectional observational study in the Netherlands, we evaluated socioeconomic differences in each step of the COVID-19 care pathway using individual level registration data. We found that lower income groups had a lower risk of having at least one administered COVID-19 test and testing positive for COVID-19. However, when assessing the risk of testing positive among individuals with at least one administered test, individuals of lower income groups had a higher risk of testing positive. Furthermore, lower income groups had a higher risk of subsequent healthcare use following a (suspicion of) COVID-19 infection, in terms of hospital and ICU admission. We did not observe any differences in COVID-19-related GP consultations between low and high income groups. Lastly, we found that individuals of lower income groups were at higher risk of dying with COVID-19, which could partly be explained by a higher risk of COVID-19 hospitalization or ICU admission and partly by a higher risk of death when being hospitalized. Overall, our findings seems to suggest that socioeconomic disparities became more pronounced at each step of the care pathway.

The contradicting direction of the association between SES and positive COVID-19 tests among the full Dutch population and individuals with at least one administered test may imply that COVID-19 diagnosis may have been under reported among lower income groups. Testing behavior may have concealed differences in infection rates between socioeconomic groups. Hence, the number of positive tests in this study cannot directly be translated to infection rates. Lower testing rates may not been necessarily due to reluctance to test [57], but possibly because of perceived barriers such as accessibility of the test location, digital barriers in making appointments and recognizing health symptoms [14, 58, 59]. On the other hand, it could also be that individuals belonging to higher income groups tested more often than necessary given their symptoms because testing was offered free of charge in the Netherlands during the study period. Because we had no data available on COVID-19-related symptoms during the study period, we cannot conclude whether the differences in testing behavior are due to differences in the occurrence of symptoms, relative underconsumption by individuals belonging to lower income groups, relative overconsumption of individuals belonging to higher income groups, applied testing policies or a combination of these.

Our findings among approximately 200,000 patients from general practitioners in The Hague greater area indicated that individuals belonging to middle income groups were more likely to consult the GP for COVID-19-related complaints than individuals belonging to higher income groups. The risk of consulting a GP for COVID-19-related complaints did not statistically significantly differ between the lowest and highest income groups. To our knowledge, no research has evaluated socioeconomic differences in GP- visits for COVID-19 or COVID-19-related symptoms. In the primary care setting, literature focused mainly on disrupted care, e.g., for chronic diseases or cancer diagnosis [7], and the emergence and uptake of teleconsulting [8]. In addition, the pattern of middle incomes being more likely to visit a GP, but higher incomes not differing from lower incomes, is inconsistent with previous findings of pre-pandemic GP visits in the Netherlands [17]. Due to the inherent limitations of the study design and use of registration data, we could not determine whether these outcomes stemmed from a different consumption pattern in Covid than for other conditions, underconsumption by low income groups during the COVID-19 pandemic, or overconsumption by higher income groups. Alternatively, income differences in COVID-19 incidence and severity may be partly driven by having a non-Dutch country of origin [60]. The lowest income groups in the Hague greater area may have a greater share of individuals with a non-Dutch country of origin, which may affect comparability between findings in the Dutch population and the Hague greater area.

Furthermore, among the full Dutch population, we found that individuals belonging to lower income groups were at higher risk for being admitted to the hospital and ICU compared to individuals belonging to higher income groups in the general population. It could be reasoned that a more severe disease course among lower socioeconomic groups could be driven by poorer pre-pandemic health status and more comorbidities, which is widely shown for non-COVID-19 healthcare use and mortality [17, 18]. Our results indicated, however, that the elixhauser comorbidity index could not explain income differences in COVID-19 hospital and ICU admission. Possibly, the elixhauser comorbidity index, based on prior hospital visits, inadequately proxies health status or inadequately captures income differences in health status. The finding that individuals belonging to lower income groups were at greater risk of serious COVID-19 illness, even after controlling for a poorer pre-pandemic health status, is consistent with findings from Spain [61] and Switzerland [28]. However, other studies showed that the association between income and COVID-19 hospitalization may be dependent on study period and included confounders. In Sweden, income was not associated with COVID-19 hospitalization in the first six months of the pandemic [30] but in another Swedish study negatively associated in the period March 2020 to March 2022 [62]. In Finland and England, lower income groups had a higher risk of hospitalization for COVID-19, but this difference did attenuate when controlling for risk factors, among others comorbidities [60, 63]. In addition, other unidentified factors might have contributed to the higher risk of hospital and ICU admission among lower income groups. For example, differences in lifestyle factors by SES, such as BMI, smoking and physical activity [64, 65] may have played a significant role in the disease course [66], which was not captured adequately by pre-pandemic healthcare use or any other covariate used.

Lastly, we found that individuals belonging to lower income groups were at higher risk for dying with COVID-19, which is consisted with findings from other European countries, such as Sweden [67] and Switzerland [28]. They are also comparable to findings from the first wave (March–June, 2020) in the Netherlands, as Stoeldraijer et al. [26] indicated that the lowest income quintile had a three times higher risk of COVID-19 mortality compared to the highest income quintile. Wouterse et al. [27] showed that income differences in all-cause mortality was larger than predicted during the pandemic in the Netherland. Although income differences in non-COVID mortality were smaller than predicted, the income differences COVID-19 mortality drove the larger differences in all-cause mortality. Therefore, they argue that the COVID-19 pandemic has enlarged already existing income differences in mortality in the Netherlands. Similar to hospital and ICU admission, higher mortality rates may result from a higher infection rate and/or a more severe disease course. However, no support was found for these mechanisms, possibly due to the study’s limitations. The question then still remains: what could explain the higher COVID-19 mortality rates among lower socio-economic groups? In our study, we found that income difference in COVID-19-related mortality risk decreased when taking into account differences in hospital and ICU admissions for COVID-19, indicating that the differences in hospitalization could partly, but not entirely, explain why individuals belonging to lower income groups were more likely to die with COVID-19. Among hospitalized patients, individuals belonging to lower income groups retained a higher COVID-19 mortality risk. Differences in testing behavior and test results did not contributed to the observed higher risk of dying with COVID-19 among lower income groups.

### Strengths and limitations

The strength of this study lies in the assessment of the entire care pathway, from testing to mortality, while other studies only looked at testing, hospitalization and mortality as single outcome measures. We were thus able to provide a comprehensive overview of the emergence of socioeconomic differences along the care pathway. This study is, to our knowledge, the first to gain insights into socioeconomic differences in COVID-19-related GP consultations. In addition, this study was based on population-wide individual level registration data, which eliminated any selection or recall biases.

Our research was also subjected to some limitations. The sample from The Hague greater area, used to analyze the GP consultations, covered approximately 2% of the Dutch population. While the subsample was representative on outcome measures and SES indicators, a higher degree of urbanity could skew the results towards higher SES differences in GP consultations. To reduce bias, we correct for degree of urbanity, household size, and country of origin in all regression analyses. Because of the small probability of ICU admission and mortality, the sample was too small to perform advanced analyses and incorporate GP consultations into the model explaining socioeconomic differences in COVID-19 mortality. Another limiting factor is that the COVID-19 test dataset did not contain administered and positive tests at commercial test centers and tests performed by employers and healthcare facilities. Some employers organized test facilities for their employees, which was for example common for healthcare workers. Commercial test centers emerged due to capacity constraints at the test centers from the municipal health services, and charged costs for administering a COVID-19 test. There is no insight into the total number of tests conducted by parties other than the Municipal Health Services. Journalism research claimed that at peak about one-third of the tests were conducted by commercial parties [68]. Due to the costs of testing at commercial test centers, it is to be expected that higher income individuals were more likely to test at commercial test centers, which would underestimate differences in COVID-19 testing based on SES. Furthermore, due to the use of registration data, it was not possible to identify whether COVID-19 was the primary cause for hospitalization or mortality, or whether it was a comorbidity to other health complaints. In the case of pre-existing life-threatening illnesses or old age combined with a COVID-19 infection, the registered cause of death depends on the physician's assessment [69]. Multi-cause mortality may cause errors in registration of actual COVID-19 mortality, but this bias is likely equally distributed among SES-groups. Lastly, inclusion of outcome indicators reflecting the entire healthcare pathway limited us in longitudinal data availability, with total overlap in data availability only covering eight months. This period includes the low-infectivity period in summer 2020 and ends at the height of the winter resurgence in 2021. While that provides a realistic representation of the pandemic, additional longitudinal data are needed to assess potential changes in SES-mediated outcome differences over the course of the pandemic.

### Conclusions, policy implications and further research

The COVID-19 pandemic had major consequences for the entire Dutch population, but individuals with lower pre-pandemic socioeconomic status experienced significantly higher risks of COVID-19 hospitalization and mortality. The socioeconomic differences became more pronounced at each step of the care pathway, culminating to a large gap in mortality rates. These findings indicated that existing socioeconomic differences were reflected in an unequal distribution of the burden of a new communicable disease. As SES-differences in testing behavior and test results did not contribute to SES-differences in mortality, it may be especially important to improve the starting position of lower SES-groups. Policy aimed at social security and well-being may, therefore, also contribute to reduced vulnerability of lower SES-groups in a crisis situation such as the COVID-19 pandemic, the utilization of health potential and prevention or worsening of illness. Furthermore, infection prevention may be particularly valuable in lower SES-groups as they were at higher risk to experience a more severe disease course.

The descriptive analyses in this research did not explain why individuals belonging to lower income groups had a higher risk of COVID-19 hospitalization and mortality. Even though previous studies have often shown that individuals belonging to lower income groups generally used more healthcare and had a higher mortality risk because they were in poorer health, socioeconomic differences in COVID-19 hospitalization and mortality did not decrease in our study when accounting for a proxy of pre-pandemic health status. Therefore, more research is needed specifically on the contribution of health status to SES-differences in COVID-19 hospitalization and mortality and to what extent health promotion can contribute to reducing these differences. Future research into other causal mechanisms underlying these trends, such as lifestyle, behavioral, cultural, and contextual factors could inform policy makers on the extent that socioeconomic differences could have been prevented in each step of the care pathway. The dynamics in individual care pathways, e.g., using individual regression lines, could also provide additional insight. This way important lessons can be retrieved about mechanism driving the unequal distribution of the burden of a new communicable disease among socioeconomic groups. These insights will be beneficial to take equity into account in pandemic preparedness plans currently being developed.

## Supplementary Information

Below is the link to the electronic supplementary material.

Supplementary file 1 (DOCX 36 KB)

Supplementary file 2 (DOCX 37 KB)

Supplementary file 3 (DOCX 379 KB)

## Data Availability

Results are based on non-public microdata from Statistics Netherlands. Under certain conditions, these microdata are accessible for statistical and scientific research.
