# Supplementary material for: From test to rest: evaluating socioeconomic differences along the COVID-19 care pathway in the Netherlands
Source: Eur J Health Econ. 2024 Mar 18;25(9):1581–94. doi: 10.1007/s10198-024-01680-4 (PMC11512841; doi:10.1007/s10198-024-01680-4)
Supplement: Supplementary file 1 — (DOCX 36 KB) [file 10198_2024_1680_MOESM1_ESM.docx]

European Journal of Health Economics

From test to rest: evaluating socioeconomic differences along the COVID-19 care pathway in the Netherlands

Iris Meulman^1,2^, Ellen Uiters^3^, Mariëlle Cloin^1^, Jeroen Struijs^2,4^ , Johan Polder^1,2^, Niek Stadhouders^5^

^1^ Tranzo, Tilburg School of Social and Behavioral Sciences, Tilburg University, Tilburg, The Netherlands

^2^ Center for Public Health, Health Services & Society, National Institute for Public Health and the Environment, Bilthoven, The Netherlands

^3^ Center for Prevention, Lifestyle and Health, National Institute for Public Health and the Environment, Bilthoven, The Netherlands

^4^ Department of Public Health and Primary Care, Leiden University Medical Center – Health Campus The Hague, The Hague, The Netherlands

^5^ Scientific Center for Quality of Healthcare, Radboud University Medical Center, Nijmegen, the Netherlands.

## Address for correspondence

Iris Meulman; Center for Health and Society, National Institute for Public Health and the Environment, Bilthoven, The Netherlands; P.O. Box 1, 3720 BA; Bilthoven, The Netherlands; Telephone: +31 30 274 4398; Email: [iris.meulman@rivm.nl](mailto:iris.meulman@rivm.nl)

# Appendix 1 – Identification strategy GP consultations

For the covid-19 related GP consultations, we used individual routine registration data of claimed contacts recorded in the electronic health records (EHR) between June 1^st^ 2020 and December 31^st^ 2020 in general practices participating in the Extramural LUMC Academic Network (ELAN). The ELAN database consists of approximately 100 participating general practices, located in the greater The Hague region and the city of Leiden, located in the province of South Holland, the Netherlands.

## Patient population delimitation

Because practices process their patient enrolment differently, various identification strategies had to be applied.

1. **Identification based on patient enrollment**In the Netherlands, general practices declared a quarterly enrolment fee per patient with the health insurer. To ensure registration quality, enrolled patients were included if at least 80% of the declared procedures at their general practice were performed for the registered patient. 99 practices with in total 328,804 patients were included via this strategy.
2. **Identification based on declared procedures**Some general practices outsource the processing of EHR. In some of these cases, the quarterly enrolment fees were not included in the dataset, while procedures were declared and registered. We assumed that patient enrolment was not processed properly in a general practice if less than 20% of the patients with at least one declared procedure in 2020 had a declared enrolment. In this case, we included all patients with at least one declared procedure as enrolled patients. Consequently, we overestimated the enrolled population because transients were also included in the population. However, the bias resulting from this overestimation was smaller than completely excluding these practices. 17,124 patients were included via this strategy, distributed over less than 10 practices.
3. **Exclusion based on the number of enrolled patients**Practices were excluded if less than 500 patients were enrolled according to the above identification strategies. This cut-off corresponded with the guidelines of another Dutch processor of GP registration data [69]. Less than 10 practices, with in total 238 patients, were excluded because of their patient population size.

## Covid-19 related GP consultations

Because an International Classification of Primary Care (ICPC) code for covid-19 only became available in November 2020, the identification of covid-19 related GP consultation was based on a combination of free text analysis and ICPC code criteria. GP consultations were marked as covid-19 consultations if the free text included ‘covid’, ‘corona’ or ‘sars’ and were not related to the absence of the condition, coronary diseases, vaccination, post-COVID-19 symptoms, information provision, preventive consultations, family illnesses, mortality registrations or transients. In addition, consultations were excluded if they included ICPC codes: A20, A27, A49, A97, A97.01, R27, R83.04, all P-ICPC codes and all Z-ICPC codes.
