# Supplementary material for: From test to rest: evaluating socioeconomic differences along the COVID-19 care pathway in the Netherlands
Source: Eur J Health Econ. 2024 Mar 18;25(9):1581–94. doi: 10.1007/s10198-024-01680-4 (PMC11512841; doi:10.1007/s10198-024-01680-4)
Supplement: Supplementary file 2 — (DOCX 37 KB) [file 10198_2024_1680_MOESM2_ESM.docx]

European Journal of Health Economics

From test to rest: evaluating socioeconomic differences along the COVID-19 care pathway in the Netherlands

Iris Meulman^1,2^, Ellen Uiters^3^, Mariëlle Cloin^1^, Jeroen Struijs^2,4^ , Johan Polder^1,2^, Niek Stadhouders^5^

^1^ Tranzo, Tilburg School of Social and Behavioral Sciences, Tilburg University, Tilburg, The Netherlands

^2^ Center for Public Health, Health Services & Society, National Institute for Public Health and the Environment, Bilthoven, The Netherlands

^3^ Center for Prevention, Lifestyle and Health, National Institute for Public Health and the Environment, Bilthoven, The Netherlands

^4^ Department of Public Health and Primary Care, Leiden University Medical Center – Health Campus The Hague, The Hague, The Netherlands

^5^ Scientific Center for Quality of Healthcare, Radboud University Medical Center, Nijmegen, the Netherlands.

## Address for correspondence

Iris Meulman; Center for Health and Society, National Institute for Public Health and the Environment, Bilthoven, The Netherlands; P.O. Box 1, 3720 BA; Bilthoven, The Netherlands; Telephone: +31 30 274 4398; Email: [iris.meulman@rivm.nl](mailto:iris.meulman@rivm.nl)

# Appendix 2 – Elixhauser ICD-10 codes

| **Comorbidity** | **ICD-10 codes** |
| --- | --- |
| Aids-HIV | B20 to B22, B24 |
| Alcohol abuse | F010, E052, G621, I426, K292, K700, K703, K709, T051, Z502, Z714, Z721 |
| Blood loss anemia | D500 |
| Cardiac arrhythmias | I441 to I443, I456, I459, I470 to I499, R000, R001, R009, T821, Z450, Z950 |
| Chronic pulmonary disease | I278, I279, J40 to J47, J60 to J67, J684, J701, J703 |
| Coagulopathy | D65 to D68, D691, D693 to D696 |
| Congestive heart failure | I099, I110, I130, I132, I125, I420, I425 to I429, I43, I500 to I509, P290 |
| Deficiency anemia | D508, D509, D51 to D53 |
| Depression | F204, F313 to F315, F32, F33, F341, F412, F432 |
| Diabetes complicated | E102 to E108, E112 to E118, E122 to E128, E132 to E138, E142 to E148 |
| Diabetes uncomplicated | E100, E101, E109, E110, E111, E119, E120, E121, E129, E130, E131, E139, E140, E141, E149 |
| Drug abuse | F11 to F16, F18, Z19, Z715, Z722 |
| Fluid and electrolyte disorders | E222, E86, E87 |
| Hypertension complicated | I110 to I119, I130 to I139, I150 to I159 |
| Hypertension uncomplicated | I10 |
| Hypothyroidism | E00 to E03, E890 |
| Liver disease | B18, I85, I864, I982, K70, K711, K713 to K715, K717, K760, K72 to K74, K762, Z944 |
| Lymphoma | C81 to C85, C88, C96, C900, C902 |
| Metastatic cancer | C77 to C80 |
| Obesity | E66 |
| Other neurological disorders | G100 to G139, G200 to G229, G254, G255, G312, G318, G319, G320 to G329, G350 to G379, G400 to G419, G931, G934, R470, R56 |
| Paralysis | G041, G114, G801, G802, G81, G82, G830 to G834, G839 |
| Peptic ulcer disease | K257, K259, K267, K269, K277, K279, K287, K289 |
| Peripheral vascular disorder | I700 to I709, I710 to I719, I731, I738, I739, I771, I790, I792, K551, K558, K559, Z958, Z959 |
| Psychoses | F20, F22 to F25, F28, F29, F302, F312, F315 |
| Pulmonary circulation disorders | I260 to I269, I270 to I279, I280, I288, I289 |
| Renal failure | I120, I131, N18, N19, N250, Z490 to Z492, Z940, Z992 |
| Solid tumor without metastasis | C00 to C26, C30 to C34, C37 to C41, C43, C45 to C58, C60 to C76, C97 |
| Rheumatoid arthritis collagen casula diseases | L940, L941, L943, M05, M06, M08, M120, M123, M30, M310 to M313, M32 to M35, M45, M461, M468, M469 |
| Vulvular disease | A520, I050 to I089, I091, I098, I340 to I399, Q230 to Q232, Q233, Z952, Z954 |
| Weight loss | E40 to E46, R634, R64 |
