# Supplementary material for: From test to rest: evaluating socioeconomic differences along the COVID-19 care pathway in the Netherlands
Source: Eur J Health Econ. 2024 Mar 18;25(9):1581–94. doi: 10.1007/s10198-024-01680-4 (PMC11512841; doi:10.1007/s10198-024-01680-4)
Supplement: Supplementary file 3 — (DOCX 379 KB) [file 10198_2024_1680_MOESM3_ESM.docx]

European Journal of Health Economics

From test to rest: evaluating socioeconomic differences along the COVID-19 care pathway in the Netherlands

Iris Meulman^1,2^, Ellen Uiters^3^, Mariëlle Cloin^1^, Jeroen Struijs^2,4^ , Johan Polder^1,2^, Niek Stadhouders^5^

^1^ Tranzo, Tilburg School of Social and Behavioral Sciences, Tilburg University, Tilburg, The Netherlands

^2^ Center for Public Health, Health Services & Society, National Institute for Public Health and the Environment, Bilthoven, The Netherlands

^3^ Center for Prevention, Lifestyle and Health, National Institute for Public Health and the Environment, Bilthoven, The Netherlands

^4^ Department of Public Health and Primary Care, Leiden University Medical Center – Health Campus The Hague, The Hague, The Netherlands

^5^ Scientific Center for Quality of Healthcare, Radboud University Medical Center, Nijmegen, the Netherlands.

## Address for correspondence

Iris Meulman; Center for Health and Society, National Institute for Public Health and the Environment, Bilthoven, The Netherlands; P.O. Box 1, 3720 BA; Bilthoven, The Netherlands; Telephone: +31 30 274 4398; Email: [iris.meulman@rivm.nl](mailto:iris.meulman@rivm.nl)

# Appendix 3 – Tables and Sensitivity analyses

## Administered COVID-19 test – Income, financial wealth, education and imputed education

Table A 1 Administered COVID-19 tests by income decile

|  | Model 1 | | | Model 2 | | | Model 3 | | |
| --- | --- | --- | --- | --- | --- | --- | --- | --- | --- |
|  | RR | 95% CI | | RR | 95% CI | | RR | 95% CI | |
| **Income decile** |  |  |  |  |  |  |  |  |  |
| 1 | 0.57 | 0.57 | 0.57 | 0.56 | 0.56 | 0.57 | 0.61 | 0.60 | 0.61 |
| 2 | 0.75 | 0.75 | 0.75 | 0.74 | 0.74 | 0.75 | 0.78 | 0.78 | 0.78 |
| 3 | 0.86 | 0.85 | 0.86 | 0.85 | 0.85 | 0.86 | 0.88 | 0.87 | 0.88 |
| 4 | 0.91 | 0.91 | 0.92 | 0.91 | 0.91 | 0.91 | 0.93 | 0.92 | 0.93 |
| 5 | 0.96 | 0.95 | 0.96 | 0.95 | 0.95 | 0.96 | 0.96 | 0.96 | 0.97 |
| 6 | 0.99 | 0.98 | 0.99 | 0.98 | 0.98 | 0.99 | 0.99 | 0.99 | 0.99 |
| 7 | 1.01 | 1.01 | 1.02 | 1.01 | 1.01 | 1.01 | 1.01 | 1.01 | 1.02 |
| 8 | 1.03 | 1.03 | 1.04 | 1.03 | 1.03 | 1.03 | 1.03 | 1.03 | 1.04 |
| 9 | 1.04 | 1.03 | 1.04 | 1.04 | 1.03 | 1.04 | 1.04 | 1.03 | 1.04 |
| 10 | 1 | 1 | 1 | 1 | 1 | 1 | 1 | 1 | 1 |
| **Sex** |  |  |  |  |  |  |  |  |  |
| Male | 1 | 1 | 1 | 1 | 1 | 1 | 1 | 1 | 1 |
| Female | 1.25 | 1.24 | 1.25 | 1.23 | 1.23 | 1.23 | 1.22 | 1.22 | 1.22 |
| **Age** |  |  |  |  |  |  |  |  |  |
| 25-44 | 1 | 1 | 1 | 1 | 1 | 1 | 1 | 1 | 1 |
| 45-64 | 0.68 | 0.68 | 0.68 | 0.66 | 0.66 | 0.66 | 0.67 | 0.67 | 0.67 |
| 65-79 | 0.40 | 0.40 | 0.40 | 0.37 | 0.37 | 0.37 | 0.40 | 0.39 | 0.40 |
| **Comorbidities** |  |  |  |  |  |  |  |  |  |
| No |  |  |  | 1 | 1 | 1 | 1 | 1 | 1 |
| Yes |  |  |  | 1.21 | 1.20 | 1.21 | 1.20 | 1.19 | 1.20 |
| **Urbanity** |  |  |  |  |  |  |  |  |  |
| 1 |  |  |  |  |  |  | 1 | 1 | 1 |
| 2 |  |  |  |  |  |  | 1.10 | 1.09 | 1.10 |
| 3 |  |  |  |  |  |  | 1.14 | 1.14 | 1.15 |
| 4 |  |  |  |  |  |  | 1.18 | 1.17 | 1.18 |
| 5 |  |  |  |  |  |  | 1.25 | 1.24 | 1.25 |
| 6 |  |  |  |  |  |  | 1.36 | 1.36 | 1.37 |
| **Household size** |  |  |  |  |  |  |  |  |  |
| 1 |  |  |  |  |  |  | 1 | 1 | 1 |
| 2 |  |  |  |  |  |  | 1.04 | 1.03 | 1.04 |
| 3-4 |  |  |  |  |  |  | 1.19 | 1.18 | 1.19 |
| >4 |  |  |  |  |  |  | 1.16 | 1.16 | 1.17 |
| **Country of origin** |  |  |  |  |  |  |  |  |  |
| Dutch |  |  |  |  |  |  | 1 | 1 | 1 |
| Europe |  |  |  |  |  |  | 0.77 | 0.76 | 0.77 |
| Other |  |  |  |  |  |  | 0.82 | 0.82 | 0.83 |

Table A 1 Administered COVID-19 tests by lagged income decile

|  | Model 1 | | | Model 2 | | | Model 3 | | |
| --- | --- | --- | --- | --- | --- | --- | --- | --- | --- |
|  | RR | 95% CI | | RR | 95% CI | | RR | 95% CI | |
| **Lagged income decile** |  |  |  |  |  |  |  |  |  |
| 1 | 0.62 | 0.62 | 0.62 | 0.61 | 0.61 | 0.62 | 0.66 | 0.65 | 0.66 |
| 2 | 0.77 | 0.76 | 0.77 | 0.76 | 0.76 | 0.77 | 0.80 | 0.80 | 0.81 |
| 3 | 0.87 | 0.87 | 0.87 | 0.87 | 0.86 | 0.87 | 0.89 | 0.89 | 0.89 |
| 4 | 0.92 | 0.92 | 0.93 | 0.92 | 0.92 | 0.92 | 0.94 | 0.93 | 0.94 |
| 5 | 0.96 | 0.95 | 0.96 | 0.95 | 0.95 | 0.96 | 0.97 | 0.96 | 0.97 |
| 6 | 0.99 | 0.99 | 0.99 | 0.99 | 0.98 | 0.99 | 1.00 | 0.99 | 1.00 |
| 7 | 1.01 | 1.01 | 1.02 | 1.01 | 1.01 | 1.01 | 1.02 | 1.01 | 1.02 |
| 8 | 1.03 | 1.03 | 1.03 | 1.03 | 1.02 | 1.03 | 1.03 | 1.03 | 1.04 |
| 9 | 1.04 | 1.03 | 1.04 | 1.03 | 1.03 | 1.04 | 1.04 | 1.03 | 1.04 |
| 10 | 1 | 1 | 1 | 1 | 1 | 1 | 1 | 1 | 1 |
| **Sex** |  |  |  |  |  |  |  |  |  |
| Male | 1 | 1 | 1 | 1 | 1 | 1 | 1 | 1 | 1 |
| Female | 1.25 | 1.25 | 1.25 | 1.23 | 1.23 | 1.23 | 1.22 | 1.22 | 1.22 |
| **Age** |  |  |  |  |  |  |  |  |  |
| 25-44 | 1 | 1 | 1 | 1 | 1 | 1 | 1 | 1 | 1 |
| 45-64 | 0.68 | 0.68 | 0.68 | 0.66 | 0.66 | 0.66 | 0.66 | 0.66 | 0.67 |
| 65-79 | 0.40 | 0.40 | 0.40 | 0.37 | 0.37 | 0.37 | 0.40 | 0.39 | 0.40 |
| **Comorbidities** |  |  |  |  |  |  |  |  |  |
| No |  |  |  | 1 | 1 | 1 | 1 | 1 | 1 |
| Yes |  |  |  | 1.21 | 1.20 | 1.21 | 1.20 | 1.19 | 1.20 |
| **Urbanity** |  |  |  |  |  |  |  |  |  |
| 1 |  |  |  |  |  |  | 1 | 1 | 1 |
| 2 |  |  |  |  |  |  | 1.10 | 1.09 | 1.10 |
| 3 |  |  |  |  |  |  | 1.14 | 1.14 | 1.15 |
| 4 |  |  |  |  |  |  | 1.18 | 1.17 | 1.18 |
| 5 |  |  |  |  |  |  | 1.25 | 1.24 | 1.25 |
| 6 |  |  |  |  |  |  | 1.37 | 1.36 | 1.37 |
| **Household size** |  |  |  |  |  |  |  |  |  |
| 1 |  |  |  |  |  |  | 1 | 1 | 1 |
| 2 |  |  |  |  |  |  | 1.06 | 1.06 | 1.06 |
| 3-4 |  |  |  |  |  |  | 1.22 | 1.21 | 1.22 |
| >4 |  |  |  |  |  |  | 1.20 | 1.19 | 1.20 |
| **Country of origin** |  |  |  |  |  |  |  |  |  |
| Dutch |  |  |  |  |  |  | 1 | 1 | 1 |
| Europe |  |  |  |  |  |  | 0.76 | 0.76 | 0.76 |
| Other |  |  |  |  |  |  | 0.82 | 0.81 | 0.82 |

Table A 2 Administered COVID-19 tests by financial wealth decile

|  | Model 1 | | | Model 2 | | | Model 3 | | |
| --- | --- | --- | --- | --- | --- | --- | --- | --- | --- |
|  | RR | 95% CI | | RR | 95% CI | | RR | 95% CI | |
| **Financial wealth decile** |  |  |  |  |  |  |  |  |  |
| 1 | 0.99 | 0.99 | 1.00 | 0.98 | 0.98 | 0.98 | 1.04 | 1.03 | 1.04 |
| 2 | 0.84 | 0.83 | 0.84 | 0.82 | 0.82 | 0.83 | 0.91 | 0.91 | 0.92 |
| 3 | 0.85 | 0.84 | 0.85 | 0.84 | 0.83 | 0.84 | 0.92 | 0.91 | 0.92 |
| 4 | 0.96 | 0.96 | 0.96 | 0.96 | 0.95 | 0.96 | 1.01 | 1.01 | 1.02 |
| 5 | 1.04 | 1.03 | 1.04 | 1.03 | 1.03 | 1.04 | 1.07 | 1.07 | 1.08 |
| 6 | 1.07 | 1.07 | 1.07 | 1.07 | 1.06 | 1.07 | 1.09 | 1.09 | 1.10 |
| 7 | 1.09 | 1.08 | 1.09 | 1.08 | 1.08 | 1.09 | 1.10 | 1.10 | 1.11 |
| 8 | 1.09 | 1.09 | 1.10 | 1.09 | 1.08 | 1.09 | 1.10 | 1.10 | 1.10 |
| 9 | 1.07 | 1.06 | 1.07 | 1.07 | 1.06 | 1.07 | 1.07 | 1.07 | 1.08 |
| 10 | 1 | 1 | 1 | 1 | 1 | 1 | 1 | 1 | 1 |
| **Sex** |  |  |  |  |  |  |  |  |  |
| Male | 1 | 1 | 1 | 1 | 1 | 1 | 1 | 1 | 1 |
| Female | 1.23 | 1.23 | 1.23 | 1.21 | 1.21 | 1.22 | 1.21 | 1.21 | 1.21 |
| **Age** |  |  |  |  |  |  |  |  |  |
| 25-44 | 1 | 1 | 1 | 1 | 1 | 1 | 1 | 1 | 1 |
| 45-64 | 0.68 | 0.68 | 0.68 | 0.66 | 0.66 | 0.66 | 0.66 | 0.66 | 0.67 |
| 65-79 | 0.40 | 0.40 | 0.40 | 0.37 | 0.37 | 0.37 | 0.39 | 0.39 | 0.40 |
| **Comorbidities** |  |  |  |  |  |  |  |  |  |
| No |  |  |  | 1 | 1 | 1 | 1 | 1 | 1 |
| Yes |  |  |  | 1.20 | 1.20 | 1.21 | 1.19 | 1.19 | 1.19 |
| **Urbanity** |  |  |  |  |  |  |  |  |  |
| 1 |  |  |  |  |  |  | 1 | 1 | 1 |
| 2 |  |  |  |  |  |  | 1.10 | 1.09 | 1.10 |
| 3 |  |  |  |  |  |  | 1.14 | 1.14 | 1.15 |
| 4 |  |  |  |  |  |  | 1.18 | 1.17 | 1.18 |
| 5 |  |  |  |  |  |  | 1.25 | 1.24 | 1.25 |
| 6 |  |  |  |  |  |  | 1.38 | 1.37 | 1.38 |
| **Household size** |  |  |  |  |  |  |  |  |  |
| 1 |  |  |  |  |  |  | 1 | 1 | 1 |
| 2 |  |  |  |  |  |  | 1.10 | 1.10 | 1.11 |
| 3-4 |  |  |  |  |  |  | 1.26 | 1.25 | 1.26 |
| >4 |  |  |  |  |  |  | 1.22 | 1.22 | 1.22 |
| **Country of origin** |  |  |  |  |  |  |  |  |  |
| Dutch |  |  |  |  |  |  | 1 | 1 | 1 |
| Europe |  |  |  |  |  |  | 0.75 | 0.75 | 0.75 |
| Other |  |  |  |  |  |  | 0.77 | 0.77 | 0.78 |

Table A 3 Administered COVID-19 test by education

|  | Model 1 | | | Model 2 | | | Model 3 | | |
| --- | --- | --- | --- | --- | --- | --- | --- | --- | --- |
|  | RR | 95% CI | | RR | 95% CI | | RR | 95% CI | |
| **Education** |  |  |  |  |  |  |  |  |  |
| Low | 0.61 | 0.60 | 0.61 | 0.60 | 0.60 | 0.60 | 0.63 | 0.63 | 0.63 |
| Middle | 0.80 | 0.80 | 0.80 | 0.79 | 0.79 | 0.79 | 0.81 | 0.81 | 0.81 |
| High | 1 | 1 | 1 | 1 | 1 | 1 | 1 | 1 | 1 |
| **Sex** |  |  |  |  |  |  |  |  |  |
| Male | 1 | 1 | 1 | 1 | 1 | 1 | 1 | 1 | 1 |
| Female | 1.25 | 1.24 | 1.25 | 1.23 | 1.23 | 1.23 | 1.22 | 1.22 | 1.22 |
| **Age** |  |  |  |  |  |  |  |  |  |
| 25-44 | 1 | 1 | 1 | 1 | 1 | 1 | 1 | 1 | 1 |
| 45-64 | 0.72 | 0.72 | 0.72 | 0.70 | 0.70 | 0.70 | 0.71 | 0.71 | 0.71 |
| 65-79 | 0.48 | 0.48 | 0.49 | 0.46 | 0.46 | 0.46 | 0.49 | 0.49 | 0.49 |
| **Comorbidities** |  |  |  |  |  |  |  |  |  |
| No |  |  |  | 1 | 1 | 1 | 1 | 1 | 1 |
| Yes |  |  |  | 1.17 | 1.17 | 1.18 | 1.17 | 1.17 | 1.17 |
| **Urbanity** |  |  |  |  |  |  |  |  |  |
| 1 |  |  |  |  |  |  | 1 | 1 | 1 |
| 2 |  |  |  |  |  |  | 1.08 | 1.07 | 1.08 |
| 3 |  |  |  |  |  |  | 1.11 | 1.11 | 1.11 |
| 4 |  |  |  |  |  |  | 1.13 | 1.13 | 1.14 |
| 5 |  |  |  |  |  |  | 1.18 | 1.18 | 1.19 |
| 6 |  |  |  |  |  |  | 1.25 | 1.24 | 1.26 |
| **Household size** |  |  |  |  |  |  |  |  |  |
| 1 |  |  |  |  |  |  | 1 | 1 | 1 |
| 2 |  |  |  |  |  |  | 1.11 | 1.10 | 1.11 |
| 3-4 |  |  |  |  |  |  | 1.27 | 1.27 | 1.27 |
| >4 |  |  |  |  |  |  | 1.23 | 1.22 | 1.23 |
| **Country of origin** |  |  |  |  |  |  |  |  |  |
| Dutch |  |  |  |  |  |  | 1 | 1 | 1 |
| Europe |  |  |  |  |  |  | 0.83 | 0.83 | 0.84 |
| Other |  |  |  |  |  |  | 0.83 | 0.83 | 0.83 |

Table A 4 Administered COVID-19 test by imputed education

|  | Model 1 | | | Model 2 | | | Model 3 | | |
| --- | --- | --- | --- | --- | --- | --- | --- | --- | --- |
|  | RR | 95% CI | | RR | 95% CI | | RR | 95% CI | |
| **Imputed education** |  |  |  |  |  |  |  |  |  |
| Low | 0.64 | 0.64 | 0.65 | 0.64 | 0.63 | 0.64 | 0.66 | 0.66 | 0.67 |
| Middle | 0.81 | 0.80 | 0.81 | 0.80 | 0.80 | 0.80 | 0.81 | 0.81 | 0.81 |
| High | 1 | 1 | 1 | 1 | 1 | 1 | 1 | 1 | 1 |
| **Sex** |  |  |  |  |  |  |  |  |  |
| Male | 1 | 1 | 1 | 1 | 1 | 1 | 1 | 1 | 1 |
| Female | 1.24 | 1.24 | 1.24 | 1.22 | 1.22 | 1.22 | 1.21 | 1.21 | 1.22 |
| **Age** |  |  |  |  |  |  |  |  |  |
| 25-44 | 1 | 1 | 1 | 1 | 1 | 1 | 1 | 1 | 1 |
| 45-64 | 0.71 | 0.71 | 0.72 | 0.69 | 0.69 | 0.69 | 0.69 | 0.69 | 0.69 |
| 65-79 | 0.44 | 0.44 | 0.45 | 0.42 | 0.41 | 0.42 | 0.44 | 0.43 | 0.44 |
| **Comorbidities** |  |  |  |  |  |  |  |  |  |
| No |  |  |  | 1 | 1 | 1 | 1 | 1 | 1 |
| Yes |  |  |  | 1.21 | 1.21 | 1.22 | 1.20 | 1.20 | 1.21 |
| **Urbanity** |  |  |  |  |  |  |  |  |  |
| 1 |  |  |  |  |  |  | 1 | 1 | 1 |
| 2 |  |  |  |  |  |  | 1.09 | 1.08 | 1.09 |
| 3 |  |  |  |  |  |  | 1.13 | 1.12 | 1.13 |
| 4 |  |  |  |  |  |  | 1.15 | 1.15 | 1.15 |
| 5 |  |  |  |  |  |  | 1.19 | 1.19 | 1.20 |
| 6 |  |  |  |  |  |  | 1.27 | 1.26 | 1.27 |
| **Household size** |  |  |  |  |  |  |  |  |  |
| 1 |  |  |  |  |  |  | 1 | 1 | 1 |
| 2 |  |  |  |  |  |  | 1.12 | 1.12 | 1.13 |
| 3-4 |  |  |  |  |  |  | 1.28 | 1.28 | 1.29 |
| >4 |  |  |  |  |  |  | 1.24 | 1.24 | 1.25 |
| **Country of origin** |  |  |  |  |  |  |  |  |  |
| Dutch |  |  |  |  |  |  | 1 | 1 | 1 |
| Europe |  |  |  |  |  |  | 0.75 | 0.75 | 0.76 |
| Other |  |  |  |  |  |  | 0.80 | 0.80 | 0.80 |

## Positive COVID-19 test – Income, financial wealth, education and imputed education

Table A 5 Postive COVID-19 tests by income decile

|  | Model 1 | | | Model 2 | | | Model 3 | | |
| --- | --- | --- | --- | --- | --- | --- | --- | --- | --- |
|  | RR | 95% CI | | RR | 95% CI | | RR | 95% CI | |
| **Income decile** |  |  |  |  |  |  |  |  |  |
| 1 | 0.80 | 0.79 | 0.81 | 0.79 | 0.78 | 0.80 | 0.77 | 0.76 | 0.78 |
| 2 | 0.94 | 0.92 | 0.95 | 0.93 | 0.92 | 0.94 | 0.94 | 0.92 | 0.95 |
| 3 | 1.04 | 1.02 | 1.05 | 1.03 | 1.02 | 1.04 | 1.04 | 1.03 | 1.06 |
| 4 | 1.09 | 1.07 | 1.10 | 1.08 | 1.07 | 1.10 | 1.10 | 1.09 | 1.12 |
| 5 | 1.13 | 1.11 | 1.14 | 1.12 | 1.11 | 1.14 | 1.14 | 1.13 | 1.16 |
| 6 | 1.15 | 1.14 | 1.17 | 1.15 | 1.13 | 1.16 | 1.17 | 1.15 | 1.18 |
| 7 | 1.16 | 1.14 | 1.17 | 1.16 | 1.14 | 1.17 | 1.17 | 1.16 | 1.19 |
| 8 | 1.16 | 1.14 | 1.17 | 1.15 | 1.14 | 1.17 | 1.17 | 1.15 | 1.18 |
| 9 | 1.10 | 1.08 | 1.11 | 1.10 | 1.08 | 1.11 | 1.11 | 1.09 | 1.12 |
| 10 | 1 | 1 | 1 | 1 | 1 | 1 | 1 | 1 | 1 |
| **Sex** |  |  |  |  |  |  |  |  |  |
| Male | 1 | 1 | 1 | 1 | 1 | 1 | 1 | 1 | 1 |
| Female | 1.08 | 1.08 | 1.09 | 1.07 | 1.07 | 1.08 | 1.06 | 1.05 | 1.06 |
| **Age** |  |  |  |  |  |  |  |  |  |
| 25-44 | 1 | 1 | 1 | 1 | 1 | 1 | 1 | 1 | 1 |
| 45-64 | 0.92 | 0.91 | 0.92 | 0.89 | 0.89 | 0.90 | 0.96 | 0.95 | 0.97 |
| 65-79 | 0.46 | 0.46 | 0.47 | 0.44 | 0.44 | 0.44 | 0.57 | 0.57 | 0.58 |
| **Comorbidities** |  |  |  |  |  |  |  |  |  |
| No |  |  |  | 1 | 1 | 1 | 1 | 1 | 1 |
| Yes |  |  |  | 1.16 | 1.15 | 1.17 | 1.16 | 1.15 | 1.17 |
| **Urbanity** |  |  |  |  |  |  |  |  |  |
| 1 |  |  |  |  |  |  | 1 | 1 | 1 |
| 2 |  |  |  |  |  |  | 1.11 | 1.10 | 1.12 |
| 3 |  |  |  |  |  |  | 1.16 | 1.15 | 1.17 |
| 4 |  |  |  |  |  |  | 1.17 | 1.16 | 1.18 |
| 5 |  |  |  |  |  |  | 1.29 | 1.28 | 1.31 |
| 6 |  |  |  |  |  |  | 1.46 | 1.44 | 1.48 |
| **Household size** |  |  |  |  |  |  |  |  |  |
| 1 |  |  |  |  |  |  | 1 | 1 | 1 |
| 2 |  |  |  |  |  |  | 1.27 | 1.26 | 1.28 |
| 3-4 |  |  |  |  |  |  | 1.65 | 1.63 | 1.66 |
| >4 |  |  |  |  |  |  | 2.16 | 2.14 | 2.19 |
| **Country of origin** |  |  |  |  |  |  |  |  |  |
| Dutch |  |  |  |  |  |  | 1 | 1 | 1 |
| Europe |  |  |  |  |  |  | 0.78 | 0.77 | 0.79 |
| Other |  |  |  |  |  |  | 1.44 | 1.43 | 1.45 |

Table A 1 Positive COVID-19 tests by lagged income decile

|  | Model 1 | | | Model 2 | | | Model 3 | | |
| --- | --- | --- | --- | --- | --- | --- | --- | --- | --- |
|  | RR | 95% CI | | RR | 95% CI | | RR | 95% CI | |
| **Lagged income decile** |  |  |  |  |  |  |  |  |  |
| 1 | 0.85 | 0.84 | 0.86 | 0.84 | 0.83 | 0.85 | 0.80 | 0.79 | 0.81 |
| 2 | 0.97 | 0.96 | 0.98 | 0.96 | 0.95 | 0.98 | 0.96 | 0.95 | 0.97 |
| 3 | 1.06 | 1.05 | 1.08 | 1.06 | 1.05 | 1.07 | 1.06 | 1.05 | 1.08 |
| 4 | 1.11 | 1.10 | 1.13 | 1.11 | 1.09 | 1.12 | 1.12 | 1.10 | 1.13 |
| 5 | 1.13 | 1.12 | 1.15 | 1.13 | 1.11 | 1.14 | 1.14 | 1.13 | 1.16 |
| 6 | 1.14 | 1.13 | 1.16 | 1.14 | 1.13 | 1.16 | 1.16 | 1.14 | 1.17 |
| 7 | 1.14 | 1.13 | 1.16 | 1.14 | 1.12 | 1.16 | 1.16 | 1.14 | 1.17 |
| 8 | 1.12 | 1.10 | 1.13 | 1.12 | 1.10 | 1.13 | 1.14 | 1.12 | 1.15 |
| 9 | 1.08 | 1.06 | 1.09 | 1.08 | 1.06 | 1.09 | 1.09 | 1.08 | 1.11 |
| 10 | 1 | 1 | 1 | 1 | 1 | 1 | 1 | 1 | 1 |
| **Sex** |  |  |  |  |  |  |  |  |  |
| Male | 1 | 1 | 1 | 1 | 1 | 1 | 1 | 1 | 1 |
| Female | 1.08 | 1.08 | 1.09 | 1.07 | 1.07 | 1.08 | 1.06 | 1.05 | 1.06 |
| **Age** |  |  |  |  |  |  |  |  |  |
| 25-44 | 1 | 1 | 1 | 1 | 1 | 1 | 1 | 1 | 1 |
| 45-64 | 0.91 | 0.91 | 0.92 | 0.89 | 0.88 | 0.90 | 0.96 | 0.95 | 0.97 |
| 65-79 | 0.47 | 0.46 | 0.47 | 0.44 | 0.44 | 0.45 | 0.58 | 0.57 | 0.58 |
| **Comorbidities** |  |  |  |  |  |  |  |  |  |
| No |  |  |  | 1 | 1 | 1 | 1 | 1 | 1 |
| Yes |  |  |  | 1.16 | 1.15 | 1.17 | 1.16 | 1.15 | 1.17 |
| **Urbanity** |  |  |  |  |  |  |  |  |  |
| 1 |  |  |  |  |  |  | 1 | 1 | 1 |
| 2 |  |  |  |  |  |  | 1.11 | 1.10 | 1.12 |
| 3 |  |  |  |  |  |  | 1.16 | 1.15 | 1.17 |
| 4 |  |  |  |  |  |  | 1.17 | 1.16 | 1.18 |
| 5 |  |  |  |  |  |  | 1.29 | 1.28 | 1.31 |
| 6 |  |  |  |  |  |  | 1.46 | 1.44 | 1.48 |
| **Household size** |  |  |  |  |  |  |  |  |  |
| 1 |  |  |  |  |  |  | 1 | 1 | 1 |
| 2 |  |  |  |  |  |  | 1.29 | 1.28 | 1.30 |
| 3-4 |  |  |  |  |  |  | 1.68 | 1.66 | 1.69 |
| >4 |  |  |  |  |  |  | 2.21 | 2.18 | 2.23 |
| **Country of origin** |  |  |  |  |  |  |  |  |  |
| Dutch |  |  |  |  |  |  | 1 | 1 | 1 |
| Europe |  |  |  |  |  |  | 0.78 | 0.77 | 0.79 |
| Other |  |  |  |  |  |  | 1.43 | 1.42 | 1.44 |

Table A 6 Positive COVID-19 tests by financial wealth decile

|  | Model 1 | | | Model 2 | | | Model 3 | | |
| --- | --- | --- | --- | --- | --- | --- | --- | --- | --- |
|  | RR | 95% CI | | RR | 95% CI | | RR | 95% CI | |
| **Financial wealth decile** |  |  |  |  |  |  |  |  |  |
| 1 | 1.11 | 1.09 | 1.12 | 1.10 | 1.08 | 1.11 | 1.08 | 1.06 | 1.09 |
| 2 | 0.95 | 0.94 | 0.97 | 0.94 | 0.93 | 0.96 | 1.00 | 0.99 | 1.02 |
| 3 | 0.97 | 0.96 | 0.99 | 0.97 | 0.95 | 0.98 | 1.03 | 1.02 | 1.05 |
| 4 | 1.04 | 1.03 | 1.06 | 1.04 | 1.02 | 1.05 | 1.11 | 1.09 | 1.12 |
| 5 | 1.08 | 1.07 | 1.09 | 1.07 | 1.06 | 1.09 | 1.14 | 1.13 | 1.16 |
| 6 | 1.12 | 1.11 | 1.14 | 1.12 | 1.10 | 1.13 | 1.18 | 1.17 | 1.20 |
| 7 | 1.13 | 1.11 | 1.14 | 1.13 | 1.11 | 1.14 | 1.18 | 1.17 | 1.20 |
| 8 | 1.11 | 1.10 | 1.12 | 1.11 | 1.09 | 1.12 | 1.15 | 1.14 | 1.17 |
| 9 | 1.06 | 1.04 | 1.07 | 1.06 | 1.04 | 1.07 | 1.09 | 1.07 | 1.10 |
| 10 | 1 | 1 | 1 | 1 | 1 | 1 | 1 | 1 | 1 |
| **Sex** |  |  |  |  |  |  |  |  |  |
| Male | 1 | 1 | 1 | 1 | 1 | 1 | 1 | 1 | 1 |
| Female | 1.08 | 1.07 | 1.08 | 1.07 | 1.06 | 1.07 | 1.05 | 1.04 | 1.06 |
| **Age** |  |  |  |  |  |  |  |  |  |
| 25-44 | 1 | 1 | 1 | 1 | 1 | 1 | 1 | 1 | 1 |
| 45-64 | 0.92 | 0.91 | 0.92 | 0.89 | 0.89 | 0.90 | 0.96 | 0.95 | 0.97 |
| 65-79 | 0.46 | 0.46 | 0.47 | 0.44 | 0.44 | 0.45 | 0.57 | 0.57 | 0.58 |
| **Comorbidities** |  |  |  |  |  |  |  |  |  |
| No |  |  |  | 1 | 1 | 1 | 1 | 1 | 1 |
| Yes |  |  |  | 1.16 | 1.15 | 1.16 | 1.16 | 1.15 | 1.16 |
| **Urbanity** |  |  |  |  |  |  |  |  |  |
| 1 |  |  |  |  |  |  | 1 | 1 | 1 |
| 2 |  |  |  |  |  |  | 1.11 | 1.09 | 1.12 |
| 3 |  |  |  |  |  |  | 1.15 | 1.14 | 1.17 |
| 4 |  |  |  |  |  |  | 1.16 | 1.15 | 1.18 |
| 5 |  |  |  |  |  |  | 1.28 | 1.27 | 1.30 |
| 6 |  |  |  |  |  |  | 1.45 | 1.43 | 1.47 |
| **Household size** |  |  |  |  |  |  |  |  |  |
| 1 |  |  |  |  |  |  | 1 | 1 | 1 |
| 2 |  |  |  |  |  |  | 1.33 | 1.31 | 1.34 |
| 3-4 |  |  |  |  |  |  | 1.73 | 1.72 | 1.75 |
| >4 |  |  |  |  |  |  | 2.26 | 2.23 | 2.28 |
| **Country of origin** |  |  |  |  |  |  |  |  |  |
| Dutch |  |  |  |  |  |  | 1 | 1 | 1 |
| Europe |  |  |  |  |  |  | 0.77 | 0.76 | 0.78 |
| Other |  |  |  |  |  |  | 1.37 | 1.36 | 1.38 |

Table A 7 Positive COVID-19 test by education

|  | Model 1 | | | Model 2 | | | Model 3 | | |
| --- | --- | --- | --- | --- | --- | --- | --- | --- | --- |
|  | RR | 95% CI | | RR | 95% CI | | RR | 95% CI | |
| **Education** |  |  |  |  |  |  |  |  |  |
| Low | 0.94 | 0.93 | 0.94 | 0.92 | 0.91 | 0.93 | 0.87 | 0.86 | 0.88 |
| Middle | 1.00 | 0.99 | 1.01 | 0.99 | 0.98 | 1.00 | 1.00 | 0.99 | 1.01 |
| High | 1 | 1 | 1 | 1 | 1 | 1 | 1 | 1 | 1 |
| **Sex** |  |  |  |  |  |  |  |  |  |
| Male | 1 | 1 | 1 | 1 | 1 | 1 | 1 | 1 | 1 |
| Female | 1.11 | 1.10 | 1.12 | 1.10 | 1.09 | 1.10 | 1.07 | 1.07 | 1.08 |
| **Age** |  |  |  |  |  |  |  |  |  |
| 25-44 | 1 | 1 | 1 | 1 | 1 | 1 | 1 | 1 | 1 |
| 45-64 | 0.91 | 0.90 | 0.92 | 0.89 | 0.88 | 0.90 | 0.95 | 0.94 | 0.96 |
| 65-79 | 0.49 | 0.49 | 0.50 | 0.47 | 0.47 | 0.48 | 0.61 | 0.60 | 0.62 |
| **Comorbidities** |  |  |  |  |  |  |  |  |  |
| No |  |  |  | 1 | 1 | 1 | 1 | 1 | 1 |
| Yes |  |  |  | 1.14 | 1.13 | 1.15 | 1.14 | 1.13 | 1.15 |
| **Urbanity** |  |  |  |  |  |  |  |  |  |
| 1 |  |  |  |  |  |  | 1 | 1 | 1 |
| 2 |  |  |  |  |  |  | 1.11 | 1.10 | 1.13 |
| 3 |  |  |  |  |  |  | 1.16 | 1.14 | 1.17 |
| 4 |  |  |  |  |  |  | 1.17 | 1.16 | 1.19 |
| 5 |  |  |  |  |  |  | 1.29 | 1.28 | 1.31 |
| 6 |  |  |  |  |  |  | 1.47 | 1.45 | 1.50 |
| **Household size** |  |  |  |  |  |  |  |  |  |
| 1 |  |  |  |  |  |  | 1 | 1 | 1 |
| 2 |  |  |  |  |  |  | 1.33 | 1.32 | 1.35 |
| 3-4 |  |  |  |  |  |  | 1.69 | 1.67 | 1.71 |
| >4 |  |  |  |  |  |  | 2.15 | 2.12 | 2.18 |
| **Country of origin** |  |  |  |  |  |  |  |  |  |
| Dutch |  |  |  |  |  |  | 1 | 1 | 1 |
| Europe |  |  |  |  |  |  | 0.84 | 0.82 | 0.85 |
| Other |  |  |  |  |  |  | 1.47 | 1.45 | 1.48 |

Table A 8 Positive COVID-19 test by imputed education

|  | Model 1 | | | Model 2 | | | Model 3 | | |
| --- | --- | --- | --- | --- | --- | --- | --- | --- | --- |
|  | RR | 95% CI | | RR | 95% CI | | RR | 95% CI | |
| **Imputed education** |  |  |  |  |  |  |  |  |  |
| Low | 0.95 | 0.94 | 0.96 | 0.94 | 0.93 | 0.95 | 0.91 | 0.90 | 0.92 |
| Middle | 1.01 | 1.00 | 1.02 | 1.00 | 0.99 | 1.01 | 1.01 | 1.00 | 1.02 |
| High | 1 | 1 | 1 | 1 | 1 | 1 | 1 | 1 | 1 |
| **Sex** |  |  |  |  |  |  |  |  |  |
| Male | 1 | 1 | 1 | 1 | 1 | 1 | 1 | 1 | 1 |
| Female | 1.08 | 1.07 | 1.09 | 1.07 | 1.06 | 1.08 | 1.05 | 1.05 | 1.06 |
| **Age** |  |  |  |  |  |  |  |  |  |
| 25-44 | 1 | 1 | 1 | 1 | 1 | 1 | 1 | 1 | 1 |
| 45-64 | 0.92 | 0.91 | 0.93 | 0.90 | 0.89 | 0.90 | 0.97 | 0.96 | 0.97 |
| 65-79 | 0.47 | 0.47 | 0.48 | 0.45 | 0.44 | 0.45 | 0.59 | 0.58 | 0.60 |
| **Comorbidities** |  |  |  |  |  |  |  |  |  |
| No |  |  |  | 1 | 1 | 1 | 1 | 1 | 1 |
| Yes |  |  |  | 1.16 | 1.15 | 1.16 | 1.16 | 1.15 | 1.16 |
| **Urbanity** |  |  |  |  |  |  |  |  |  |
| 1 |  |  |  |  |  |  | 1 | 1 | 1 |
| 2 |  |  |  |  |  |  | 1.11 | 1.10 | 1.12 |
| 3 |  |  |  |  |  |  | 1.16 | 1.14 | 1.17 |
| 4 |  |  |  |  |  |  | 1.17 | 1.15 | 1.18 |
| 5 |  |  |  |  |  |  | 1.28 | 1.26 | 1.29 |
| 6 |  |  |  |  |  |  | 1.43 | 1.41 | 1.45 |
| **Household size** |  |  |  |  |  |  |  |  |  |
| 1 |  |  |  |  |  |  | 1 | 1 | 1 |
| 2 |  |  |  |  |  |  | 1.35 | 1.33 | 1.36 |
| 3-4 |  |  |  |  |  |  | 1.76 | 1.74 | 1.78 |
| >4 |  |  |  |  |  |  | 2.28 | 2.25 | 2.30 |
| **Country of origin** |  |  |  |  |  |  |  |  |  |
| Dutch |  |  |  |  |  |  | 1 | 1 | 1 |
| Europe |  |  |  |  |  |  | 0.77 | 0.76 | 0.78 |
| Other |  |  |  |  |  |  | 1.37 | 1.36 | 1.38 |

## Positive COVID-19 test among individuals with at least one administered test – Income, financial wealth, education and imputed education

Table A 9 Positive COVID-19 tests among individuals with at least one administered test by income decile

|  | Model 1 | | | Model 2 | | | Model 3 | | |
| --- | --- | --- | --- | --- | --- | --- | --- | --- | --- |
|  | RR | 95% CI | | RR | 95% CI | | RR | 95% CI | |
| **Income decile** |  |  |  |  |  |  |  |  |  |
| 1 | 1.40 | 1.38 | 1.42 | 1.41 | 1.39 | 1.43 | 1.32 | 1.30 | 1.34 |
| 2 | 1.26 | 1.24 | 1.27 | 1.26 | 1.25 | 1.28 | 1.24 | 1.22 | 1.26 |
| 3 | 1.22 | 1.21 | 1.24 | 1.23 | 1.21 | 1.24 | 1.22 | 1.21 | 1.24 |
| 4 | 1.21 | 1.19 | 1.22 | 1.21 | 1.19 | 1.22 | 1.21 | 1.20 | 1.23 |
| 5 | 1.20 | 1.18 | 1.21 | 1.20 | 1.19 | 1.21 | 1.21 | 1.19 | 1.22 |
| 6 | 1.18 | 1.17 | 1.20 | 1.18 | 1.17 | 1.20 | 1.20 | 1.18 | 1.21 |
| 7 | 1.16 | 1.14 | 1.17 | 1.16 | 1.14 | 1.17 | 1.17 | 1.16 | 1.18 |
| 8 | 1.13 | 1.11 | 1.14 | 1.13 | 1.11 | 1.14 | 1.14 | 1.13 | 1.16 |
| 9 | 1.06 | 1.05 | 1.08 | 1.06 | 1.05 | 1.08 | 1.08 | 1.06 | 1.09 |
| 10 | 1 | 1 | 1 | 1 | 1 | 1 | 1 | 1 | 1 |
| **Sex** |  |  |  |  |  |  |  |  |  |
| Male | 1 | 1 | 1 | 1 | 1 | 1 | 1 | 1 | 1 |
| Female | 0.87 | 0.87 | 0.88 | 0.88 | 0.87 | 0.88 | 0.89 | 0.88 | 0.89 |
| **Age** |  |  |  |  |  |  |  |  |  |
| 25-44 | 1 | 1 | 1 | 1 | 1 | 1 | 1 | 1 | 1 |
| 45-64 | 1.33 | 1.33 | 1.34 | 1.35 | 1.34 | 1.36 | 1.41 | 1.40 | 1.42 |
| 65-79 | 1.16 | 1.15 | 1.17 | 1.19 | 1.18 | 1.20 | 1.43 | 1.42 | 1.45 |
| **Comorbidities** |  |  |  |  |  |  |  |  |  |
| No |  |  |  | 1 | 1 | 1 | 1 | 1 | 1 |
| Yes |  |  |  | 0.94 | 0.94 | 0.95 | 0.95 | 0.94 | 0.95 |
| **Urbanity** |  |  |  |  |  |  |  |  |  |
| 1 |  |  |  |  |  |  | 1 | 1 | 1 |
| 2 |  |  |  |  |  |  | 1.01 | 1.00 | 1.02 |
| 3 |  |  |  |  |  |  | 1.01 | 1.00 | 1.02 |
| 4 |  |  |  |  |  |  | 0.99 | 0.98 | 1.00 |
| 5 |  |  |  |  |  |  | 1.05 | 1.04 | 1.06 |
| 6 |  |  |  |  |  |  | 1.11 | 1.09 | 1.12 |
| **Household size** |  |  |  |  |  |  |  |  |  |
| 1 |  |  |  |  |  |  | 1 | 1 | 1 |
| 2 |  |  |  |  |  |  | 1.23 | 1.21 | 1.24 |
| 3-4 |  |  |  |  |  |  | 1.40 | 1.39 | 1.41 |
| >4 |  |  |  |  |  |  | 1.84 | 1.82 | 1.86 |
| **Country of origin** |  |  |  |  |  |  |  |  |  |
| Dutch |  |  |  |  |  |  | 1 | 1 | 1 |
| Europe |  |  |  |  |  |  | 0.99 | 0.98 | 1.01 |
| Other |  |  |  |  |  |  | 1.69 | 1.68 | 1.71 |

Table A 1 Positive COVID-19 tests 19 among individuals with at least one administered test by lagged income decile

|  | Model 1 | | | Model 2 | | | Model 3 | | |
| --- | --- | --- | --- | --- | --- | --- | --- | --- | --- |
|  | RR | 95% CI | | RR | 95% CI | | RR | 95% CI | |
| **Lagged income decile** |  |  |  |  |  |  |  |  |  |
| 1 | 1.38 | 1.36 | 1.40 | 1.39 | 1.37 | 1.40 | 1.27 | 1.25 | 1.29 |
| 2 | 1.27 | 1.25 | 1.29 | 1.27 | 1.26 | 1.29 | 1.24 | 1.22 | 1.25 |
| 3 | 1.24 | 1.22 | 1.25 | 1.24 | 1.22 | 1.26 | 1.22 | 1.21 | 1.24 |
| 4 | 1.22 | 1.20 | 1.23 | 1.22 | 1.20 | 1.23 | 1.22 | 1.20 | 1.23 |
| 5 | 1.20 | 1.18 | 1.21 | 1.20 | 1.18 | 1.21 | 1.20 | 1.19 | 1.22 |
| 6 | 1.17 | 1.15 | 1.18 | 1.17 | 1.15 | 1.18 | 1.18 | 1.16 | 1.19 |
| 7 | 1.14 | 1.12 | 1.15 | 1.14 | 1.12 | 1.15 | 1.15 | 1.14 | 1.17 |
| 8 | 1.09 | 1.08 | 1.11 | 1.09 | 1.08 | 1.11 | 1.11 | 1.09 | 1.12 |
| 9 | 1.05 | 1.03 | 1.06 | 1.05 | 1.03 | 1.06 | 1.06 | 1.05 | 1.07 |
| 10 | 1 | 1 | 1 | 1 | 1 | 1 | 1 | 1 | 1 |
| **Sex** |  |  |  |  |  |  |  |  |  |
| Male | 1 | 1 | 1 | 1 | 1 | 1 | 1 | 1 | 1 |
| Female | 0.87 | 0.87 | 0.88 | 0.88 | 0.87 | 0.88 | 0.89 | 0.88 | 0.89 |
| **Age** |  |  |  |  |  |  |  |  |  |
| 25-44 | 1 | 1 | 1 | 1 | 1 | 1 | 1 | 1 | 1 |
| 45-64 | 1.34 | 1.33 | 1.35 | 1.35 | 1.35 | 1.36 | 1.41 | 1.41 | 1.42 |
| 65-79 | 1.17 | 1.16 | 1.18 | 1.20 | 1.18 | 1.21 | 1.44 | 1.43 | 1.46 |
| **Comorbidities** |  |  |  |  |  |  |  |  |  |
| No |  |  |  | 1 | 1 | 1 | 1 | 1 | 1 |
| Yes |  |  |  | 0.94 | 0.94 | 0.95 | 0.95 | 0.94 | 0.95 |
| **Urbanity** |  |  |  |  |  |  |  |  |  |
| 1 |  |  |  |  |  |  | 1 | 1 | 1 |
| 2 |  |  |  |  |  |  | 1.01 | 1.00 | 1.02 |
| 3 |  |  |  |  |  |  | 1.01 | 1.00 | 1.02 |
| 4 |  |  |  |  |  |  | 0.99 | 0.98 | 1.00 |
| 5 |  |  |  |  |  |  | 1.05 | 1.04 | 1.06 |
| 6 |  |  |  |  |  |  | 1.11 | 1.09 | 1.12 |
| **Household size** |  |  |  |  |  |  |  |  |  |
| 1 |  |  |  |  |  |  | 1 | 1 | 1 |
| 2 |  |  |  |  |  |  | 1.22 | 1.20 | 1.23 |
| 3-4 |  |  |  |  |  |  | 1.39 | 1.37 | 1.40 |
| >4 |  |  |  |  |  |  | 1.82 | 1.80 | 1.84 |
| **Country of origin** |  |  |  |  |  |  |  |  |  |
| Dutch |  |  |  |  |  |  | 1 | 1 | 1 |
| Europe |  |  |  |  |  |  | 0.99 | 0.98 | 1.01 |
| Other |  |  |  |  |  |  | 1.70 | 1.69 | 1.71 |

Table A 10 Positive COVID-19 tests among individuals with at least one administered test by financial wealth decile

|  | Model 1 | | | Model 2 | | | Model 3 | | |
| --- | --- | --- | --- | --- | --- | --- | --- | --- | --- |
|  | RR | 95% CI | | RR | 95% CI | | RR | 95% CI | |
| **Financial wealth decile** |  |  |  |  |  |  |  |  |  |
| 1 | 1.13 | 1.11 | 1.14 | 1.13 | 1.12 | 1.15 | 1.08 | 1.06 | 1.09 |
| 2 | 1.15 | 1.14 | 1.16 | 1.16 | 1.14 | 1.17 | 1.15 | 1.14 | 1.17 |
| 3 | 1.13 | 1.11 | 1.14 | 1.13 | 1.12 | 1.15 | 1.15 | 1.14 | 1.17 |
| 4 | 1.07 | 1.05 | 1.08 | 1.07 | 1.06 | 1.08 | 1.11 | 1.09 | 1.12 |
| 5 | 1.03 | 1.02 | 1.04 | 1.03 | 1.02 | 1.05 | 1.08 | 1.06 | 1.09 |
| 6 | 1.05 | 1.03 | 1.06 | 1.05 | 1.03 | 1.06 | 1.09 | 1.08 | 1.11 |
| 7 | 1.04 | 1.03 | 1.05 | 1.04 | 1.03 | 1.05 | 1.08 | 1.07 | 1.10 |
| 8 | 1.02 | 1.01 | 1.03 | 1.02 | 1.01 | 1.03 | 1.06 | 1.05 | 1.07 |
| 9 | 1.00 | 0.98 | 1.01 | 1.00 | 0.98 | 1.01 | 1.02 | 1.01 | 1.03 |
| 10 | 1 | 1 | 1 | 1 | 1 | 1 | 1 | 1 | 1 |
| **Sex** |  |  |  |  |  |  |  |  |  |
| Male | 1 | 1 | 1 | 1 | 1 | 1 | 1 | 1 | 1 |
| Female | 0.88 | 0.88 | 0.88 | 0.88 | 0.88 | 0.89 | 0.89 | 0.89 | 0.90 |
| **Age** |  |  |  |  |  |  |  |  |  |
| 25-44 | 1 | 1 | 1 | 1 | 1 | 1 | 1 | 1 | 1 |
| 45-64 | 1.34 | 1.33 | 1.34 | 1.35 | 1.34 | 1.36 | 1.41 | 1.40 | 1.42 |
| 65-79 | 1.16 | 1.15 | 1.17 | 1.19 | 1.17 | 1.20 | 1.44 | 1.42 | 1.45 |
| **Comorbidities** |  |  |  |  |  |  |  |  |  |
| No |  |  |  | 1 | 1 | 1 | 1 | 1 | 1 |
| Yes |  |  |  | 0.95 | 0.94 | 0.95 | 0.95 | 0.94 | 0.96 |
| **Urbanity** |  |  |  |  |  |  |  |  |  |
| 1 |  |  |  |  |  |  | 1 | 1 | 1 |
| 2 |  |  |  |  |  |  | 1.01 | 0.99 | 1.02 |
| 3 |  |  |  |  |  |  | 1.00 | 0.99 | 1.01 |
| 4 |  |  |  |  |  |  | 0.99 | 0.98 | 1.00 |
| 5 |  |  |  |  |  |  | 1.04 | 1.03 | 1.06 |
| 6 |  |  |  |  |  |  | 1.10 | 1.08 | 1.11 |
| **Household size** |  |  |  |  |  |  |  |  |  |
| 1 |  |  |  |  |  |  | 1 | 1 | 1 |
| 2 |  |  |  |  |  |  | 1.20 | 1.19 | 1.21 |
| 3-4 |  |  |  |  |  |  | 1.38 | 1.37 | 1.40 |
| >4 |  |  |  |  |  |  | 1.84 | 1.82 | 1.86 |
| **Country of origin** |  |  |  |  |  |  |  |  |  |
| Dutch |  |  |  |  |  |  | 1 | 1 | 1 |
| Europe |  |  |  |  |  |  | 0.99 | 0.98 | 1.00 |
| Other |  |  |  |  |  |  | 1.71 | 1.70 | 1.72 |

Table A 11 Positive COVID-19 tests among individuals with at least one administered test by education

|  | Model 1 | | | Model 2 | | | Model 3 | | |
| --- | --- | --- | --- | --- | --- | --- | --- | --- | --- |
|  | RR | 95% CI | | RR | 95% CI | | RR | 95% CI | |
| **Education** |  |  |  |  |  |  |  |  |  |
| Low | 1.53 | 1.52 | 1.55 | 1.54 | 1.53 | 1.56 | 1.41 | 1.40 | 1.42 |
| Middle | 1.25 | 1.24 | 1.26 | 1.25 | 1.25 | 1.26 | 1.24 | 1.24 | 1.25 |
| High | 1 | 1 | 1 | 1 | 1 | 1 | 1 | 1 | 1 |
| **Sex** |  |  |  |  |  |  |  |  |  |
| Male | 1 | 1 | 1 | 1 | 1 | 1 | 1 | 1 | 1 |
| Female | 0.89 | 0.88 | 0.89 | 0.89 | 0.88 | 0.90 | 0.89 | 0.89 | 0.90 |
| **Age** |  |  |  |  |  |  |  |  |  |
| 25-44 | 1 | 1 | 1 | 1 | 1 | 1 | 1 | 1 | 1 |
| 45-64 | 1.26 | 1.25 | 1.26 | 1.27 | 1.26 | 1.28 | 1.32 | 1.31 | 1.33 |
| 65-79 | 1.02 | 1.00 | 1.03 | 1.04 | 1.02 | 1.05 | 1.25 | 1.23 | 1.27 |
| **Comorbidities** |  |  |  |  |  |  |  |  |  |
| No |  |  |  | 1 | 1 | 1 | 1 | 1 | 1 |
| Yes |  |  |  | 0.95 | 0.94 | 0.95 | 0.95 | 0.94 | 0.95 |
| **Urbanity** |  |  |  |  |  |  |  |  |  |
| 1 |  |  |  |  |  |  | 1 | 1 | 1 |
| 2 |  |  |  |  |  |  | 1.03 | 1.02 | 1.04 |
| 3 |  |  |  |  |  |  | 1.03 | 1.02 | 1.05 |
| 4 |  |  |  |  |  |  | 1.03 | 1.02 | 1.04 |
| 5 |  |  |  |  |  |  | 1.11 | 1.10 | 1.13 |
| 6 |  |  |  |  |  |  | 1.21 | 1.19 | 1.23 |
| **Household size** |  |  |  |  |  |  |  |  |  |
| 1 |  |  |  |  |  |  | 1 | 1 | 1 |
| 2 |  |  |  |  |  |  | 1.18 | 1.17 | 1.20 |
| 3-4 |  |  |  |  |  |  | 1.32 | 1.31 | 1.34 |
| >4 |  |  |  |  |  |  | 1.73 | 1.71 | 1.76 |
| **Country of origin** |  |  |  |  |  |  |  |  |  |
| Dutch |  |  |  |  |  |  | 1 | 1 | 1 |
| Europe |  |  |  |  |  |  | 1.00 | 0.99 | 1.02 |
| Other |  |  |  |  |  |  | 1.74 | 1.72 | 1.75 |

Table A 12 Positive COVID-19 tests among individuals with at least one administered test by imputed education

|  | Model 1 | | | Model 2 | | | Model 3 | | |
| --- | --- | --- | --- | --- | --- | --- | --- | --- | --- |
|  | RR | 95% CI | | RR | 95% CI | | RR | 95% CI | |
| **Imputed education** |  |  |  |  |  |  |  |  |  |
| Low | 1.45 | 1.44 | 1.47 | 1.46 | 1.45 | 1.48 | 1.39 | 1.37 | 1.40 |
| Middle | 1.24 | 1.23 | 1.25 | 1.25 | 1.24 | 1.26 | 1.24 | 1.23 | 1.25 |
| High | 1 | 1 | 1 | 1 | 1 | 1 | 1 | 1 | 1 |
| **Sex** |  |  |  |  |  |  |  |  |  |
| Male | 1 | 1 | 1 | 1 | 1 | 1 | 1 | 1 | 1 |
| Female | 0.87 | 0.87 | 0.88 | 0.88 | 0.87 | 0.88 | 0.89 | 0.88 | 0.89 |
| **Age** |  |  |  |  |  |  |  |  |  |
| 25-44 | 1 | 1 | 1 | 1 | 1 | 1 | 1 | 1 | 1 |
| 45-64 | 1.27 | 1.26 | 1.28 | 1.29 | 1.28 | 1.30 | 1.37 | 1.36 | 1.38 |
| 65-79 | 1.05 | 1.04 | 1.06 | 1.08 | 1.07 | 1.09 | 1.33 | 1.32 | 1.35 |
| **Comorbidities** |  |  |  |  |  |  |  |  |  |
| No |  |  |  | 1 | 1 | 1 | 1 | 1 | 1 |
| Yes |  |  |  | 0.93 | 0.92 | 0.94 | 0.93 | 0.93 | 0.94 |
| **Urbanity** |  |  |  |  |  |  |  |  |  |
| 1 |  |  |  |  |  |  | 1 | 1 | 1 |
| 2 |  |  |  |  |  |  | 1.02 | 1.01 | 1.03 |
| 3 |  |  |  |  |  |  | 1.02 | 1.01 | 1.03 |
| 4 |  |  |  |  |  |  | 1.01 | 1.00 | 1.02 |
| 5 |  |  |  |  |  |  | 1.08 | 1.07 | 1.09 |
| 6 |  |  |  |  |  |  | 1.16 | 1.14 | 1.18 |
| **Household size** |  |  |  |  |  |  |  |  |  |
| 1 |  |  |  |  |  |  | 1 | 1 | 1 |
| 2 |  |  |  |  |  |  | 1.18 | 1.17 | 1.19 |
| 3-4 |  |  |  |  |  |  | 1.37 | 1.35 | 1.38 |
| >4 |  |  |  |  |  |  | 1.80 | 1.78 | 1.82 |
| **Country of origin** |  |  |  |  |  |  |  |  |  |
| Dutch |  |  |  |  |  |  | 1 | 1 | 1 |
| Europe |  |  |  |  |  |  | 0.99 | 0.98 | 1.00 |
| Other |  |  |  |  |  |  | 1.66 | 1.65 | 1.67 |

## GP consultation for COVID-19 related symptoms – Income, financial wealth, education and imputed education

Table A 13 GP consultation for COVID-19 related symptoms by income decile

|  | Model 1 | | | Model 2 | | | Model 3 | | |
| --- | --- | --- | --- | --- | --- | --- | --- | --- | --- |
|  | RR | 95% CI | | RR | 95% CI | | RR | 95% CI | |
| **Income decile** |  |  |  |  |  |  |  |  |  |
| 1 | 1.06 | 0.97 | 1.16 | 1.04 | 0.95 | 1.14 | 1.08 | 0.98 | 1.18 |
| 2 | 1.14 | 1.04 | 1.24 | 1.12 | 1.03 | 1.23 | 1.16 | 1.06 | 1.27 |
| 3 | 1.21 | 1.11 | 1.32 | 1.21 | 1.10 | 1.31 | 1.22 | 1.12 | 1.34 |
| 4 | 1.21 | 1.11 | 1.32 | 1.20 | 1.10 | 1.31 | 1.20 | 1.10 | 1.31 |
| 5 | 1.19 | 1.09 | 1.30 | 1.18 | 1.09 | 1.29 | 1.18 | 1.08 | 1.29 |
| 6 | 1.28 | 1.18 | 1.40 | 1.28 | 1.18 | 1.39 | 1.27 | 1.17 | 1.39 |
| 7 | 1.28 | 1.18 | 1.39 | 1.28 | 1.17 | 1.39 | 1.27 | 1.17 | 1.38 |
| 8 | 1.22 | 1.12 | 1.33 | 1.22 | 1.12 | 1.33 | 1.20 | 1.11 | 1.31 |
| 9 | 1.14 | 1.05 | 1.25 | 1.14 | 1.05 | 1.24 | 1.14 | 1.04 | 1.24 |
| 10 | 1 | 1 | 1 | 1 | 1 | 1 | 1 | 1 | 1 |
| **Sex** |  |  |  |  |  |  |  |  |  |
| Male | 1 | 1 | 1 | 1 | 1 | 1 | 1 | 1 | 1 |
| Female | 1.23 | 1.19 | 1.28 | 1.21 | 1.17 | 1.26 | 1.21 | 1.16 | 1.26 |
| **Age** |  |  |  |  |  |  |  |  |  |
| 25-44 | 1 | 1 | 1 | 1 | 1 | 1 | 1 | 1 | 1 |
| 45-64 | 1.14 | 1.09 | 1.18 | 1.09 | 1.04 | 1.13 | 1.15 | 1.11 | 1.20 |
| 65-79 | 0.70 | 0.66 | 0.74 | 0.64 | 0.61 | 0.68 | 0.83 | 0.78 | 0.88 |
| **Comorbidities** |  |  |  |  |  |  |  |  |  |
| No |  |  |  | 1 | 1 | 1 | 1 | 1 | 1 |
| Yes |  |  |  | 1.27 | 1.22 | 1.32 | 1.27 | 1.23 | 1.32 |
| **Urbanity** |  |  |  |  |  |  |  |  |  |
| 1 |  |  |  |  |  |  | 1 | 1 | 1 |
| 2 |  |  |  |  |  |  | 1.45 | 1.06 | 1.98 |
| 3 |  |  |  |  |  |  | 1.48 | 1.11 | 1.97 |
| 4 |  |  |  |  |  |  | 1.45 | 1.11 | 1.91 |
| 5 |  |  |  |  |  |  | 1.31 | 0.99 | 1.72 |
| 6 |  |  |  |  |  |  | 1.43 | 1.08 | 1.89 |
| **Household size** |  |  |  |  |  |  |  |  |  |
| 1 |  |  |  |  |  |  | 1 | 1 | 1 |
| 2 |  |  |  |  |  |  | 1.18 | 1.11 | 1.25 |
| 3-4 |  |  |  |  |  |  | 1.58 | 1.49 | 1.68 |
| >4 |  |  |  |  |  |  | 2.12 | 1.97 | 2.28 |
| **Country of origin** |  |  |  |  |  |  |  |  |  |
| Dutch |  |  |  |  |  |  | 1 | 1 | 1 |
| Europe |  |  |  |  |  |  | 0.79 | 0.72 | 0.86 |
| Other |  |  |  |  |  |  | 1.19 | 1.14 | 1.25 |

Table A 1 GP consultations for COVID-19 related symptoms by lagged income decile

|  | Model 1 | | | Model 2 | | | Model 3 | | |
| --- | --- | --- | --- | --- | --- | --- | --- | --- | --- |
|  | RR | 95% CI | | RR | 95% CI | | RR | 95% CI | |
| **Lagged income decile** |  |  |  |  |  |  |  |  |  |
| 1 | 1.11 | 1.01 | 1.21 | 1.09 | 0.99 | 1.19 | 1.10 | 1.01 | 1.21 |
| 2 | 1.12 | 1.02 | 1.22 | 1.11 | 1.01 | 1.21 | 1.12 | 1.02 | 1.23 |
| 3 | 1.18 | 1.08 | 1.29 | 1.17 | 1.07 | 1.28 | 1.17 | 1.07 | 1.28 |
| 4 | 1.21 | 1.11 | 1.32 | 1.20 | 1.10 | 1.32 | 1.20 | 1.09 | 1.31 |
| 5 | 1.22 | 1.11 | 1.33 | 1.21 | 1.11 | 1.33 | 1.20 | 1.10 | 1.31 |
| 6 | 1.29 | 1.18 | 1.41 | 1.28 | 1.18 | 1.40 | 1.27 | 1.16 | 1.39 |
| 7 | 1.22 | 1.11 | 1.33 | 1.21 | 1.11 | 1.33 | 1.21 | 1.10 | 1.32 |
| 8 | 1.17 | 1.07 | 1.28 | 1.17 | 1.07 | 1.28 | 1.17 | 1.06 | 1.28 |
| 9 | 1.11 | 1.01 | 1.22 | 1.11 | 1.01 | 1.22 | 1.11 | 1.01 | 1.22 |
| 10 | 1 | 1 | 1 | 1 | 1 | 1 | 1 | 1 | 1 |
| **Sex** |  |  |  |  |  |  |  |  |  |
| Male | 1 | 1 | 1 | 1 | 1 | 1 | 1 | 1 | 1 |
| Female | 1.23 | 1.19 | 1.28 | 1.21 | 1.17 | 1.26 | 1.21 | 1.17 | 1.26 |
| **Age** |  |  |  |  |  |  |  |  |  |
| 25-44 | 1 | 1 | 1 | 1 | 1 | 1 | 1 | 1 | 1 |
| 45-64 | 1.14 | 1.09 | 1.18 | 1.09 | 1.04 | 1.13 | 1.15 | 1.10 | 1.20 |
| 65-79 | 0.70 | 0.66 | 0.74 | 0.65 | 0.61 | 0.68 | 0.83 | 0.78 | 0.88 |
| **Comorbidities** |  |  |  |  |  |  |  |  |  |
| No |  |  |  | 1 | 1 | 1 | 1 | 1 | 1 |
| Yes |  |  |  | 1.27 | 1.22 | 1.32 | 1.27 | 1.23 | 1.32 |
| **Urbanity** |  |  |  |  |  |  |  |  |  |
| 1 |  |  |  |  |  |  | 1 | 1 | 1 |
| 2 |  |  |  |  |  |  | 1.45 | 1.06 | 1.98 |
| 3 |  |  |  |  |  |  | 1.48 | 1.11 | 1.98 |
| 4 |  |  |  |  |  |  | 1.46 | 1.11 | 1.92 |
| 5 |  |  |  |  |  |  | 1.31 | 0.99 | 1.72 |
| 6 |  |  |  |  |  |  | 1.43 | 1.09 | 1.90 |
| **Household size** |  |  |  |  |  |  |  |  |  |
| 1 |  |  |  |  |  |  | 1 | 1 | 1 |
| 2 |  |  |  |  |  |  | 1.18 | 1.11 | 1.25 |
| 3-4 |  |  |  |  |  |  | 1.58 | 1.49 | 1.68 |
| >4 |  |  |  |  |  |  | 2.12 | 1.97 | 2.28 |
| **Country of origin** |  |  |  |  |  |  |  |  |  |
| Dutch |  |  |  |  |  |  | 1 | 1 | 1 |
| Europe |  |  |  |  |  |  | 0.79 | 0.72 | 0.86 |
| Other |  |  |  |  |  |  | 1.19 | 1.14 | 1.24 |

Table A 14 GP consultation for COVID-19 related symptoms by financial wealth decile

|  | Model 1 | | | Model 2 | | | Model 3 | | |
| --- | --- | --- | --- | --- | --- | --- | --- | --- | --- |
|  | RR | 95% CI | | RR | 95% CI | | RR | 95% CI | |
| **Financial wealth decile** |  |  |  |  |  |  |  |  |  |
| 1 | 1.16 | 1.07 | 1.27 | 1.14 | 1.04 | 1.24 | 1.19 | 1.09 | 1.30 |
| 2 | 1.04 | 0.95 | 1.14 | 1.02 | 0.93 | 1.11 | 1.16 | 1.06 | 1.27 |
| 3 | 0.98 | 0.90 | 1.08 | 0.97 | 0.89 | 1.06 | 1.08 | 0.99 | 1.18 |
| 4 | 1.11 | 1.02 | 1.22 | 1.10 | 1.01 | 1.20 | 1.21 | 1.10 | 1.32 |
| 5 | 1.08 | 0.99 | 1.18 | 1.07 | 0.98 | 1.17 | 1.16 | 1.06 | 1.27 |
| 6 | 1.14 | 1.05 | 1.25 | 1.14 | 1.04 | 1.24 | 1.20 | 1.10 | 1.32 |
| 7 | 1.10 | 1.01 | 1.20 | 1.09 | 1.00 | 1.20 | 1.14 | 1.04 | 1.24 |
| 8 | 1.08 | 0.99 | 1.18 | 1.08 | 0.99 | 1.18 | 1.11 | 1.01 | 1.21 |
| 9 | 0.99 | 0.90 | 1.08 | 0.99 | 0.90 | 1.08 | 1.00 | 0.92 | 1.10 |
| 10 | 1 | 1 | 1 | 1 | 1 | 1 | 1 | 1 | 1 |
| **Sex** |  |  |  |  |  |  |  |  |  |
| Male | 1 | 1 | 1 | 1 | 1 | 1 | 1 | 1 | 1 |
| Female | 1.23 | 1.19 | 1.28 | 1.21 | 1.17 | 1.26 | 1.21 | 1.17 | 1.26 |
| **Age** |  |  |  |  |  |  |  |  |  |
| 25-44 | 1 | 1 | 1 | 1 | 1 | 1 | 1 | 1 | 1 |
| 45-64 | 1.13 | 1.09 | 1.18 | 1.09 | 1.04 | 1.13 | 1.15 | 1.10 | 1.19 |
| 65-79 | 0.70 | 0.66 | 0.74 | 0.64 | 0.61 | 0.68 | 0.83 | 0.78 | 0.88 |
| **Comorbidities** |  |  |  |  |  |  |  |  |  |
| No |  |  |  | 1 | 1 | 1 | 1 | 1 | 1 |
| Yes |  |  |  | 1.26 | 1.22 | 1.31 | 1.27 | 1.22 | 1.32 |
| **Urbanity** |  |  |  |  |  |  |  |  |  |
| 1 |  |  |  |  |  |  | 1 | 1 | 1 |
| 2 |  |  |  |  |  |  | 1.44 | 1.06 | 1.97 |
| 3 |  |  |  |  |  |  | 1.49 | 1.12 | 1.99 |
| 4 |  |  |  |  |  |  | 1.46 | 1.11 | 1.92 |
| 5 |  |  |  |  |  |  | 1.29 | 0.98 | 1.70 |
| 6 |  |  |  |  |  |  | 1.42 | 1.07 | 1.87 |
| **Household size** |  |  |  |  |  |  |  |  |  |
| 1 |  |  |  |  |  |  | 1 | 1 | 1 |
| 2 |  |  |  |  |  |  | 1.19 | 1.12 | 1.27 |
| 3-4 |  |  |  |  |  |  | 1.62 | 1.53 | 1.72 |
| >4 |  |  |  |  |  |  | 2.17 | 2.02 | 2.33 |
| **Country of origin** |  |  |  |  |  |  |  |  |  |
| Dutch |  |  |  |  |  |  | 1 | 1 | 1 |
| Europe |  |  |  |  |  |  | 0.78 | 0.71 | 0.85 |
| Other |  |  |  |  |  |  | 1.16 | 1.11 | 1.21 |

Table A 15 GP consultation for COVID-19 related symptoms by education

|  | Model 1 | | | Model 2 | | | Model 3 | | |
| --- | --- | --- | --- | --- | --- | --- | --- | --- | --- |
|  | RR | 95% CI | | RR | 95% CI | | RR | 95% CI | |
| **Education** |  |  |  |  |  |  |  |  |  |
| Low | 1.12 | 1.05 | 1.19 | 1.09 | 1.02 | 1.16 | 1.06 | 1.00 | 1.13 |
| Middle | 1.16 | 1.10 | 1.22 | 1.14 | 1.08 | 1.20 | 1.14 | 1.08 | 1.20 |
| High | 1 | 1 | 1 | 1 | 1 | 1 | 1 | 1 | 1 |
| **Sex** |  |  |  |  |  |  |  |  |  |
| Male | 1 | 1 | 1 | 1 | 1 | 1 | 1 | 1 | 1 |
| Female | 1.30 | 1.24 | 1.36 | 1.27 | 1.21 | 1.33 | 1.26 | 1.20 | 1.32 |
| **Age** |  |  |  |  |  |  |  |  |  |
| 25-44 | 1 | 1 | 1 | 1 | 1 | 1 | 1 | 1 | 1 |
| 45-64 | 1.13 | 1.08 | 1.19 | 1.09 | 1.04 | 1.15 | 1.14 | 1.09 | 1.20 |
| 65-79 | 0.69 | 0.62 | 0.75 | 0.64 | 0.58 | 0.70 | 0.80 | 0.72 | 0.88 |
| **Comorbidities** |  |  |  |  |  |  |  |  |  |
| No |  |  |  | 1 | 1 | 1 | 1 | 1 | 1 |
| Yes |  |  |  | 1.25 | 1.19 | 1.31 | 1.26 | 1.20 | 1.32 |
| **Urbanity** |  |  |  |  |  |  |  |  |  |
| 1 |  |  |  |  |  |  | 1 | 1 | 1 |
| 2 |  |  |  |  |  |  | 1.15 | 0.81 | 1.63 |
| 3 |  |  |  |  |  |  | 1.13 | 0.82 | 1.55 |
| 4 |  |  |  |  |  |  | 1.12 | 0.83 | 1.51 |
| 5 |  |  |  |  |  |  | 1.01 | 0.75 | 1.36 |
| 6 |  |  |  |  |  |  | 1.11 | 0.82 | 1.51 |
| **Household size** |  |  |  |  |  |  |  |  |  |
| 1 |  |  |  |  |  |  | 1 | 1 | 1 |
| 2 |  |  |  |  |  |  | 1.26 | 1.17 | 1.36 |
| 3-4 |  |  |  |  |  |  | 1.64 | 1.52 | 1.76 |
| >4 |  |  |  |  |  |  | 2.09 | 1.91 | 2.28 |
| **Country of origin** |  |  |  |  |  |  |  |  |  |
| Dutch |  |  |  |  |  |  | 1 | 1 | 1 |
| Europe |  |  |  |  |  |  | 0.84 | 0.75 | 0.94 |
| Other |  |  |  |  |  |  | 1.21 | 1.15 | 1.28 |

Table A 16 GP consultation for COVID-19 related symptoms by imputed education

|  | Model 1 | | | Model 2 | | | Model 3 | | |
| --- | --- | --- | --- | --- | --- | --- | --- | --- | --- |
|  | RR | 95% CI | | RR | 95% CI | | RR | 95% CI | |
| **Imputed education** |  |  |  |  |  |  |  |  |  |
| Low | 1.13 | 1.05 | 1.22 | 1.11 | 1.04 | 1.19 | 1.09 | 1.03 | 1.16 |
| Middle | 1.14 | 1.08 | 1.21 | 1.13 | 1.07 | 1.20 | 1.12 | 1.07 | 1.18 |
| High | 1 | 1 | 1 | 1 | 1 | 1 | 1 | 1 | 1 |
| **Sex** |  |  |  |  |  |  |  |  |  |
| Male | 1 | 1 | 1 | 1 | 1 | 1 | 1 | 1 | 1 |
| Female | 1.23 | 1.18 | 1.28 | 1.21 | 1.16 | 1.25 | 1.21 | 1.16 | 1.25 |
| **Age** |  |  |  |  |  |  |  |  |  |
| 25-44 | 1 | 1 | 1 | 1 | 1 | 1 | 1 | 1 | 1 |
| 45-64 | 1.12 | 1.07 | 1.18 | 1.08 | 1.03 | 1.13 | 1.15 | 1.10 | 1.19 |
| 65-79 | 0.68 | 0.64 | 0.73 | 0.63 | 0.59 | 0.67 | 0.82 | 0.77 | 0.87 |
| **Comorbidities** |  |  |  |  |  |  |  |  |  |
| No |  |  |  | 1 | 1 | 1 | 1 | 1 | 1 |
| Yes |  |  |  | 1.26 | 1.21 | 1.31 | 1.27 | 1.22 | 1.32 |
| **Urbanity** |  |  |  |  |  |  |  |  |  |
| 1 |  |  |  |  |  |  | 1 | 1 | 1 |
| 2 |  |  |  |  |  |  | 1.47 | 1.08 | 2.01 |
| 3 |  |  |  |  |  |  | 1.51 | 1.14 | 2.02 |
| 4 |  |  |  |  |  |  | 1.49 | 1.13 | 1.96 |
| 5 |  |  |  |  |  |  | 1.33 | 1.01 | 1.76 |
| 6 |  |  |  |  |  |  | 1.47 | 1.11 | 1.94 |
| **Household size** |  |  |  |  |  |  |  |  |  |
| 1 |  |  |  |  |  |  | 1 | 1 | 1 |
| 2 |  |  |  |  |  |  | 1.18 | 1.11 | 1.25 |
| 3-4 |  |  |  |  |  |  | 1.59 | 1.50 | 1.69 |
| >4 |  |  |  |  |  |  | 2.12 | 1.98 | 2.28 |
| **Country of origin** |  |  |  |  |  |  |  |  |  |
| Dutch |  |  |  |  |  |  | 1 | 1 | 1 |
| Europe |  |  |  |  |  |  | 0.79 | 0.72 | 0.86 |
| Other |  |  |  |  |  |  | 1.17 | 1.12 | 1.23 |

## Hospital admissions for COVID-19 – Income, financial wealth, education and imputed education

Table A 17 Hospital admissions for COVID-19 by income decile

|  | Model 1 | | | Model 2 | | | Model 3 | | |
| --- | --- | --- | --- | --- | --- | --- | --- | --- | --- |
|  | RR | 95% CI | | RR | 95% CI | | RR | 95% CI | |
| **Income decile** |  |  |  |  |  |  |  |  |  |
| 1 | 3.08 | 2.86 | 3.31 | 2.98 | 2.77 | 3.21 | 2.11 | 1.95 | 2.27 |
| 2 | 2.27 | 2.10 | 2.45 | 2.20 | 2.03 | 2.37 | 1.94 | 1.79 | 2.09 |
| 3 | 1.89 | 1.74 | 2.04 | 1.85 | 1.71 | 2.00 | 1.77 | 1.64 | 1.92 |
| 4 | 1.61 | 1.49 | 1.75 | 1.59 | 1.47 | 1.72 | 1.59 | 1.47 | 1.73 |
| 5 | 1.51 | 1.39 | 1.64 | 1.50 | 1.38 | 1.62 | 1.53 | 1.41 | 1.66 |
| 6 | 1.50 | 1.38 | 1.63 | 1.49 | 1.37 | 1.61 | 1.53 | 1.41 | 1.66 |
| 7 | 1.27 | 1.17 | 1.39 | 1.26 | 1.16 | 1.38 | 1.30 | 1.20 | 1.42 |
| 8 | 1.19 | 1.09 | 1.30 | 1.19 | 1.09 | 1.29 | 1.22 | 1.12 | 1.33 |
| 9 | 1.08 | 0.99 | 1.18 | 1.08 | 0.99 | 1.18 | 1.10 | 1.01 | 1.20 |
| 10 | 1 | 1 | 1 | 1 | 1 | 1 | 1 | 1 | 1 |
| **Sex** |  |  |  |  |  |  |  |  |  |
| Male | 1 | 1 | 1 | 1 | 1 | 1 | 1 | 1 | 1 |
| Female | 0.62 | 0.60 | 0.64 | 0.60 | 0.58 | 0.62 | 0.61 | 0.59 | 0.63 |
| **Age** |  |  |  |  |  |  |  |  |  |
| 25-44 | 1 | 1 | 1 | 1 | 1 | 1 | 1 | 1 | 1 |
| 45-64 | 4.05 | 3.82 | 4.29 | 3.44 | 3.24 | 3.65 | 4.07 | 3.84 | 4.31 |
| 65-79 | 8.90 | 8.41 | 9.43 | 6.51 | 6.14 | 6.91 | 9.78 | 9.19 | 10.41 |
| **Comorbidities** |  |  |  |  |  |  |  |  |  |
| No |  |  |  | 1 | 1 | 1 | 1 | 1 | 1 |
| Yes |  |  |  | 2.39 | 2.30 | 2.48 | 2.40 | 2.31 | 2.49 |
| **Urbanity** |  |  |  |  |  |  |  |  |  |
| 1 |  |  |  |  |  |  | 1 | 1 | 1 |
| 2 |  |  |  |  |  |  | 1.18 | 1.11 | 1.27 |
| 3 |  |  |  |  |  |  | 1.35 | 1.27 | 1.44 |
| 4 |  |  |  |  |  |  | 1.43 | 1.34 | 1.52 |
| 5 |  |  |  |  |  |  | 1.71 | 1.60 | 1.82 |
| 6 |  |  |  |  |  |  | 1.96 | 1.81 | 2.12 |
| **Household size** |  |  |  |  |  |  |  |  |  |
| 1 |  |  |  |  |  |  | 1 | 1 | 1 |
| 2 |  |  |  |  |  |  | 1.29 | 1.23 | 1.34 |
| 3-4 |  |  |  |  |  |  | 1.48 | 1.41 | 1.56 |
| >4 |  |  |  |  |  |  | 1.95 | 1.82 | 2.09 |
| **Country of origin** |  |  |  |  |  |  |  |  |  |
| Dutch |  |  |  |  |  |  | 1 | 1 | 1 |
| Europe |  |  |  |  |  |  | 1.02 | 0.95 | 1.10 |
| Other |  |  |  |  |  |  | 3.08 | 2.97 | 3.21 |

Table A 1 Hospital admissions for COVID-19 by lagged income decile

|  | Model 1 | | | Model 2 | | | Model 3 | | |
| --- | --- | --- | --- | --- | --- | --- | --- | --- | --- |
|  | RR | 95% CI | | RR | 95% CI | | RR | 95% CI | |
| **Lagged income decile** |  |  |  |  |  |  |  |  |  |
| 1 | 3.19 | 2.95 | 3.44 | 3.10 | 2.87 | 3.34 | 2.17 | 2.01 | 2.34 |
| 2 | 2.29 | 2.12 | 2.48 | 2.24 | 2.07 | 2.42 | 1.98 | 1.82 | 2.14 |
| 3 | 1.98 | 1.82 | 2.15 | 1.95 | 1.79 | 2.11 | 1.87 | 1.72 | 2.03 |
| 4 | 1.70 | 1.56 | 1.84 | 1.68 | 1.54 | 1.82 | 1.68 | 1.54 | 1.82 |
| 5 | 1.61 | 1.48 | 1.75 | 1.59 | 1.46 | 1.73 | 1.62 | 1.49 | 1.76 |
| 6 | 1.48 | 1.35 | 1.61 | 1.47 | 1.34 | 1.60 | 1.50 | 1.38 | 1.64 |
| 7 | 1.30 | 1.19 | 1.42 | 1.29 | 1.18 | 1.41 | 1.33 | 1.22 | 1.45 |
| 8 | 1.21 | 1.10 | 1.33 | 1.21 | 1.10 | 1.32 | 1.24 | 1.13 | 1.35 |
| 9 | 1.13 | 1.03 | 1.24 | 1.13 | 1.03 | 1.24 | 1.16 | 1.05 | 1.27 |
| 10 | 1 | 1 | 1 | 1 | 1 | 1 | 1 | 1 | 1 |
| **Sex** |  |  |  |  |  |  |  |  |  |
| Male | 1 | 1 | 1 | 1 | 1 | 1 | 1 | 1 | 1 |
| Female | 0.61 | 0.59 | 0.63 | 0.60 | 0.58 | 0.61 | 0.61 | 0.59 | 0.63 |
| **Age** |  |  |  |  |  |  |  |  |  |
| 25-44 | 1 | 1 | 1 | 1 | 1 | 1 | 1 | 1 | 1 |
| 45-64 | 4.09 | 3.86 | 4.33 | 3.47 | 3.27 | 3.68 | 4.08 | 3.85 | 4.33 |
| 65-79 | 8.99 | 8.48 | 9.52 | 6.58 | 6.20 | 6.98 | 9.82 | 9.22 | 10.45 |
| **Comorbidities** |  |  |  |  |  |  |  |  |  |
| No |  |  |  | 1 | 1 | 1 | 1 | 1 | 1 |
| Yes |  |  |  | 2.39 | 2.31 | 2.48 | 2.40 | 2.31 | 2.49 |
| **Urbanity** |  |  |  |  |  |  |  |  |  |
| 1 |  |  |  |  |  |  | 1 | 1 | 1 |
| 2 |  |  |  |  |  |  | 1.18 | 1.11 | 1.27 |
| 3 |  |  |  |  |  |  | 1.35 | 1.27 | 1.44 |
| 4 |  |  |  |  |  |  | 1.43 | 1.34 | 1.51 |
| 5 |  |  |  |  |  |  | 1.71 | 1.60 | 1.82 |
| 6 |  |  |  |  |  |  | 1.96 | 1.81 | 2.12 |
| **Household size** |  |  |  |  |  |  |  |  |  |
| 1 |  |  |  |  |  |  | 1 | 1 | 1 |
| 2 |  |  |  |  |  |  | 1.28 | 1.23 | 1.34 |
| 3-4 |  |  |  |  |  |  | 1.45 | 1.38 | 1.53 |
| >4 |  |  |  |  |  |  | 1.88 | 1.75 | 2.01 |
| **Country of origin** |  |  |  |  |  |  |  |  |  |
| Dutch |  |  |  |  |  |  | 1 | 1 | 1 |
| Europe |  |  |  |  |  |  | 1.02 | 0.95 | 1.10 |
| Other |  |  |  |  |  |  | 3.10 | 2.98 | 3.22 |

Table A 18 Hospital admissions for COVID-19 by financial wealth decile

|  | Model 1 | | | Model 2 | | | Model 3 | | |
| --- | --- | --- | --- | --- | --- | --- | --- | --- | --- |
|  | RR | 95% CI | | RR | 95% CI | | RR | 95% CI | |
| **Financial wealth decile** |  |  |  |  |  |  |  |  |  |
| 1 | 3.66 | 3.38 | 3.95 | 3.47 | 3.21 | 3.75 | 2.47 | 2.28 | 2.67 |
| 2 | 3.28 | 3.03 | 3.55 | 3.10 | 2.87 | 3.36 | 2.47 | 2.28 | 2.68 |
| 3 | 2.37 | 2.18 | 2.57 | 2.29 | 2.11 | 2.49 | 2.06 | 1.90 | 2.24 |
| 4 | 1.91 | 1.75 | 2.08 | 1.85 | 1.70 | 2.02 | 1.76 | 1.62 | 1.92 |
| 5 | 1.72 | 1.57 | 1.87 | 1.67 | 1.54 | 1.83 | 1.66 | 1.52 | 1.81 |
| 6 | 1.47 | 1.34 | 1.60 | 1.44 | 1.32 | 1.57 | 1.47 | 1.34 | 1.61 |
| 7 | 1.32 | 1.20 | 1.44 | 1.30 | 1.19 | 1.43 | 1.34 | 1.23 | 1.47 |
| 8 | 1.16 | 1.06 | 1.28 | 1.16 | 1.05 | 1.27 | 1.20 | 1.09 | 1.32 |
| 9 | 1.09 | 0.99 | 1.20 | 1.10 | 1.00 | 1.21 | 1.13 | 1.03 | 1.24 |
| 10 | 1 | 1 | 1 | 1 | 1 | 1 | 1 | 1 | 1 |
| **Sex** |  |  |  |  |  |  |  |  |  |
| Male | 1 | 1 | 1 | 1 | 1 | 1 | 1 | 1 | 1 |
| Female | 0.63 | 0.61 | 0.65 | 0.61 | 0.59 | 0.63 | 0.62 | 0.60 | 0.64 |
| **Age** |  |  |  |  |  |  |  |  |  |
| 25-44 | 1 | 1 | 1 | 1 | 1 | 1 | 1 | 1 | 1 |
| 45-64 | 4.06 | 3.83 | 4.30 | 3.44 | 3.24 | 3.65 | 3.98 | 3.75 | 4.22 |
| 65-79 | 8.94 | 8.44 | 9.47 | 6.58 | 6.20 | 6.97 | 9.48 | 8.91 | 10.09 |
| **Comorbidities** |  |  |  |  |  |  |  |  |  |
| No |  |  |  | 1 | 1 | 1 | 1 | 1 | 1 |
| Yes |  |  |  | 2.32 | 2.24 | 2.41 | 2.36 | 2.27 | 2.45 |
| **Urbanity** |  |  |  |  |  |  |  |  |  |
| 1 |  |  |  |  |  |  | 1 | 1 | 1 |
| 2 |  |  |  |  |  |  | 1.15 | 1.07 | 1.23 |
| 3 |  |  |  |  |  |  | 1.27 | 1.19 | 1.36 |
| 4 |  |  |  |  |  |  | 1.32 | 1.25 | 1.40 |
| 5 |  |  |  |  |  |  | 1.57 | 1.48 | 1.68 |
| 6 |  |  |  |  |  |  | 1.82 | 1.68 | 1.97 |
| **Household size** |  |  |  |  |  |  |  |  |  |
| 1 |  |  |  |  |  |  | 1 | 1 | 1 |
| 2 |  |  |  |  |  |  | 1.35 | 1.29 | 1.41 |
| 3-4 |  |  |  |  |  |  | 1.52 | 1.45 | 1.60 |
| >4 |  |  |  |  |  |  | 1.99 | 1.86 | 2.13 |
| **Country of origin** |  |  |  |  |  |  |  |  |  |
| Dutch |  |  |  |  |  |  | 1 | 1 | 1 |
| Europe |  |  |  |  |  |  | 1.00 | 0.93 | 1.07 |
| Other |  |  |  |  |  |  | 2.89 | 2.78 | 3.01 |

Table A 19 Hospital admissions for COVID-19 by education

|  | Model 1 | | | Model 2 | | | Model 3 | | |
| --- | --- | --- | --- | --- | --- | --- | --- | --- | --- |
|  | RR | 95% CI | | RR | 95% CI | | RR | 95% CI | |
| **Education** |  |  |  |  |  |  |  |  |  |
| Low | 2.96 | 2.78 | 3.15 | 2.75 | 2.58 | 2.93 | 2.11 | 1.98 | 2.25 |
| Middle | 1.69 | 1.59 | 1.80 | 1.61 | 1.51 | 1.72 | 1.60 | 1.50 | 1.70 |
| High | 1 | 1 | 1 | 1 | 1 | 1 | 1 | 1 | 1 |
| **Sex** |  |  |  |  |  |  |  |  |  |
| Male | 1 | 1 | 1 | 1 | 1 | 1 | 1 | 1 | 1 |
| Female | 0.66 | 0.63 | 0.69 | 0.63 | 0.60 | 0.66 | 0.64 | 0.61 | 0.67 |
| **Age** |  |  |  |  |  |  |  |  |  |
| 25-44 | 1 | 1 | 1 | 1 | 1 | 1 | 1 | 1 | 1 |
| 45-64 | 3.74 | 3.50 | 3.99 | 3.23 | 3.02 | 3.45 | 3.72 | 3.48 | 3.98 |
| 65-79 | 6.74 | 6.27 | 7.25 | 5.23 | 4.86 | 5.63 | 8.11 | 7.50 | 8.78 |
| **Comorbidities** |  |  |  |  |  |  |  |  |  |
| No |  |  |  | 1 | 1 | 1 | 1 | 1 | 1 |
| Yes |  |  |  | 2.26 | 2.15 | 2.38 | 2.25 | 2.13 | 2.36 |
| **Urbanity** |  |  |  |  |  |  |  |  |  |
| 1 |  |  |  |  |  |  | 1 | 1 | 1 |
| 2 |  |  |  |  |  |  | 1.26 | 1.13 | 1.41 |
| 3 |  |  |  |  |  |  | 1.39 | 1.25 | 1.54 |
| 4 |  |  |  |  |  |  | 1.50 | 1.37 | 1.65 |
| 5 |  |  |  |  |  |  | 1.81 | 1.63 | 1.99 |
| 6 |  |  |  |  |  |  | 2.16 | 1.93 | 2.43 |
| **Household size** |  |  |  |  |  |  |  |  |  |
| 1 |  |  |  |  |  |  | 1 | 1 | 1 |
| 2 |  |  |  |  |  |  | 1.29 | 1.21 | 1.37 |
| 3-4 |  |  |  |  |  |  | 1.52 | 1.42 | 1.63 |
| >4 |  |  |  |  |  |  | 1.93 | 1.76 | 2.11 |
| **Country of origin** |  |  |  |  |  |  |  |  |  |
| Dutch |  |  |  |  |  |  | 1 | 1 | 1 |
| Europe |  |  |  |  |  |  | 1.08 | 0.97 | 1.21 |
| Other |  |  |  |  |  |  | 3.51 | 3.33 | 3.71 |

Table A 20 Hospital admissions for COVID-19 by imputed education

|  | Model 1 | | | Model 2 | | | Model 3 | | |
| --- | --- | --- | --- | --- | --- | --- | --- | --- | --- |
|  | RR | 95% CI | | RR | 95% CI | | RR | 95% CI | |
| **Imputed education** |  |  |  |  |  |  |  |  |  |
| Low | 2.23 | 2.10 | 2.37 | 2.14 | 2.02 | 2.26 | 1.89 | 1.80 | 1.99 |
| Middle | 1.47 | 1.37 | 1.57 | 1.43 | 1.34 | 1.51 | 1.46 | 1.38 | 1.54 |
| High | 1 | 1 | 1 | 1 | 1 | 1 | 1 | 1 | 1 |
| **Sex** |  |  |  |  |  |  |  |  |  |
| Male | 1 | 1 | 1 | 1 | 1 | 1 | 1 | 1 | 1 |
| Female | 0.59 | 0.57 | 0.61 | 0.57 | 0.55 | 0.59 | 0.59 | 0.57 | 0.61 |
| **Age** |  |  |  |  |  |  |  |  |  |
| 25-44 | 1 | 1 | 1 | 1 | 1 | 1 | 1 | 1 | 1 |
| 45-64 | 3.74 | 3.50 | 3.99 | 3.18 | 2.99 | 3.38 | 3.87 | 3.65 | 4.10 |
| 65-79 | 7.37 | 6.90 | 7.88 | 5.45 | 5.12 | 5.80 | 8.59 | 8.07 | 9.15 |
| **Comorbidities** |  |  |  |  |  |  |  |  |  |
| No |  |  |  | 1 | 1 | 1 | 1 | 1 | 1 |
| Yes |  |  |  | 2.38 | 2.29 | 2.47 | 2.38 | 2.29 | 2.47 |
| **Urbanity** |  |  |  |  |  |  |  |  |  |
| 1 |  |  |  |  |  |  | 1 | 1 | 1 |
| 2 |  |  |  |  |  |  | 1.20 | 1.12 | 1.28 |
| 3 |  |  |  |  |  |  | 1.38 | 1.30 | 1.48 |
| 4 |  |  |  |  |  |  | 1.47 | 1.38 | 1.56 |
| 5 |  |  |  |  |  |  | 1.80 | 1.69 | 1.91 |
| 6 |  |  |  |  |  |  | 2.11 | 1.95 | 2.28 |
| **Household size** |  |  |  |  |  |  |  |  |  |
| 1 |  |  |  |  |  |  | 1 | 1 | 1 |
| 2 |  |  |  |  |  |  | 1.18 | 1.13 | 1.23 |
| 3-4 |  |  |  |  |  |  | 1.32 | 1.26 | 1.39 |
| >4 |  |  |  |  |  |  | 1.71 | 1.60 | 1.83 |
| **Country of origin** |  |  |  |  |  |  |  |  |  |
| Dutch |  |  |  |  |  |  | 1 | 1 | 1 |
| Europe |  |  |  |  |  |  | 1.06 | 0.98 | 1.14 |
| Other |  |  |  |  |  |  | 3.24 | 3.12 | 3.37 |

## ICU admissions for COVID-19 – Income, financial wealth, education and imputed education

Table A 21 ICU admissions for COVID-19 by income decile

|  | Model 1 | | | Model 2 | | | Model 3 | | |
| --- | --- | --- | --- | --- | --- | --- | --- | --- | --- |
|  | RR | 95% CI | | RR | 95% CI | | RR | 95% CI | |
| **Income decile** |  |  |  |  |  |  |  |  |  |
| 1 | 3.78 | 3.17 | 4.51 | 3.70 | 3.10 | 4.41 | 2.46 | 2.05 | 2.95 |
| 2 | 2.40 | 1.99 | 2.89 | 2.34 | 1.94 | 2.82 | 2.02 | 1.67 | 2.44 |
| 3 | 2.02 | 1.67 | 2.45 | 1.99 | 1.64 | 2.41 | 1.90 | 1.56 | 2.30 |
| 4 | 1.65 | 1.35 | 2.01 | 1.63 | 1.34 | 1.99 | 1.63 | 1.34 | 1.99 |
| 5 | 1.75 | 1.44 | 2.13 | 1.74 | 1.43 | 2.11 | 1.77 | 1.45 | 2.15 |
| 6 | 1.60 | 1.31 | 1.95 | 1.59 | 1.30 | 1.94 | 1.63 | 1.34 | 2.00 |
| 7 | 1.38 | 1.12 | 1.69 | 1.37 | 1.11 | 1.68 | 1.41 | 1.15 | 1.74 |
| 8 | 1.26 | 1.02 | 1.55 | 1.25 | 1.02 | 1.55 | 1.29 | 1.05 | 1.60 |
| 9 | 1.25 | 1.02 | 1.54 | 1.25 | 1.02 | 1.54 | 1.28 | 1.04 | 1.58 |
| 10 | 1 | 1 | 1 | 1 | 1 | 1 | 1 | 1 | 1 |
| **Sex** |  |  |  |  |  |  |  |  |  |
| Male | 1 | 1 | 1 | 1 | 1 | 1 | 1 | 1 | 1 |
| Female | 0.39 | 0.36 | 0.43 | 0.39 | 0.36 | 0.42 | 0.40 | 0.36 | 0.43 |
| **Age** |  |  |  |  |  |  |  |  |  |
| 25-44 | 1 | 1 | 1 | 1 | 1 | 1 | 1 | 1 | 1 |
| 45-64 | 6.62 | 5.60 | 7.81 | 5.83 | 4.93 | 6.88 | 6.92 | 5.85 | 8.17 |
| 65-79 | 14.59 | 12.37 | 17.20 | 11.38 | 9.62 | 13.45 | 16.83 | 14.14 | 20.03 |
| **Comorbidities** |  |  |  |  |  |  |  |  |  |
| No |  |  |  | 1 | 1 | 1 | 1 | 1 | 1 |
| Yes |  |  |  | 1.94 | 1.78 | 2.11 | 1.95 | 1.79 | 2.12 |
| **Urbanity** |  |  |  |  |  |  |  |  |  |
| 1 |  |  |  |  |  |  | 1 | 1 | 1 |
| 2 |  |  |  |  |  |  | 1.01 | 0.86 | 1.19 |
| 3 |  |  |  |  |  |  | 1.12 | 0.97 | 1.31 |
| 4 |  |  |  |  |  |  | 1.24 | 1.08 | 1.42 |
| 5 |  |  |  |  |  |  | 1.60 | 1.39 | 1.85 |
| 6 |  |  |  |  |  |  | 1.80 | 1.51 | 2.15 |
| **Household size** |  |  |  |  |  |  |  |  |  |
| 1 |  |  |  |  |  |  | 1 | 1 | 1 |
| 2 |  |  |  |  |  |  | 1.51 | 1.36 | 1.68 |
| 3-4 |  |  |  |  |  |  | 1.59 | 1.41 | 1.80 |
| >4 |  |  |  |  |  |  | 1.86 | 1.57 | 2.21 |
| **Country of origin** |  |  |  |  |  |  |  |  |  |
| Dutch |  |  |  |  |  |  | 1 | 1 | 1 |
| Europe |  |  |  |  |  |  | 1.05 | 0.88 | 1.25 |
| Other |  |  |  |  |  |  | 3.74 | 3.41 | 4.10 |

Table A 1 ICU admissions for COVID-19 by lagged income decile

|  | Model 1 | | | Model 2 | | | Model 3 | | |
| --- | --- | --- | --- | --- | --- | --- | --- | --- | --- |
|  | RR | 95% CI | | RR | 95% CI | | RR | 95% CI | |
| **Lagged income decile** |  |  |  |  |  |  |  |  |  |
| 1 | 3.92 | 3.26 | 4.71 | 3.85 | 3.20 | 4.62 | 2.54 | 2.10 | 3.07 |
| 2 | 2.51 | 2.06 | 3.05 | 2.46 | 2.03 | 2.99 | 2.13 | 1.75 | 2.60 |
| 3 | 2.04 | 1.67 | 2.50 | 2.02 | 1.65 | 2.47 | 1.93 | 1.57 | 2.36 |
| 4 | 2.04 | 1.67 | 2.50 | 2.03 | 1.66 | 2.48 | 2.03 | 1.66 | 2.48 |
| 5 | 1.67 | 1.36 | 2.06 | 1.66 | 1.35 | 2.05 | 1.70 | 1.38 | 2.09 |
| 6 | 1.65 | 1.34 | 2.03 | 1.64 | 1.33 | 2.02 | 1.69 | 1.37 | 2.08 |
| 7 | 1.47 | 1.18 | 1.82 | 1.46 | 1.18 | 1.81 | 1.51 | 1.22 | 1.87 |
| 8 | 1.30 | 1.04 | 1.63 | 1.30 | 1.04 | 1.62 | 1.34 | 1.07 | 1.67 |
| 9 | 1.29 | 1.03 | 1.62 | 1.29 | 1.03 | 1.62 | 1.33 | 1.06 | 1.66 |
| 10 | 1 | 1 | 1 | 1 | 1 | 1 | 1 | 1 | 1 |
| **Sex** |  |  |  |  |  |  |  |  |  |
| Male | 1 | 1 | 1 | 1 | 1 | 1 | 1 | 1 | 1 |
| Female | 0.39 | 0.36 | 0.43 | 0.38 | 0.35 | 0.42 | 0.39 | 0.36 | 0.43 |
| **Age** |  |  |  |  |  |  |  |  |  |
| 25-44 | 1 | 1 | 1 | 1 | 1 | 1 | 1 | 1 | 1 |
| 45-64 | 6.67 | 5.65 | 7.87 | 5.87 | 4.97 | 6.94 | 6.95 | 5.88 | 8.22 |
| 65-79 | 14.69 | 12.46 | 17.32 | 11.46 | 9.70 | 13.55 | 16.92 | 14.21 | 20.14 |
| **Comorbidities** |  |  |  |  |  |  |  |  |  |
| No |  |  |  | 1 | 1 | 1 | 1 | 1 | 1 |
| Yes |  |  |  | 1.95 | 1.79 | 2.12 | 1.95 | 1.79 | 2.12 |
| **Urbanity** |  |  |  |  |  |  |  |  |  |
| 1 |  |  |  |  |  |  | 1 | 1 | 1 |
| 2 |  |  |  |  |  |  | 1.01 | 0.86 | 1.18 |
| 3 |  |  |  |  |  |  | 1.12 | 0.96 | 1.30 |
| 4 |  |  |  |  |  |  | 1.23 | 1.07 | 1.42 |
| 5 |  |  |  |  |  |  | 1.60 | 1.39 | 1.85 |
| 6 |  |  |  |  |  |  | 1.80 | 1.51 | 2.15 |
| **Household size** |  |  |  |  |  |  |  |  |  |
| 1 |  |  |  |  |  |  | 1 | 1 | 1 |
| 2 |  |  |  |  |  |  | 1.51 | 1.35 | 1.67 |
| 3-4 |  |  |  |  |  |  | 1.55 | 1.37 | 1.76 |
| >4 |  |  |  |  |  |  | 1.79 | 1.51 | 2.12 |
| **Country of origin** |  |  |  |  |  |  |  |  |  |
| Dutch |  |  |  |  |  |  | 1 | 1 | 1 |
| Europe |  |  |  |  |  |  | 1.05 | 0.88 | 1.25 |
| Other |  |  |  |  |  |  | 3.76 | 3.43 | 4.12 |

Table A 22 ICU admissions for COVID-19 by financial wealth decile

|  | Model 1 | | | Model 2 | | | Model 3 | | |
| --- | --- | --- | --- | --- | --- | --- | --- | --- | --- |
|  | RR | 95% CI | | RR | 95% CI | | RR | 95% CI | |
| **Financial wealth decile** |  |  |  |  |  |  |  |  |  |
| 1 | 4.42 | 3.67 | 5.33 | 4.25 | 3.52 | 5.12 | 2.89 | 2.38 | 3.50 |
| 2 | 3.55 | 2.93 | 4.30 | 3.41 | 2.81 | 4.13 | 2.62 | 2.15 | 3.19 |
| 3 | 2.62 | 2.15 | 3.20 | 2.55 | 2.09 | 3.12 | 2.27 | 1.85 | 2.77 |
| 4 | 2.05 | 1.67 | 2.52 | 2.01 | 1.64 | 2.47 | 1.90 | 1.54 | 2.33 |
| 5 | 1.86 | 1.51 | 2.30 | 1.83 | 1.48 | 2.25 | 1.81 | 1.47 | 2.23 |
| 6 | 1.51 | 1.22 | 1.88 | 1.49 | 1.20 | 1.85 | 1.52 | 1.22 | 1.89 |
| 7 | 1.22 | 0.97 | 1.53 | 1.21 | 0.96 | 1.52 | 1.25 | 1.00 | 1.57 |
| 8 | 1.32 | 1.05 | 1.65 | 1.31 | 1.05 | 1.64 | 1.37 | 1.09 | 1.71 |
| 9 | 1.06 | 0.84 | 1.35 | 1.07 | 0.84 | 1.35 | 1.10 | 0.87 | 1.39 |
| 10 | 1 | 1 | 1 | 1 | 1 | 1 | 1 | 1 | 1 |
| **Sex** |  |  |  |  |  |  |  |  |  |
| Male | 1 | 1 | 1 | 1 | 1 | 1 | 1 | 1 | 1 |
| Female | 0.40 | 0.37 | 0.43 | 0.39 | 0.36 | 0.42 | 0.40 | 0.37 | 0.43 |
| **Age** |  |  |  |  |  |  |  |  |  |
| 25-44 | 1 | 1 | 1 | 1 | 1 | 1 | 1 | 1 | 1 |
| 45-64 | 6.64 | 5.62 | 7.83 | 5.85 | 4.95 | 6.91 | 6.77 | 5.73 | 8.00 |
| 65-79 | 14.72 | 12.48 | 17.35 | 11.57 | 9.79 | 13.68 | 16.29 | 13.70 | 19.38 |
| **Comorbidities** |  |  |  |  |  |  |  |  |  |
| No |  |  |  | 1 | 1 | 1 | 1 | 1 | 1 |
| Yes |  |  |  | 1.88 | 1.73 | 2.04 | 1.91 | 1.76 | 2.08 |
| **Urbanity** |  |  |  |  |  |  |  |  |  |
| 1 |  |  |  |  |  |  | 1 | 1 | 1 |
| 2 |  |  |  |  |  |  | 0.98 | 0.83 | 1.15 |
| 3 |  |  |  |  |  |  | 1.05 | 0.90 | 1.22 |
| 4 |  |  |  |  |  |  | 1.13 | 0.99 | 1.30 |
| 5 |  |  |  |  |  |  | 1.46 | 1.26 | 1.69 |
| 6 |  |  |  |  |  |  | 1.66 | 1.39 | 1.98 |
| **Household size** |  |  |  |  |  |  |  |  |  |
| 1 |  |  |  |  |  |  | 1 | 1 | 1 |
| 2 |  |  |  |  |  |  | 1.59 | 1.43 | 1.77 |
| 3-4 |  |  |  |  |  |  | 1.63 | 1.44 | 1.84 |
| >4 |  |  |  |  |  |  | 1.88 | 1.59 | 2.23 |
| **Country of origin** |  |  |  |  |  |  |  |  |  |
| Dutch |  |  |  |  |  |  | 1 | 1 | 1 |
| Europe |  |  |  |  |  |  | 1.01 | 0.85 | 1.21 |
| Other |  |  |  |  |  |  | 3.47 | 3.17 | 3.80 |

Table A 23 ICU admissions for COVID-19 by education

|  | Model 1 | | | Model 2 | | | Model 3 | | |
| --- | --- | --- | --- | --- | --- | --- | --- | --- | --- |
|  | RR | 95% CI | | RR | 95% CI | | RR | 95% CI | |
| **Education** |  |  |  |  |  |  |  |  |  |
| Low | 3.44 | 2.96 | 4.00 | 3.26 | 2.80 | 3.79 | 2.42 | 2.08 | 2.82 |
| Middle | 1.76 | 1.51 | 2.06 | 1.71 | 1.46 | 2.00 | 1.68 | 1.44 | 1.97 |
| High | 1 | 1 | 1 | 1 | 1 | 1 | 1 | 1 | 1 |
| **Sex** |  |  |  |  |  |  |  |  |  |
| Male | 1 | 1 | 1 | 1 | 1 | 1 | 1 | 1 | 1 |
| Female | 0.43 | 0.38 | 0.48 | 0.42 | 0.37 | 0.47 | 0.43 | 0.38 | 0.48 |
| **Age** |  |  |  |  |  |  |  |  |  |
| 25-44 | 1 | 1 | 1 | 1 | 1 | 1 | 1 | 1 | 1 |
| 45-64 | 6.00 | 4.98 | 7.23 | 5.37 | 4.45 | 6.47 | 6.17 | 5.11 | 7.44 |
| 65-79 | 12.16 | 10.00 | 14.80 | 9.97 | 8.17 | 12.17 | 15.24 | 12.38 | 18.77 |
| **Comorbidities** |  |  |  |  |  |  |  |  |  |
| No |  |  |  | 1 | 1 | 1 | 1 | 1 | 1 |
| Yes |  |  |  | 1.84 | 1.64 | 2.07 | 1.83 | 1.63 | 2.06 |
| **Urbanity** |  |  |  |  |  |  |  |  |  |
| 1 |  |  |  |  |  |  | 1 | 1 | 1 |
| 2 |  |  |  |  |  |  | 1.08 | 0.84 | 1.38 |
| 3 |  |  |  |  |  |  | 1.05 | 0.83 | 1.33 |
| 4 |  |  |  |  |  |  | 1.29 | 1.04 | 1.60 |
| 5 |  |  |  |  |  |  | 1.64 | 1.31 | 2.05 |
| 6 |  |  |  |  |  |  | 2.01 | 1.56 | 2.60 |
| **Household size** |  |  |  |  |  |  |  |  |  |
| 1 |  |  |  |  |  |  | 1 | 1 | 1 |
| 2 |  |  |  |  |  |  | 1.47 | 1.26 | 1.71 |
| 3-4 |  |  |  |  |  |  | 1.62 | 1.37 | 1.90 |
| >4 |  |  |  |  |  |  | 1.75 | 1.40 | 2.17 |
| **Country of origin** |  |  |  |  |  |  |  |  |  |
| Dutch |  |  |  |  |  |  | 1 | 1 | 1 |
| Europe |  |  |  |  |  |  | 1.15 | 0.87 | 1.50 |
| Other |  |  |  |  |  |  | 4.22 | 3.72 | 4.78 |

Table A 24 ICU admissions for COVID-19 by imputed education

|  | Model 1 | | | Model 2 | | | Model 3 | | |
| --- | --- | --- | --- | --- | --- | --- | --- | --- | --- |
|  | RR | 95% CI | | RR | 95% CI | | RR | 95% CI | |
| **Imputed education** |  |  |  |  |  |  |  |  |  |
| Low | 2.41 | 2.10 | 2.77 | 2.34 | 2.06 | 2.65 | 2.01 | 1.79 | 2.27 |
| Middle | 1.49 | 1.29 | 1.72 | 1.46 | 1.28 | 1.66 | 1.49 | 1.32 | 1.69 |
| High | 1 | 1 | 1 | 1 | 1 | 1 | 1 | 1 | 1 |
| **Sex** |  |  |  |  |  |  |  |  |  |
| Male | 1 | 1 | 1 | 1 | 1 | 1 | 1 | 1 | 1 |
| Female | 0.37 | 0.34 | 0.41 | 0.37 | 0.33 | 0.40 | 0.38 | 0.35 | 0.41 |
| **Age** |  |  |  |  |  |  |  |  |  |
| 25-44 | 1 | 1 | 1 | 1 | 1 | 1 | 1 | 1 | 1 |
| 45-64 | 6.10 | 5.05 | 7.36 | 5.38 | 4.52 | 6.41 | 6.61 | 5.59 | 7.81 |
| 65-79 | 12.03 | 9.96 | 14.53 | 9.46 | 7.92 | 11.29 | 14.92 | 12.52 | 17.79 |
| **Comorbidities** |  |  |  |  |  |  |  |  |  |
| No |  |  |  | 1 | 1 | 1 | 1 | 1 | 1 |
| Yes |  |  |  | 1.93 | 1.76 | 2.10 | 1.93 | 1.78 | 2.10 |
| **Urbanity** |  |  |  |  |  |  |  |  |  |
| 1 |  |  |  |  |  |  | 1 | 1 | 1 |
| 2 |  |  |  |  |  |  | 1.02 | 0.87 | 1.20 |
| 3 |  |  |  |  |  |  | 1.15 | 0.99 | 1.34 |
| 4 |  |  |  |  |  |  | 1.27 | 1.11 | 1.46 |
| 5 |  |  |  |  |  |  | 1.69 | 1.46 | 1.95 |
| 6 |  |  |  |  |  |  | 1.94 | 1.62 | 2.32 |
| **Household size** |  |  |  |  |  |  |  |  |  |
| 1 |  |  |  |  |  |  | 1 | 1 | 1 |
| 2 |  |  |  |  |  |  | 1.38 | 1.24 | 1.53 |
| 3-4 |  |  |  |  |  |  | 1.41 | 1.25 | 1.59 |
| >4 |  |  |  |  |  |  | 1.62 | 1.37 | 1.92 |
| **Country of origin** |  |  |  |  |  |  |  |  |  |
| Dutch |  |  |  |  |  |  | 1 | 1 | 1 |
| Europe |  |  |  |  |  |  | 1.09 | 0.91 | 1.30 |
| Other |  |  |  |  |  |  | 3.98 | 3.64 | 4.35 |

## COVID-19 Mortality – Income, financial wealth, education and imputed education

Table A 25 COVID-19 mortality by income decile

|  | Model 1 | | | Model 2 | | | Model 3 | | |
| --- | --- | --- | --- | --- | --- | --- | --- | --- | --- |
|  | RR | 95% CI | | RR | 95% CI | | RR | 95% CI | |
| **Income decile** |  |  |  |  |  |  |  |  |  |
| 1 | 4.84 | 4.05 | 5.77 | 4.75 | 3.98 | 5.67 | 3.85 | 3.21 | 4.62 |
| 2 | 3.07 | 2.55 | 3.70 | 2.99 | 2.48 | 3.60 | 2.78 | 2.31 | 3.36 |
| 3 | 2.44 | 2.01 | 2.96 | 2.39 | 1.97 | 2.90 | 2.30 | 1.90 | 2.79 |
| 4 | 2.06 | 1.69 | 2.51 | 2.03 | 1.66 | 2.47 | 2.01 | 1.65 | 2.45 |
| 5 | 1.83 | 1.50 | 2.24 | 1.81 | 1.48 | 2.22 | 1.83 | 1.49 | 2.23 |
| 6 | 1.85 | 1.52 | 2.26 | 1.84 | 1.51 | 2.25 | 1.86 | 1.52 | 2.27 |
| 7 | 1.57 | 1.28 | 1.94 | 1.57 | 1.28 | 1.93 | 1.58 | 1.29 | 1.95 |
| 8 | 1.34 | 1.08 | 1.65 | 1.34 | 1.08 | 1.65 | 1.35 | 1.09 | 1.67 |
| 9 | 0.94 | 0.75 | 1.19 | 0.95 | 0.75 | 1.19 | 0.95 | 0.76 | 1.20 |
| 10 | 1 | 1 | 1 | 1 | 1 | 1 | 1 | 1 | 1 |
| **Sex** |  |  |  |  |  |  |  |  |  |
| Male | 1 | 1 | 1 | 1 | 1 | 1 | 1 | 1 | 1 |
| Female | 0.43 | 0.40 | 0.46 | 0.42 | 0.39 | 0.46 | 0.42 | 0.38 | 0.45 |
| **Age** |  |  |  |  |  |  |  |  |  |
| 25-64 | 1 | 1 | 1 | 1 | 1 | 1 | 1 | 1 | 1 |
| 65-79 | 18.31 | 16.61 | 20.19 | 13.53 | 12.25 | 14.95 | 14.68 | 13.14 | 16.41 |
| **Comorbidities** |  |  |  |  |  |  |  |  |  |
| No |  |  |  | 1 | 1 | 1 | 1 | 1 | 1 |
| Yes |  |  |  | 3.44 | 3.11 | 3.81 | 3.47 | 3.14 | 3.84 |
| **Urbanity** |  |  |  |  |  |  |  |  |  |
| 1 |  |  |  |  |  |  | 1 | 1 | 1 |
| 2 |  |  |  |  |  |  | 1.15 | 1.00 | 1.33 |
| 3 |  |  |  |  |  |  | 1.28 | 1.11 | 1.47 |
| 4 |  |  |  |  |  |  | 1.42 | 1.25 | 1.62 |
| 5 |  |  |  |  |  |  | 1.59 | 1.38 | 1.82 |
| 6 |  |  |  |  |  |  | 1.46 | 1.21 | 1.76 |
| **Household size** |  |  |  |  |  |  |  |  |  |
| 1 |  |  |  |  |  |  | 1 | 1 | 1 |
| 2 |  |  |  |  |  |  | 0.93 | 0.85 | 1.01 |
| 3-4 |  |  |  |  |  |  | 0.85 | 0.74 | 0.98 |
| >4 |  |  |  |  |  |  | 1.12 | 0.89 | 1.41 |
| **Country of origin** |  |  |  |  |  |  |  |  |  |
| Dutch |  |  |  |  |  |  | 1 | 1 | 1 |
| Europe |  |  |  |  |  |  | 1.00 | 0.86 | 1.16 |
| Other |  |  |  |  |  |  | 1.70 | 1.54 | 1.89 |

Table A 1 COVID-19 mortality by lagged income decile

|  | Model 1 | | | Model 2 | | | Model 3 | | |
| --- | --- | --- | --- | --- | --- | --- | --- | --- | --- |
|  | RR | 95% CI | | RR | 95% CI | | RR | 95% CI | |
| **Lagged income decile** |  |  |  |  |  |  |  |  |  |
| 1 | 4.00 | 3.32 | 4.82 | 3.96 | 3.28 | 4.78 | 3.10 | 2.56 | 3.76 |
| 2 | 3.49 | 2.88 | 4.23 | 3.42 | 2.83 | 4.14 | 3.16 | 2.61 | 3.83 |
| 3 | 2.79 | 2.29 | 3.41 | 2.74 | 2.25 | 3.35 | 2.63 | 2.16 | 3.21 |
| 4 | 2.47 | 2.02 | 3.02 | 2.45 | 2.00 | 3.00 | 2.43 | 1.99 | 2.98 |
| 5 | 2.01 | 1.64 | 2.48 | 2.00 | 1.63 | 2.46 | 2.01 | 1.63 | 2.48 |
| 6 | 1.93 | 1.57 | 2.38 | 1.92 | 1.56 | 2.37 | 1.94 | 1.57 | 2.39 |
| 7 | 1.83 | 1.48 | 2.26 | 1.83 | 1.48 | 2.26 | 1.84 | 1.49 | 2.27 |
| 8 | 1.39 | 1.11 | 1.74 | 1.39 | 1.11 | 1.74 | 1.40 | 1.12 | 1.75 |
| 9 | 1.06 | 0.84 | 1.35 | 1.07 | 0.84 | 1.35 | 1.07 | 0.84 | 1.36 |
| 10 | 1 | 1 | 1 | 1 | 1 | 1 | 1 | 1 | 1 |
| **Sex** |  |  |  |  |  |  |  |  |  |
| Male | 1 | 1 | 1 | 1 | 1 | 1 | 1 | 1 | 1 |
| Female | 0.42 | 0.39 | 0.46 | 0.42 | 0.39 | 0.45 | 0.41 | 0.38 | 0.45 |
| **Age** |  |  |  |  |  |  |  |  |  |
| 25-64 | 1 | 1 | 1 | 1 | 1 | 1 | 1 | 1 | 1 |
| 65-79 | 18.58 | 16.85 | 20.49 | 13.68 | 12.39 | 15.12 | 14.82 | 13.26 | 16.56 |
| **Comorbidities** |  |  |  |  |  |  |  |  |  |
| No |  |  |  | 1 | 1 | 1 | 1 | 1 | 1 |
| Yes |  |  |  | 3.46 | 3.13 | 3.83 | 3.49 | 3.16 | 3.86 |
| **Urbanity** |  |  |  |  |  |  |  |  |  |
| 1 |  |  |  |  |  |  | 1 | 1 | 1 |
| 2 |  |  |  |  |  |  | 1.15 | 0.99 | 1.32 |
| 3 |  |  |  |  |  |  | 1.27 | 1.11 | 1.46 |
| 4 |  |  |  |  |  |  | 1.42 | 1.25 | 1.61 |
| 5 |  |  |  |  |  |  | 1.61 | 1.40 | 1.84 |
| 6 |  |  |  |  |  |  | 1.52 | 1.26 | 1.83 |
| **Household size** |  |  |  |  |  |  |  |  |  |
| 1 |  |  |  |  |  |  | 1 | 1 | 1 |
| 2 |  |  |  |  |  |  | 0.90 | 0.83 | 0.98 |
| 3-4 |  |  |  |  |  |  | 0.79 | 0.68 | 0.91 |
| >4 |  |  |  |  |  |  | 1.00 | 0.80 | 1.27 |
| **Country of origin** |  |  |  |  |  |  |  |  |  |
| Dutch |  |  |  |  |  |  | 1 | 1 | 1 |
| Europe |  |  |  |  |  |  | 1.03 | 0.89 | 1.20 |
| Other |  |  |  |  |  |  | 1.89 | 1.71 | 2.09 |

Table A 26 COVID-19 mortality by financial wealth decile

|  | Model 1 | | | Model 2 | | | Model 3 | | |
| --- | --- | --- | --- | --- | --- | --- | --- | --- | --- |
|  | RR | 95% CI | | RR | 95% CI | | RR | 95% CI | |
| **Financial wealth decile** |  |  |  |  |  |  |  |  |  |
| 1 | 4.62 | 3.81 | 5.60 | 4.39 | 3.62 | 5.32 | 3.53 | 2.90 | 4.30 |
| 2 | 4.44 | 3.66 | 5.39 | 4.22 | 3.47 | 5.12 | 3.62 | 2.97 | 4.40 |
| 3 | 3.20 | 2.62 | 3.91 | 3.10 | 2.54 | 3.79 | 2.86 | 2.34 | 3.50 |
| 4 | 2.45 | 1.99 | 3.01 | 2.38 | 1.94 | 2.93 | 2.28 | 1.85 | 2.80 |
| 5 | 2.12 | 1.72 | 2.62 | 2.07 | 1.67 | 2.55 | 2.02 | 1.64 | 2.50 |
| 6 | 1.78 | 1.43 | 2.22 | 1.75 | 1.41 | 2.18 | 1.74 | 1.40 | 2.17 |
| 7 | 1.59 | 1.28 | 1.99 | 1.58 | 1.26 | 1.97 | 1.58 | 1.27 | 1.98 |
| 8 | 1.30 | 1.03 | 1.63 | 1.29 | 1.02 | 1.63 | 1.30 | 1.03 | 1.64 |
| 9 | 1.40 | 1.12 | 1.76 | 1.41 | 1.12 | 1.77 | 1.42 | 1.13 | 1.79 |
| 10 | 1 | 1 | 1 | 1 | 1 | 1 | 1 | 1 | 1 |
| **Sex** |  |  |  |  |  |  |  |  |  |
| Male | 1 | 1 | 1 | 1 | 1 | 1 | 1 | 1 | 1 |
| Female | 0.43 | 0.40 | 0.47 | 0.43 | 0.40 | 0.46 | 0.42 | 0.39 | 0.46 |
| **Age** |  |  |  |  |  |  |  |  |  |
| 25-64 | 1 | 1 | 1 | 1 | 1 | 1 | 1 | 1 | 1 |
| 65-79 | 18.42 | 16.71 | 20.31 | 13.65 | 12.35 | 15.08 | 14.19 | 12.73 | 15.83 |
| **Comorbidities** |  |  |  |  |  |  |  |  |  |
| No |  |  |  | 1 | 1 | 1 | 1 | 1 | 1 |
| Yes |  |  |  | 3.36 | 3.04 | 3.72 | 3.41 | 3.08 | 3.77 |
| **Urbanity** |  |  |  |  |  |  |  |  |  |
| 1 |  |  |  |  |  |  | 1 | 1 | 1 |
| 2 |  |  |  |  |  |  | 1.10 | 0.95 | 1.27 |
| 3 |  |  |  |  |  |  | 1.16 | 1.01 | 1.34 |
| 4 |  |  |  |  |  |  | 1.27 | 1.11 | 1.44 |
| 5 |  |  |  |  |  |  | 1.42 | 1.24 | 1.63 |
| 6 |  |  |  |  |  |  | 1.36 | 1.12 | 1.64 |
| **Household size** |  |  |  |  |  |  |  |  |  |
| 1 |  |  |  |  |  |  | 1 | 1 | 1 |
| 2 |  |  |  |  |  |  | 0.98 | 0.90 | 1.06 |
| 3-4 |  |  |  |  |  |  | 0.81 | 0.70 | 0.93 |
| >4 |  |  |  |  |  |  | 1.04 | 0.83 | 1.31 |
| **Country of origin** |  |  |  |  |  |  |  |  |  |
| Dutch |  |  |  |  |  |  | 1 | 1 | 1 |
| Europe |  |  |  |  |  |  | 1.01 | 0.87 | 1.17 |
| Other |  |  |  |  |  |  | 1.75 | 1.58 | 1.94 |

Table A 27 COVID-19 mortality by education

|  | Model 1 | | | Model 2 | | | Model 3 | | |
| --- | --- | --- | --- | --- | --- | --- | --- | --- | --- |
|  | RR | 95% CI | | RR | 95% CI | | RR | 95% CI | |
| **Education** |  |  |  |  |  |  |  |  |  |
| Low | 3.85 | 3.14 | 4.73 | 3.56 | 2.90 | 4.37 | 3.12 | 2.54 | 3.84 |
| Middle | 1.66 | 1.33 | 2.06 | 1.58 | 1.27 | 1.97 | 1.60 | 1.28 | 1.99 |
| High | 1 | 1 | 1 | 1 | 1 | 1 | 1 | 1 | 1 |
| **Sex** |  |  |  |  |  |  |  |  |  |
| Male | 1 | 1 | 1 | 1 | 1 | 1 | 1 | 1 | 1 |
| Female | 0.41 | 0.35 | 0.47 | 0.39 | 0.34 | 0.46 | 0.39 | 0.34 | 0.45 |
| **Age** |  |  |  |  |  |  |  |  |  |
| 25-64 | 1 | 1 | 1 | 1 | 1 | 1 | 1 | 1 | 1 |
| 65-79 | 14.29 | 12.25 | 16.66 | 10.94 | 9.36 | 12.78 | 11.95 | 10.08 | 14.18 |
| **Comorbidities** |  |  |  |  |  |  |  |  |  |
| No |  |  |  | 1 | 1 | 1 | 1 | 1 | 1 |
| Yes |  |  |  | 3.41 | 2.85 | 4.08 | 3.42 | 2.86 | 4.09 |
| **Urbanity** |  |  |  |  |  |  |  |  |  |
| 1 |  |  |  |  |  |  | 1 | 1 | 1 |
| 2 |  |  |  |  |  |  | 1.53 | 1.14 | 2.06 |
| 3 |  |  |  |  |  |  | 1.28 | 0.95 | 1.73 |
| 4 |  |  |  |  |  |  | 1.64 | 1.25 | 2.15 |
| 5 |  |  |  |  |  |  | 1.87 | 1.40 | 2.49 |
| 6 |  |  |  |  |  |  | 2.07 | 1.47 | 2.93 |
| **Household size** |  |  |  |  |  |  |  |  |  |
| 1 |  |  |  |  |  |  | 1 | 1 | 1 |
| 2 |  |  |  |  |  |  | 0.98 | 0.83 | 1.15 |
| 3-4 |  |  |  |  |  |  | 0.79 | 0.63 | 0.99 |
| >4 |  |  |  |  |  |  | 0.79 | 0.54 | 1.15 |
| **Country of origin** |  |  |  |  |  |  |  |  |  |
| Dutch |  |  |  |  |  |  | 1 | 1 | 1 |
| Europe |  |  |  |  |  |  | 1.03 | 0.76 | 1.40 |
| Other |  |  |  |  |  |  | 2.44 | 2.06 | 2.88 |

Table A 28 COVID-19 mortality by imputed education

|  | Model 1 | | | Model 2 | | | Model 3 | | |
| --- | --- | --- | --- | --- | --- | --- | --- | --- | --- |
|  | RR | 95% CI | | RR | 95% CI | | RR | 95% CI | |
| **Imputed education** |  |  |  |  |  |  |  |  |  |
| Low | 2.74 | 2.32 | 3.24 | 2.62 | 2.29 | 3.00 | 2.54 | 2.25 | 2.87 |
| Middle | 1.61 | 1.29 | 2.01 | 1.57 | 1.33 | 1.86 | 1.61 | 1.39 | 1.86 |
| High | 1 | 1 | 1 | 1 | 1 | 1 | 1 | 1 | 1 |
| **Sex** |  |  |  |  |  |  |  |  |  |
| Male | 1 | 1 | 1 | 1 | 1 | 1 | 1 | 1 | 1 |
| Female | 0.39 | 0.36 | 0.43 | 0.39 | 0.36 | 0.42 | 0.38 | 0.35 | 0.41 |
| **Age** |  |  |  |  |  |  |  |  |  |
| 25-64 | 1 | 1 | 1 | 1 | 1 | 1 | 1 | 1 | 1 |
| 65-79 | 15.46 | 13.62 | 17.54 | 11.56 | 10.37 | 12.89 | 12.58 | 11.26 | 14.04 |
| **Comorbidities** |  |  |  |  |  |  |  |  |  |
| No |  |  |  | 1 | 1 | 1 | 1 | 1 | 1 |
| Yes |  |  |  | 3.42 | 3.06 | 3.81 | 3.45 | 3.12 | 3.81 |
| **Urbanity** |  |  |  |  |  |  |  |  |  |
| 1 |  |  |  |  |  |  | 1 | 1 | 1 |
| 2 |  |  |  |  |  |  | 1.16 | 1.01 | 1.34 |
| 3 |  |  |  |  |  |  | 1.31 | 1.14 | 1.51 |
| 4 |  |  |  |  |  |  | 1.48 | 1.30 | 1.68 |
| 5 |  |  |  |  |  |  | 1.73 | 1.51 | 1.99 |
| 6 |  |  |  |  |  |  | 1.70 | 1.41 | 2.06 |
| **Household size** |  |  |  |  |  |  |  |  |  |
| 1 |  |  |  |  |  |  | 1 | 1 | 1 |
| 2 |  |  |  |  |  |  | 0.81 | 0.75 | 0.88 |
| 3-4 |  |  |  |  |  |  | 0.67 | 0.58 | 0.78 |
| >4 |  |  |  |  |  |  | 0.85 | 0.67 | 1.06 |
| **Country of origin** |  |  |  |  |  |  |  |  |  |
| Dutch |  |  |  |  |  |  | 1 | 1 | 1 |
| Europe |  |  |  |  |  |  | 1.08 | 0.93 | 1.25 |
| Other |  |  |  |  |  |  | 2.03 | 1.84 | 2.24 |

## COVID-19 Mortality among hospitalized patients – Income, financial wealth, education and imputed education

Table A 29 COVID-19 mortality among hospitalized by income decile

|  | Model 1 | | | Model 2 | | | Model 3 | | |
| --- | --- | --- | --- | --- | --- | --- | --- | --- | --- |
|  | RR | 95% CI | | RR | 95% CI | | RR | 95% CI | |
| **Income decile** |  |  |  |  |  |  |  |  |  |
| 1 | 1.66 | 1.34 | 2.06 | 1.63 | 1.32 | 2.02 | 1.81 | 1.45 | 2.25 |
| 2 | 1.64 | 1.31 | 2.04 | 1.59 | 1.27 | 1.99 | 1.59 | 1.27 | 1.99 |
| 3 | 1.46 | 1.16 | 1.84 | 1.43 | 1.13 | 1.80 | 1.40 | 1.11 | 1.76 |
| 4 | 1.45 | 1.14 | 1.83 | 1.42 | 1.12 | 1.79 | 1.37 | 1.08 | 1.73 |
| 5 | 1.39 | 1.09 | 1.76 | 1.36 | 1.07 | 1.72 | 1.34 | 1.05 | 1.70 |
| 6 | 1.36 | 1.07 | 1.73 | 1.33 | 1.05 | 1.70 | 1.33 | 1.04 | 1.69 |
| 7 | 1.38 | 1.08 | 1.77 | 1.37 | 1.07 | 1.76 | 1.38 | 1.08 | 1.77 |
| 8 | 1.29 | 1.00 | 1.67 | 1.27 | 0.98 | 1.65 | 1.29 | 0.99 | 1.66 |
| 9 | 0.97 | 0.73 | 1.28 | 0.97 | 0.73 | 1.28 | 0.99 | 0.74 | 1.31 |
| 10 | 1 | 1 | 1 | 1 | 1 | 1 | 1 | 1 | 1 |
| **Sex** |  |  |  |  |  |  |  |  |  |
| Male | 1 | 1 | 1 | 1 | 1 | 1 | 1 | 1 | 1 |
| Female | 0.67 | 0.61 | 0.73 | 0.66 | 0.60 | 0.72 | 0.63 | 0.58 | 0.70 |
| **Age** |  |  |  |  |  |  |  |  |  |
| 25-64 | 1 | 1 | 1 | 1 | 1 | 1 | 1 | 1 | 1 |
| 65-79 | 5.06 | 4.49 | 5.69 | 4.75 | 4.22 | 5.36 | 4.09 | 3.59 | 4.65 |
| **Comorbidities** |  |  |  |  |  |  |  |  |  |
| No |  |  |  | 1 | 1 | 1 | 1 | 1 | 1 |
| Yes |  |  |  | 1.43 | 1.27 | 1.61 | 1.39 | 1.24 | 1.57 |
| **Urbanity** |  |  |  |  |  |  |  |  |  |
| 1 |  |  |  |  |  |  | 1 | 1 | 1 |
| 2 |  |  |  |  |  |  | 0.90 | 0.77 | 1.06 |
| 3 |  |  |  |  |  |  | 0.90 | 0.78 | 1.05 |
| 4 |  |  |  |  |  |  | 0.90 | 0.78 | 1.04 |
| 5 |  |  |  |  |  |  | 0.92 | 0.79 | 1.07 |
| 6 |  |  |  |  |  |  | 0.77 | 0.62 | 0.95 |
| **Household size** |  |  |  |  |  |  |  |  |  |
| 1 |  |  |  |  |  |  | 1 | 1 | 1 |
| 2 |  |  |  |  |  |  | 0.82 | 0.74 | 0.90 |
| 3-4 |  |  |  |  |  |  | 0.68 | 0.58 | 0.81 |
| >4 |  |  |  |  |  |  | 0.64 | 0.48 | 0.84 |
| **Country of origin** |  |  |  |  |  |  |  |  |  |
| Dutch |  |  |  |  |  |  | 1 | 1 | 1 |
| Europe |  |  |  |  |  |  | 0.92 | 0.77 | 1.11 |
| Other |  |  |  |  |  |  | 0.77 | 0.68 | 0.87 |

Table A 1 COVID-19 mortality among hospitalized by lagged income decile

|  | Model 1 | | | Model 2 | | | Model 3 | | |
| --- | --- | --- | --- | --- | --- | --- | --- | --- | --- |
|  | RR | 95% CI | | RR | 95% CI | | RR | 95% CI | |
| **Lagged income decile** |  |  |  |  |  |  |  |  |  |
| 1 | 1.57 | 1.24 | 1.99 | 1.55 | 1.22 | 1.95 | 1.62 | 1.27 | 2.06 |
| 2 | 1.87 | 1.47 | 2.37 | 1.81 | 1.43 | 2.30 | 1.78 | 1.40 | 2.27 |
| 3 | 1.66 | 1.29 | 2.13 | 1.61 | 1.26 | 2.07 | 1.57 | 1.22 | 2.01 |
| 4 | 1.81 | 1.41 | 2.32 | 1.77 | 1.39 | 2.27 | 1.73 | 1.35 | 2.22 |
| 5 | 1.56 | 1.20 | 2.01 | 1.52 | 1.18 | 1.97 | 1.50 | 1.16 | 1.94 |
| 6 | 1.51 | 1.16 | 1.95 | 1.48 | 1.14 | 1.92 | 1.46 | 1.13 | 1.89 |
| 7 | 1.78 | 1.38 | 2.31 | 1.76 | 1.36 | 2.28 | 1.78 | 1.38 | 2.31 |
| 8 | 1.35 | 1.02 | 1.79 | 1.34 | 1.02 | 1.78 | 1.37 | 1.03 | 1.81 |
| 9 | 1.13 | 0.84 | 1.52 | 1.12 | 0.83 | 1.51 | 1.12 | 0.83 | 1.51 |
| 10 | 1 | 1 | 1 | 1 | 1 | 1 | 1 | 1 | 1 |
| **Sex** |  |  |  |  |  |  |  |  |  |
| Male | 1 | 1 | 1 | 1 | 1 | 1 | 1 | 1 | 1 |
| Female | 0.67 | 0.61 | 0.74 | 0.66 | 0.61 | 0.73 | 0.64 | 0.58 | 0.70 |
| **Age** |  |  |  |  |  |  |  |  |  |
| 25-64 | 1 | 1 | 1 | 1 | 1 | 1 | 1 | 1 | 1 |
| 65-79 | 5.15 | 4.57 | 5.79 | 4.83 | 4.28 | 5.45 | 4.14 | 3.63 | 4.71 |
| **Comorbidities** |  |  |  |  |  |  |  |  |  |
| No |  |  |  | 1 | 1 | 1 | 1 | 1 | 1 |
| Yes |  |  |  | 1.43 | 1.27 | 1.61 | 1.40 | 1.24 | 1.57 |
| **Urbanity** |  |  |  |  |  |  |  |  |  |
| 1 |  |  |  |  |  |  | 1 | 1 | 1 |
| 2 |  |  |  |  |  |  | 0.91 | 0.77 | 1.06 |
| 3 |  |  |  |  |  |  | 0.90 | 0.77 | 1.05 |
| 4 |  |  |  |  |  |  | 0.89 | 0.77 | 1.03 |
| 5 |  |  |  |  |  |  | 0.93 | 0.80 | 1.08 |
| 6 |  |  |  |  |  |  | 0.80 | 0.65 | 0.99 |
| **Household size** |  |  |  |  |  |  |  |  |  |
| 1 |  |  |  |  |  |  | 1 | 1 | 1 |
| 2 |  |  |  |  |  |  | 0.80 | 0.73 | 0.88 |
| 3-4 |  |  |  |  |  |  | 0.64 | 0.54 | 0.75 |
| >4 |  |  |  |  |  |  | 0.59 | 0.45 | 0.77 |
| **Country of origin** |  |  |  |  |  |  |  |  |  |
| Dutch |  |  |  |  |  |  | 1 | 1 | 1 |
| Europe |  |  |  |  |  |  | 0.95 | 0.79 | 1.14 |
| Other |  |  |  |  |  |  | 0.85 | 0.75 | 0.96 |

Table A 30 COVID-19 mortality among hospitalized by financial wealth decile

|  | Model 1 | | | Model 2 | | | Model 3 | | |
| --- | --- | --- | --- | --- | --- | --- | --- | --- | --- |
|  | RR | 95% CI | | RR | 95% CI | | RR | 95% CI | |
| **Financial wealth decile** |  |  |  |  |  |  |  |  |  |
| 1 | 1.65 | 1.29 | 2.12 | 1.63 | 1.27 | 2.09 | 1.77 | 1.37 | 2.29 |
| 2 | 1.84 | 1.43 | 2.37 | 1.79 | 1.39 | 2.30 | 1.85 | 1.43 | 2.38 |
| 3 | 1.73 | 1.33 | 2.25 | 1.69 | 1.30 | 2.19 | 1.71 | 1.32 | 2.22 |
| 4 | 1.78 | 1.37 | 2.32 | 1.77 | 1.36 | 2.30 | 1.82 | 1.40 | 2.37 |
| 5 | 1.75 | 1.34 | 2.30 | 1.73 | 1.32 | 2.26 | 1.77 | 1.35 | 2.32 |
| 6 | 1.68 | 1.27 | 2.21 | 1.65 | 1.25 | 2.18 | 1.66 | 1.26 | 2.19 |
| 7 | 1.53 | 1.15 | 2.04 | 1.53 | 1.15 | 2.03 | 1.54 | 1.16 | 2.04 |
| 8 | 1.43 | 1.06 | 1.92 | 1.41 | 1.04 | 1.90 | 1.43 | 1.06 | 1.92 |
| 9 | 1.45 | 1.08 | 1.95 | 1.46 | 1.09 | 1.96 | 1.47 | 1.09 | 1.97 |
| 10 | 1 | 1 | 1 | 1 | 1 | 1 | 1 | 1 | 1 |
| **Sex** |  |  |  |  |  |  |  |  |  |
| Male | 1 | 1 | 1 | 1 | 1 | 1 | 1 | 1 | 1 |
| Female | 0.68 | 0.62 | 0.74 | 0.67 | 0.61 | 0.74 | 0.64 | 0.59 | 0.71 |
| **Age** |  |  |  |  |  |  |  |  |  |
| 25-64 | 1 | 1 | 1 | 1 | 1 | 1 | 1 | 1 | 1 |
| 65-79 | 5.13 | 4.56 | 5.78 | 4.82 | 4.27 | 5.43 | 4.09 | 3.60 | 4.66 |
| **Comorbidities** |  |  |  |  |  |  |  |  |  |
| No |  |  |  | 1 | 1 | 1 | 1 | 1 | 1 |
| Yes |  |  |  | 1.44 | 1.27 | 1.62 | 1.40 | 1.24 | 1.57 |
| **Urbanity** |  |  |  |  |  |  |  |  |  |
| 1 |  |  |  |  |  |  | 1 | 1 | 1 |
| 2 |  |  |  |  |  |  | 0.90 | 0.76 | 1.05 |
| 3 |  |  |  |  |  |  | 0.87 | 0.75 | 1.02 |
| 4 |  |  |  |  |  |  | 0.87 | 0.75 | 1.00 |
| 5 |  |  |  |  |  |  | 0.90 | 0.77 | 1.05 |
| 6 |  |  |  |  |  |  | 0.78 | 0.63 | 0.96 |
| **Household size** |  |  |  |  |  |  |  |  |  |
| 1 |  |  |  |  |  |  | 1 | 1 | 1 |
| 2 |  |  |  |  |  |  | 0.81 | 0.74 | 0.90 |
| 3-4 |  |  |  |  |  |  | 0.63 | 0.54 | 0.75 |
| >4 |  |  |  |  |  |  | 0.59 | 0.45 | 0.77 |
| **Country of origin** |  |  |  |  |  |  |  |  |  |
| Dutch |  |  |  |  |  |  | 1 | 1 | 1 |
| Europe |  |  |  |  |  |  | 0.94 | 0.78 | 1.12 |
| Other |  |  |  |  |  |  | 0.84 | 0.74 | 0.94 |

Table A 31 COVID-19 mortality among hospitalized by education

|  | Model 1 | | | Model 2 | | | Model 3 | | |
| --- | --- | --- | --- | --- | --- | --- | --- | --- | --- |
|  | RR | 95% CI | | RR | 95% CI | | RR | 95% CI | |
| **Education** |  |  |  |  |  |  |  |  |  |
| Low | 1.50 | 1.17 | 1.92 | 1.44 | 1.13 | 1.85 | 1.48 | 1.15 | 1.90 |
| Middle | 1.29 | 0.99 | 1.68 | 1.26 | 0.97 | 1.64 | 1.25 | 0.96 | 1.63 |
| High | 1 | 1 | 1 | 1 | 1 | 1 | 1 | 1 | 1 |
| **Sex** |  |  |  |  |  |  |  |  |  |
| Male | 1 | 1 | 1 | 1 | 1 | 1 | 1 | 1 | 1 |
| Female | 0.67 | 0.56 | 0.79 | 0.66 | 0.55 | 0.78 | 0.62 | 0.52 | 0.74 |
| **Age** |  |  |  |  |  |  |  |  |  |
| 25-64 | 1 | 1 | 1 | 1 | 1 | 1 | 1 | 1 | 1 |
| 65-79 | 4.65 | 3.89 | 5.55 | 4.39 | 3.67 | 5.25 | 3.67 | 3.03 | 4.45 |
| **Comorbidities** |  |  |  |  |  |  |  |  |  |
| No |  |  |  | 1 | 1 | 1 | 1 | 1 | 1 |
| Yes |  |  |  | 1.47 | 1.19 | 1.81 | 1.40 | 1.14 | 1.73 |
| **Urbanity** |  |  |  |  |  |  |  |  |  |
| 1 |  |  |  |  |  |  | 1 | 1 | 1 |
| 2 |  |  |  |  |  |  | 0.96 | 0.69 | 1.35 |
| 3 |  |  |  |  |  |  | 0.89 | 0.64 | 1.24 |
| 4 |  |  |  |  |  |  | 0.97 | 0.71 | 1.31 |
| 5 |  |  |  |  |  |  | 0.99 | 0.73 | 1.36 |
| 6 |  |  |  |  |  |  | 0.91 | 0.62 | 1.32 |
| **Household size** |  |  |  |  |  |  |  |  |  |
| 1 |  |  |  |  |  |  | 1 | 1 | 1 |
| 2 |  |  |  |  |  |  | 0.83 | 0.69 | 1.00 |
| 3-4 |  |  |  |  |  |  | 0.60 | 0.47 | 0.78 |
| >4 |  |  |  |  |  |  | 0.44 | 0.28 | 0.69 |
| **Country of origin** |  |  |  |  |  |  |  |  |  |
| Dutch |  |  |  |  |  |  | 1 | 1 | 1 |
| Europe |  |  |  |  |  |  | 0.86 | 0.59 | 1.26 |
| Other |  |  |  |  |  |  | 0.89 | 0.74 | 1.07 |

Table A 32 COVID-19 mortality among hospitalized by imputed education

|  | Model 1 | | | Model 2 | | | Model 3 | | |
| --- | --- | --- | --- | --- | --- | --- | --- | --- | --- |
|  | RR | 95% CI | | RR | 95% CI | | RR | 95% CI | |
| **Imputed education** |  |  |  |  |  |  |  |  |  |
| Low | 1.38 | 1.12 | 1.69 | 1.35 | 1.14 | 1.59 | 1.39 | 1.19 | 1.61 |
| Middle | 1.21 | 0.94 | 1.57 | 1.20 | 0.98 | 1.46 | 1.20 | 1.01 | 1.42 |
| High | 1 | 1 | 1 | 1 | 1 | 1 | 1 | 1 | 1 |
| **Sex** |  |  |  |  |  |  |  |  |  |
| Male | 1 | 1 | 1 | 1 | 1 | 1 | 1 | 1 | 1 |
| Female | 0.66 | 0.59 | 0.75 | 0.65 | 0.59 | 0.72 | 0.62 | 0.57 | 0.69 |
| **Age** |  |  |  |  |  |  |  |  |  |
| 25-64 | 1 | 1 | 1 | 1 | 1 | 1 | 1 | 1 | 1 |
| 65-79 | 4.93 | 4.23 | 5.74 | 4.64 | 4.07 | 5.29 | 3.94 | 3.46 | 4.48 |
| **Comorbidities** |  |  |  |  |  |  |  |  |  |
| No |  |  |  | 1 | 1 | 1 | 1 | 1 | 1 |
| Yes |  |  |  | 1.44 | 1.26 | 1.63 | 1.40 | 1.24 | 1.57 |
| **Urbanity** |  |  |  |  |  |  |  |  |  |
| 1 |  |  |  |  |  |  | 1 | 1 | 1 |
| 2 |  |  |  |  |  |  | 0.91 | 0.77 | 1.07 |
| 3 |  |  |  |  |  |  | 0.91 | 0.78 | 1.07 |
| 4 |  |  |  |  |  |  | 0.91 | 0.79 | 1.05 |
| 5 |  |  |  |  |  |  | 0.95 | 0.82 | 1.11 |
| 6 |  |  |  |  |  |  | 0.81 | 0.66 | 1.01 |
| **Household size** |  |  |  |  |  |  |  |  |  |
| 1 |  |  |  |  |  |  | 1 | 1 | 1 |
| 2 |  |  |  |  |  |  | 0.79 | 0.72 | 0.87 |
| 3-4 |  |  |  |  |  |  | 0.61 | 0.52 | 0.72 |
| >4 |  |  |  |  |  |  | 0.56 | 0.42 | 0.73 |
| **Country of origin** |  |  |  |  |  |  |  |  |  |
| Dutch |  |  |  |  |  |  | 1 | 1 | 1 |
| Europe |  |  |  |  |  |  | 0.96 | 0.80 | 1.15 |
| Other |  |  |  |  |  |  | 0.85 | 0.76 | 0.95 |

## COVID-19 Mortality, healthcare utilization and testing – Income, financial wealth, education and imputed education

Table A 33 COVID-19 Mortality, healthcare utilization and testing by income decile

|  | Model 2 | | | Model 4 | | | Model 5 | | | Model 6 | | | Model 7 | | |
| --- | --- | --- | --- | --- | --- | --- | --- | --- | --- | --- | --- | --- | --- | --- | --- |
|  | RR | 95% CI | | RR | 95% CI | | RR | 95% CI | | RR | 95% CI | | RR | 95% CI | |
| **Income decile** |  |  |  |  |  |  |  |  |  |  |  |  |  |  |  |
| 1 | 4.75 | 3.98 | 5.67 | 5.29 | 4.43 | 6.31 | 4.62 | 3.87 | 5.51 | 2.06 | 1.74 | 2.44 | 1.83 | 1.55 | 2.16 |
| 2 | 2.99 | 2.48 | 3.60 | 3.25 | 2.69 | 3.91 | 2.96 | 2.45 | 3.57 | 1.75 | 1.47 | 2.09 | 1.74 | 1.47 | 2.07 |
| 3 | 2.39 | 1.97 | 2.90 | 2.55 | 2.11 | 3.10 | 2.34 | 1.93 | 2.84 | 1.60 | 1.33 | 1.92 | 1.56 | 1.30 | 1.86 |
| 4 | 2.03 | 1.66 | 2.47 | 2.15 | 1.76 | 2.62 | 1.98 | 1.63 | 2.41 | 1.51 | 1.26 | 1.83 | 1.53 | 1.27 | 1.84 |
| 5 | 1.81 | 1.48 | 2.22 | 1.91 | 1.56 | 2.33 | 1.77 | 1.45 | 2.16 | 1.40 | 1.16 | 1.70 | 1.35 | 1.12 | 1.63 |
| 6 | 1.84 | 1.51 | 2.25 | 1.91 | 1.57 | 2.34 | 1.78 | 1.46 | 2.18 | 1.46 | 1.21 | 1.77 | 1.42 | 1.18 | 1.71 |
| 7 | 1.57 | 1.28 | 1.93 | 1.62 | 1.32 | 1.99 | 1.54 | 1.25 | 1.89 | 1.43 | 1.18 | 1.74 | 1.41 | 1.16 | 1.70 |
| 8 | 1.34 | 1.08 | 1.65 | 1.36 | 1.10 | 1.68 | 1.31 | 1.06 | 1.62 | 1.30 | 1.06 | 1.59 | 1.30 | 1.07 | 1.59 |
| 9 | 0.95 | 0.75 | 1.19 | 0.95 | 0.76 | 1.20 | 0.94 | 0.74 | 1.18 | 0.95 | 0.77 | 1.19 | 0.94 | 0.76 | 1.16 |
| 10 | 1 | 1 | 1 | 1 | 1 | 1 | 1 | 1 | 1 | 1 | 1 | 1 | 1 | 1 | 1 |
| **Sex** |  |  |  |  |  |  |  |  |  |  |  |  |  |  |  |
| Male | 1 | 1 | 1 | 1 | 1 | 1 | 1 | 1 | 1 | 1 | 1 | 1 | 1 | 1 | 1 |
| Female | 0.42 | 0.39 | 0.46 | 0.41 | 0.38 | 0.45 | 0.42 | 0.39 | 0.46 | 0.60 | 0.55 | 0.64 | 0.66 | 0.62 | 0.71 |
| **Age** |  |  |  |  |  |  |  |  |  |  |  |  |  |  |  |
| 25-64 | 1 | 1 | 1 | 1 | 1 | 1 | 1 | 1 | 1 | 1 | 1 | 1 | 1 | 1 | 1 |
| 65-79 | 13.53 | 12.25 | 14.95 | 15.85 | 14.33 | 17.54 | 15.62 | 14.13 | 17.28 | 7.41 | 6.69 | 8.19 | 7.27 | 6.57 | 8.04 |
| **Comorbidities** |  |  |  |  |  |  |  |  |  |  |  |  |  |  |  |
| No | 1 | 1 | 1 | 1 | 1 | 1 | 1 | 1 | 1 | 1 | 1 | 1 | 1 | 1 | 1 |
| Yes | 3.44 | 3.11 | 3.81 | 3.29 | 2.98 | 3.64 | 3.34 | 3.02 | 3.70 | 2.10 | 1.90 | 2.32 | 2.11 | 1.92 | 2.33 |
| **Administered COVID-19 test** |  |  |  |  |  |  |  |  |  |  |  |  |  |  |  |
| No |  |  |  | 1 | 1 | 1 | 1 | 1 | 1 | 1 | 1 | 1 | 1 | 1 | 1 |
| Yes |  |  |  | 2.37 | 2.19 | 2.57 | 0.44 | 0.37 | 0.52 | 0.48 | 0.41 | 0.56 | 0.59 | 0.51 | 0.68 |
| **Positive COVID-19 test** |  |  |  |  |  |  |  |  |  |  |  |  |  |  |  |
| No |  |  |  |  |  |  | 1 | 1 | 1 | 1 | 1 | 1 | 1 | 1 | 1 |
| Yes |  |  |  |  |  |  | 30.76 | 25.82 | 36.64 | 1.65 | 1.38 | 1.96 | 1.30 | 1.10 | 1.52 |
| **COVID-19 hospital admission** |  |  |  |  |  |  |  |  |  |  |  |  |  |  |  |
| No |  |  |  |  |  |  |  |  |  | 1 | 1 | 1 | 1 | 1 | 1 |
| Yes |  |  |  |  |  |  |  |  |  | 540.95 | 497.50 | 588.19 | 414.48 | 378.65 | 453.70 |
| **COVID-19 ICU admission** |  |  |  |  |  |  |  |  |  |  |  |  |  |  |  |
| No |  |  |  |  |  |  |  |  |  |  |  |  | 1 | 1 | 1 |
| Yes |  |  |  |  |  |  |  |  |  |  |  |  | 2.77 | 2.56 | 2.99 |

Table A 33 COVID-19 Mortality, healthcare utilization and testing by lagged income decile

|  | Model 2 | | | Model 4 | | | Model 5 | | | Model 6 | | | Model 7 | | |
| --- | --- | --- | --- | --- | --- | --- | --- | --- | --- | --- | --- | --- | --- | --- | --- |
|  | RR | 95% CI | | RR | 95% CI | | RR | 95% CI | | RR | 95% CI | | RR | 95% CI | |
| **Income decile** |  |  |  |  |  |  |  |  |  |  |  |  |  |  |  |
| 1 | 3.96 | 3.28 | 4.78 | 4.33 | 3.59 | 5.23 | 3.84 | 3.19 | 4.64 | 1.75 | 1.46 | 2.09 | 1.65 | 1.38 | 1.97 |
| 2 | 3.42 | 2.83 | 4.14 | 3.70 | 3.05 | 4.48 | 3.39 | 2.80 | 4.10 | 1.99 | 1.66 | 2.39 | 1.85 | 1.54 | 2.21 |
| 3 | 2.74 | 2.25 | 3.35 | 2.92 | 2.39 | 3.56 | 2.68 | 2.20 | 3.27 | 1.76 | 1.46 | 2.13 | 1.73 | 1.44 | 2.09 |
| 4 | 2.45 | 2.00 | 3.00 | 2.58 | 2.11 | 3.16 | 2.38 | 1.95 | 2.91 | 1.74 | 1.44 | 2.11 | 1.64 | 1.36 | 1.98 |
| 5 | 2.00 | 1.63 | 2.46 | 2.09 | 1.70 | 2.58 | 1.95 | 1.58 | 2.40 | 1.54 | 1.26 | 1.88 | 1.50 | 1.24 | 1.83 |
| 6 | 1.92 | 1.56 | 2.37 | 1.99 | 1.62 | 2.46 | 1.88 | 1.52 | 2.31 | 1.52 | 1.24 | 1.85 | 1.47 | 1.21 | 1.79 |
| 7 | 1.83 | 1.48 | 2.26 | 1.87 | 1.52 | 2.32 | 1.79 | 1.45 | 2.21 | 1.68 | 1.37 | 2.05 | 1.63 | 1.34 | 1.98 |
| 8 | 1.39 | 1.11 | 1.74 | 1.41 | 1.13 | 1.77 | 1.37 | 1.09 | 1.71 | 1.32 | 1.06 | 1.63 | 1.30 | 1.05 | 1.60 |
| 9 | 1.07 | 0.84 | 1.35 | 1.07 | 0.84 | 1.36 | 1.06 | 0.83 | 1.35 | 1.07 | 0.85 | 1.34 | 1.06 | 0.85 | 1.32 |
| 10 | 1 | 1 | 1 | 1 | 1 | 1 | 1 | 1 | 1 | 1 | 1 | 1 | 1 | 1 | 1 |
| **Sex** |  |  |  |  |  |  |  |  |  |  |  |  |  |  |  |
| Male | 1 | 1 | 1 | 1 | 1 | 1 | 1 | 1 | 1 | 1 | 1 | 1 | 1 | 1 | 1 |
| Female | 0.42 | 0.39 | 0.45 | 0.41 | 0.38 | 0.44 | 0.42 | 0.39 | 0.46 | 0.60 | 0.56 | 0.64 | 0.67 | 0.62 | 0.72 |
| **Age** |  |  |  |  |  |  |  |  |  |  |  |  |  |  |  |
| 25-64 | 1 | 1 | 1 | 1 | 1 | 1 | 1 | 1 | 1 | 1 | 1 | 1 | 1 | 1 | 1 |
| 65-79 | 13.68 | 12.39 | 15.12 | 16.04 | 14.50 | 17.75 | 15.86 | 14.34 | 17.55 | 7.50 | 6.77 | 8.29 | 7.34 | 6.64 | 8.12 |
| **Comorbidities** |  |  |  |  |  |  |  |  |  |  |  |  |  |  |  |
| No | 1 | 1 | 1 | 1 | 1 | 1 | 1 | 1 | 1 | 1 | 1 | 1 | 1 | 1 | 1 |
| Yes | 3.46 | 3.13 | 3.83 | 3.32 | 3.00 | 3.67 | 3.37 | 3.05 | 3.73 | 2.10 | 1.90 | 2.32 | 2.11 | 1.92 | 2.33 |
| **Administered COVID-19 test** |  |  |  |  |  |  |  |  |  |  |  |  |  |  |  |
| No |  |  |  | 1 | 1 | 1 | 1 | 1 | 1 | 1 | 1 | 1 | 1 | 1 | 1 |
| Yes |  |  |  | 2.32 | 2.14 | 2.51 | 0.43 | 0.36 | 0.51 | 0.47 | 0.40 | 0.56 | 0.57 | 0.49 | 0.67 |
| **Positive COVID-19 test** |  |  |  |  |  |  |  |  |  |  |  |  |  |  |  |
| No |  |  |  |  |  |  | 1 | 1 | 1 | 1 | 1 | 1 | 1 | 1 | 1 |
| Yes |  |  |  |  |  |  | 31.17 | 26.16 | 37.13 | 1.64 | 1.37 | 1.95 | 1.31 | 1.12 | 1.54 |
| **COVID-19 hospital admission** |  |  |  |  |  |  |  |  |  |  |  |  |  |  |  |
| No |  |  |  |  |  |  |  |  |  | 1 | 1 | 1 | 1 | 1 | 1 |
| Yes |  |  |  |  |  |  |  |  |  | 559.70 | 514.86 | 608.45 | 424.36 | 387.73 | 464.47 |
| **COVID-19 ICU admission** |  |  |  |  |  |  |  |  |  |  |  |  |  |  |  |
| No |  |  |  |  |  |  |  |  |  |  |  |  | 1 | 1 | 1 |
| Yes |  |  |  |  |  |  |  |  |  |  |  |  | 2.79 | 2.58 | 3.02 |

Table A 34 COVID-19 Mortality, healthcare utilization and testing by financial wealth decile

|  | Model 2 | | | Model 4 | | | Model 5 | | | Model 6 | | | Model 7 | | |
| --- | --- | --- | --- | --- | --- | --- | --- | --- | --- | --- | --- | --- | --- | --- | --- |
|  | RR | 95% CI | | RR | 95% CI | | RR | 95% CI | | RR | 95% CI | | RR | 95% CI | |
| **Financial wealth decile** |  |  |  |  |  |  |  |  |  |  |  |  |  |  |  |
| 1 | 4.39 | 3.62 | 5.32 | 4.52 | 3.73 | 5.48 | 4.15 | 3.43 | 5.04 | 1.83 | 1.52 | 2.21 | 1.74 | 1.45 | 2.09 |
| 2 | 4.22 | 3.47 | 5.12 | 4.39 | 3.61 | 5.33 | 4.12 | 3.40 | 5.01 | 2.05 | 1.70 | 2.47 | 1.94 | 1.62 | 2.33 |
| 3 | 3.10 | 2.54 | 3.79 | 3.21 | 2.63 | 3.92 | 3.04 | 2.49 | 3.72 | 1.91 | 1.58 | 2.31 | 1.88 | 1.56 | 2.27 |
| 4 | 2.38 | 1.94 | 2.93 | 2.42 | 1.97 | 2.98 | 2.33 | 1.89 | 2.86 | 1.75 | 1.44 | 2.13 | 1.74 | 1.44 | 2.11 |
| 5 | 2.07 | 1.67 | 2.55 | 2.08 | 1.68 | 2.57 | 2.03 | 1.64 | 2.50 | 1.68 | 1.37 | 2.05 | 1.62 | 1.33 | 1.97 |
| 6 | 1.75 | 1.41 | 2.18 | 1.76 | 1.41 | 2.19 | 1.70 | 1.37 | 2.12 | 1.52 | 1.23 | 1.87 | 1.50 | 1.23 | 1.84 |
| 7 | 1.58 | 1.26 | 1.97 | 1.58 | 1.26 | 1.97 | 1.54 | 1.23 | 1.92 | 1.47 | 1.19 | 1.81 | 1.48 | 1.20 | 1.82 |
| 8 | 1.29 | 1.02 | 1.63 | 1.29 | 1.02 | 1.63 | 1.26 | 1.00 | 1.59 | 1.28 | 1.03 | 1.60 | 1.28 | 1.03 | 1.59 |
| 9 | 1.41 | 1.12 | 1.77 | 1.41 | 1.12 | 1.77 | 1.40 | 1.11 | 1.75 | 1.39 | 1.12 | 1.73 | 1.44 | 1.16 | 1.79 |
| 10 | 1 | 1 | 1 | 1 | 1 | 1 | 1 | 1 | 1 | 1 | 1 | 1 | 1 | 1 | 1 |
| **Sex** |  |  |  |  |  |  |  |  |  |  |  |  |  |  |  |
| Male | 1 | 1 | 1 | 1 | 1 | 1 | 1 | 1 | 1 | 1 | 1 | 1 | 1 | 1 | 1 |
| Female | 0.43 | 0.40 | 0.46 | 0.42 | 0.39 | 0.45 | 0.43 | 0.40 | 0.47 | 0.61 | 0.56 | 0.65 | 0.67 | 0.62 | 0.72 |
| **Age** |  |  |  |  |  |  |  |  |  |  |  |  |  |  |  |
| 25-64 | 1 | 1 | 1 | 1 | 1 | 1 | 1 | 1 | 1 | 1 | 1 | 1 | 1 | 1 | 1 |
| 65-79 | 13.65 | 12.35 | 15.08 | 15.89 | 14.36 | 17.58 | 15.75 | 14.24 | 17.42 | 7.45 | 6.73 | 8.24 | 7.29 | 6.60 | 8.07 |
| **Comorbidities** |  |  |  |  |  |  |  |  |  |  |  |  |  |  |  |
| No | 1 | 1 | 1 | 1 | 1 | 1 | 1 | 1 | 1 | 1 | 1 | 1 | 1 | 1 | 1 |
| Yes | 3.36 | 3.04 | 3.72 | 3.23 | 2.92 | 3.57 | 3.28 | 2.97 | 3.63 | 2.09 | 1.89 | 2.30 | 2.11 | 1.91 | 2.32 |
| **Administered COVID-19 test** |  |  |  |  |  |  |  |  |  |  |  |  |  |  |  |
| No |  |  |  | 1 | 1 | 1 | 1 | 1 | 1 | 1 | 1 | 1 | 1 | 1 | 1 |
| Yes |  |  |  | 2.23 | 2.07 | 2.42 | 0.41 | 0.35 | 0.49 | 0.47 | 0.40 | 0.55 | 0.56 | 0.49 | 0.65 |
| **Positive COVID-19 test** |  |  |  |  |  |  |  |  |  |  |  |  |  |  |  |
| No |  |  |  |  |  |  | 1 | 1 | 1 | 1 | 1 | 1 | 1 | 1 | 1 |
| Yes |  |  |  |  |  |  | 31.61 | 26.54 | 37.66 | 1.65 | 1.39 | 1.96 | 1.33 | 1.13 | 1.57 |
| **COVID-19 hospital admission** |  |  |  |  |  |  |  |  |  |  |  |  |  |  |  |
| No |  |  |  |  |  |  |  |  |  | 1 | 1 | 1 | 1 | 1 | 1 |
| Yes |  |  |  |  |  |  |  |  |  | 549.45 | 505.14 | 597.64 | 417.23 | 380.98 | 456.93 |
| **COVID-19 ICU admission** |  |  |  |  |  |  |  |  |  |  |  |  |  |  |  |
| No |  |  |  |  |  |  |  |  |  |  |  |  | 1 | 1 | 1 |
| Yes |  |  |  |  |  |  |  |  |  |  |  |  | 2.80 | 2.59 | 3.03 |

Table A 35 COVID-19 Mortality, healthcare utilization and testing by education

|  | Model 2 | | | Model 4 | | | Model 5 | | | Model 6 | | | Model 7 | | |
| --- | --- | --- | --- | --- | --- | --- | --- | --- | --- | --- | --- | --- | --- | --- | --- |
|  | RR | 95% CI | | RR | 95% CI | | RR | 95% CI | | RR | 95% CI | | RR | 95% CI | |
| **Education** |  |  |  |  |  |  |  |  |  |  |  |  |  |  |  |
| Low | 3.56 | 2.90 | 4.37 | 3.99 | 3.25 | 4.90 | 3.37 | 2.74 | 4.13 | 1.62 | 1.33 | 1.99 | 1.56 | 1.28 | 1.89 |
| Middle | 1.58 | 1.27 | 1.97 | 1.68 | 1.35 | 2.09 | 1.53 | 1.23 | 1.91 | 1.22 | 0.99 | 1.51 | 1.25 | 1.02 | 1.54 |
| High | 1 | 1 | 1 | 1 | 1 | 1 | 1 | 1 | 1 | 1 | 1 | 1 | 1 | 1 | 1 |
| **Sex** |  |  |  |  |  |  |  |  |  |  |  |  |  |  |  |
| Male | 1 | 1 | 1 | 1 | 1 | 1 | 1 | 1 | 1 | 1 | 1 | 1 | 1 | 1 | 1 |
| Female | 0.39 | 0.34 | 0.46 | 0.38 | 0.33 | 0.44 | 0.40 | 0.34 | 0.46 | 0.60 | 0.52 | 0.69 | 0.70 | 0.60 | 0.80 |
| **Age** |  |  |  |  |  |  |  |  |  |  |  |  |  |  |  |
| 25-64 | 1 | 1 | 1 | 1 | 1 | 1 | 1 | 1 | 1 | 1 | 1 | 1 | 1 | 1 | 1 |
| 65-79 | 10.94 | 9.36 | 12.78 | 12.47 | 10.64 | 14.61 | 12.64 | 10.79 | 14.81 | 5.92 | 5.05 | 6.95 | 5.50 | 4.70 | 6.44 |
| **Comorbidities** |  |  |  |  |  |  |  |  |  |  |  |  |  |  |  |
| No | 1 | 1 | 1 | 1 | 1 | 1 | 1 | 1 | 1 | 1 | 1 | 1 | 1 | 1 | 1 |
| Yes | 3.41 | 2.85 | 4.08 | 3.26 | 2.72 | 3.90 | 3.30 | 2.76 | 3.95 | 1.98 | 1.66 | 2.37 | 2.01 | 1.69 | 2.39 |
| **Administered COVID-19 test** |  |  |  |  |  |  |  |  |  |  |  |  |  |  |  |
| No |  |  |  | 1 | 1 | 1 | 1 | 1 | 1 | 1 | 1 | 1 | 1 | 1 | 1 |
| Yes |  |  |  | 2.31 | 1.99 | 2.67 | 0.45 | 0.34 | 0.61 | 0.50 | 0.38 | 0.66 | 0.58 | 0.44 | 0.75 |
| **Positive COVID-19 test** |  |  |  |  |  |  |  |  |  |  |  |  |  |  |  |
| No |  |  |  |  |  |  | 1 | 1 | 1 | 1 | 1 | 1 | 1 | 1 | 1 |
| Yes |  |  |  |  |  |  | 28.50 | 21.08 | 38.54 | 1.42 | 1.05 | 1.92 | 1.23 | 0.93 | 1.63 |
| **COVID-19 hospital admission** |  |  |  |  |  |  |  |  |  |  |  |  |  |  |  |
| No |  |  |  |  |  |  |  |  |  | 1 | 1 | 1 | 1 | 1 | 1 |
| Yes |  |  |  |  |  |  |  |  |  | 952.71 | 802.85 | 1130.56 | 589.86 | 487.52 | 713.68 |
| **COVID-19 ICU admission** |  |  |  |  |  |  |  |  |  |  |  |  |  |  |  |
| No |  |  |  |  |  |  |  |  |  |  |  |  | 1 | 1 | 1 |
| Yes |  |  |  |  |  |  |  |  |  |  |  |  | 4.09 | 3.52 | 4.75 |

Table A 36 COVID-19 Mortality, healthcare utilization and testing by imputed education

|  | Model 2 | | | Model 4 | | | Model 5 | | | Model 6 | | | Model 7 | | |
| --- | --- | --- | --- | --- | --- | --- | --- | --- | --- | --- | --- | --- | --- | --- | --- |
|  | RR | 95% CI | | RR | 95% CI | | RR | 95% CI | | RR | 95% CI | | RR | 95% CI | |
| **Imputed education** |  |  |  |  |  |  |  |  |  |  |  |  |  |  |  |
| Low | 2.62 | 2.29 | 3.00 | 2.82 | 2.48 | 3.20 | 2.52 | 2.22 | 2.85 | 1.55 | 1.37 | 1.76 | 1.50 | 1.33 | 1.69 |
| Middle | 1.57 | 1.33 | 1.86 | 1.64 | 1.40 | 1.91 | 1.53 | 1.32 | 1.78 | 1.27 | 1.09 | 1.47 | 1.27 | 1.10 | 1.46 |
| High | 1 | 1 | 1 | 1 | 1 | 1 | 1 | 1 | 1 | 1 | 1 | 1 | 1 | 1 | 1 |
| **Sex** |  |  |  |  |  |  |  |  |  |  |  |  |  |  |  |
| Male | 1 | 1 | 1 | 1 | 1 | 1 | 1 | 1 | 1 | 1 | 1 | 1 | 1 | 1 | 1 |
| Female | 0.39 | 0.36 | 0.42 | 0.38 | 0.35 | 0.41 | 0.40 | 0.37 | 0.43 | 0.58 | 0.54 | 0.63 | 0.65 | 0.60 | 0.70 |
| **Age** |  |  |  |  |  |  |  |  |  |  |  |  |  |  |  |
| 25-64 | 1 | 1 | 1 | 1 | 1 | 1 | 1 | 1 | 1 | 1 | 1 | 1 | 1 | 1 | 1 |
| 65-79 | 11.56 | 10.37 | 12.89 | 13.25 | 11.93 | 14.72 | 13.49 | 12.15 | 14.96 | 7.00 | 6.32 | 7.76 | 6.88 | 6.21 | 7.61 |
| **Comorbidities** |  |  |  |  |  |  |  |  |  |  |  |  |  |  |  |
| No | 1 | 1 | 1 | 1 | 1 | 1 | 1 | 1 | 1 | 1 | 1 | 1 | 1 | 1 | 1 |
| Yes | 3.42 | 3.06 | 3.81 | 3.27 | 2.95 | 3.63 | 3.33 | 3.01 | 3.69 | 2.09 | 1.89 | 2.31 | 2.11 | 1.92 | 2.33 |
| **Administered COVID-19 test** |  |  |  |  |  |  |  |  |  |  |  |  |  |  |  |
| No |  |  |  | 1 | 1 | 1 | 1 | 1 | 1 | 1 | 1 | 1 | 1 | 1 | 1 |
| Yes |  |  |  | 2.28 | 2.10 | 2.47 | 0.42 | 0.35 | 0.50 | 0.48 | 0.40 | 0.56 | 0.55 | 0.47 | 0.64 |
| **Positive COVID-19 test** |  |  |  |  |  |  |  |  |  |  |  |  |  |  |  |
| No |  |  |  |  |  |  | 1 | 1 | 1 | 1 | 1 | 1 | 1 | 1 | 1 |
| Yes |  |  |  |  |  |  | 31.13 | 26.04 | 37.21 | 1.60 | 1.35 | 1.91 | 1.37 | 1.16 | 1.62 |
| **COVID-19 hospital admission** |  |  |  |  |  |  |  |  |  |  |  |  |  |  |  |
| No |  |  |  |  |  |  |  |  |  | 1 | 1 | 1 | 1 | 1 | 1 |
| Yes |  |  |  |  |  |  |  |  |  | 566.81 | 521.32 | 616.28 | 425.55 | 388.80 | 465.76 |
| **COVID-19 ICU admission** |  |  |  |  |  |  |  |  |  |  |  |  |  |  |  |
| No |  |  |  |  |  |  |  |  |  |  |  |  | 1 | 1 | 1 |
| Yes |  |  |  |  |  |  |  |  |  |  |  |  | 2.81 | 2.60 | 3.05 |
